# A modular spring-loaded actuator for mechanical activation of membrane proteins

**DOI:** 10.1038/s41467-022-30745-2

**Published:** 2022-07-28

**Authors:** A. Mills, N. Aissaoui, D. Maurel, J. Elezgaray, F. Morvan, J. J. Vasseur, E. Margeat, R. B. Quast, J. Lai Kee-Him, N. Saint, C. Benistant, A. Nord, F. Pedaci, G. Bellot

**Affiliations:** 1 Université de Montpellier, Centre de Biochimie Structurale, INSERM, CNRS, 34090 Montpellier, France; 2 Université de Montpellier, Institut de Génomique Fonctionnelle, INSERM, CNRS, 34090 Montpellier, France; 3 CRPP, CNRS, UMR 5031, Université de Bordeaux, Pessac, France; 4 IBMM, Université de Montpellier, CNRS, ENSCM, Montpellier, France; 5 PHYMEDEXP, Université de Montpellier, CNRS, INSERM, 34090 Montpellier, France

**Keywords:** Nanostructures, Nanoscale biophysics, Membrane proteins

## Abstract

How cells respond to mechanical forces by converting them into biological signals underlie crucial cellular processes. Our understanding of mechanotransduction has been hindered by technical barriers, including limitations in our ability to effectively apply low range piconewton forces to specific mechanoreceptors on cell membranes without laborious and repetitive trials. To overcome these challenges we introduce the Nano-winch, a robust, easily assembled, programmable DNA origami-based molecular actuator. The Nano-winch is designed to manipulate multiple mechanoreceptors in parallel by exerting fine-tuned, low- piconewton forces in autonomous and remotely activated modes via adjustable single- and double-stranded DNA linkages, respectively. Nano-winches in autonomous mode can land and operate on the cell surface. Targeting the device to integrin stimulated detectable downstream phosphorylation of focal adhesion kinase, an indication that Nano-winches can be applied to study cellular mechanical processes. Remote activation mode allowed finer extension control and greater force exertion. We united remotely activated Nano-winches with single-channel bilayer experiments to directly observe the opening of a channel by mechanical force in the force responsive gated channel protein, BtuB. This customizable origami provides an instrument-free approach that can be applied to control and explore a diversity of mechanotransduction circuits on living cells.

## Abstract (web summary)

Studies on mechanotransduction are limited by our ability to apply low range forces to specific mechanoreceptors on cell membranes. Here the authors report the Nano-winch, a programmable DNA origami-based molecular actuator, to manipulate multiple mechanoreceptors in parallel by exerting piconewton forces.

## Introduction

Mechanical forces at the nanoscale are essential for numerous biological processes. To directly detect these forces, cells use molecular mechanosensors such as the cytoskeleton, molecular motor proteins, and cell surface receptors to translate piconewton (pN) scale mechanical forces into biochemical signals^1^. Investigating these molecular systems has been facilitated by the development of techniques that interface with molecules, such as atomic force microscopy, magnetic, and optical tweezers^2^. Significant discoveries have been made using these techniques; however, they are laborious, limited by low throughput and molecular specificity, and difficult to operate at a low pN range of force suitable for a majority of biological effects. In addition they require connection of the biomolecule to long tethers or to operate near a surface. DNA nanotechnology is one promising approach, able to interface with living cells, as well as to measure and exert pN scale forces. Synthetic DNA has been at the forefront of probes designed to interrogate mechanosensitive cell surface receptors using single-molecule fluorescence^1^. Moreover, it allows the fabrication of sophisticated DNA origami nanodevices with complex geometries, programmable mechanical properties, and precise positioning of molecular functionalities^3,4^.

DNA origami methods have previously been applied to decrease experimental noise in optical tweezers^5^ and introduce massive parallelization and high data throughput in single-molecule fluorescence^6^. DNA origami are now being encoded for creating machine-like nanostructures that can be mechanically controlled through the hybridization of oligonucleotides, thermal fluctuations, and electrical fields^7–10^. The next step is to apply this technique to target specific cell surface receptors and generate mechanical forces to elicit biological responses in massive parallelization^11^.

Using the DNA origami method, we report the creation of a modular molecular device called the Nano-winch. The Nano-winch is programmed to exert linear mechanical stresses on specific, individual membrane proteins, applicable to cells. The shape of the Nano-winch is inspired by prismatic joints to generate linear motion, to land on membrane surfaces, to bind a specific cell surface receptor, and then act as a molecular spring to exert tuneable stress.

## Results

### Design and assembly of the Nano-winch

**Fig. 1:**
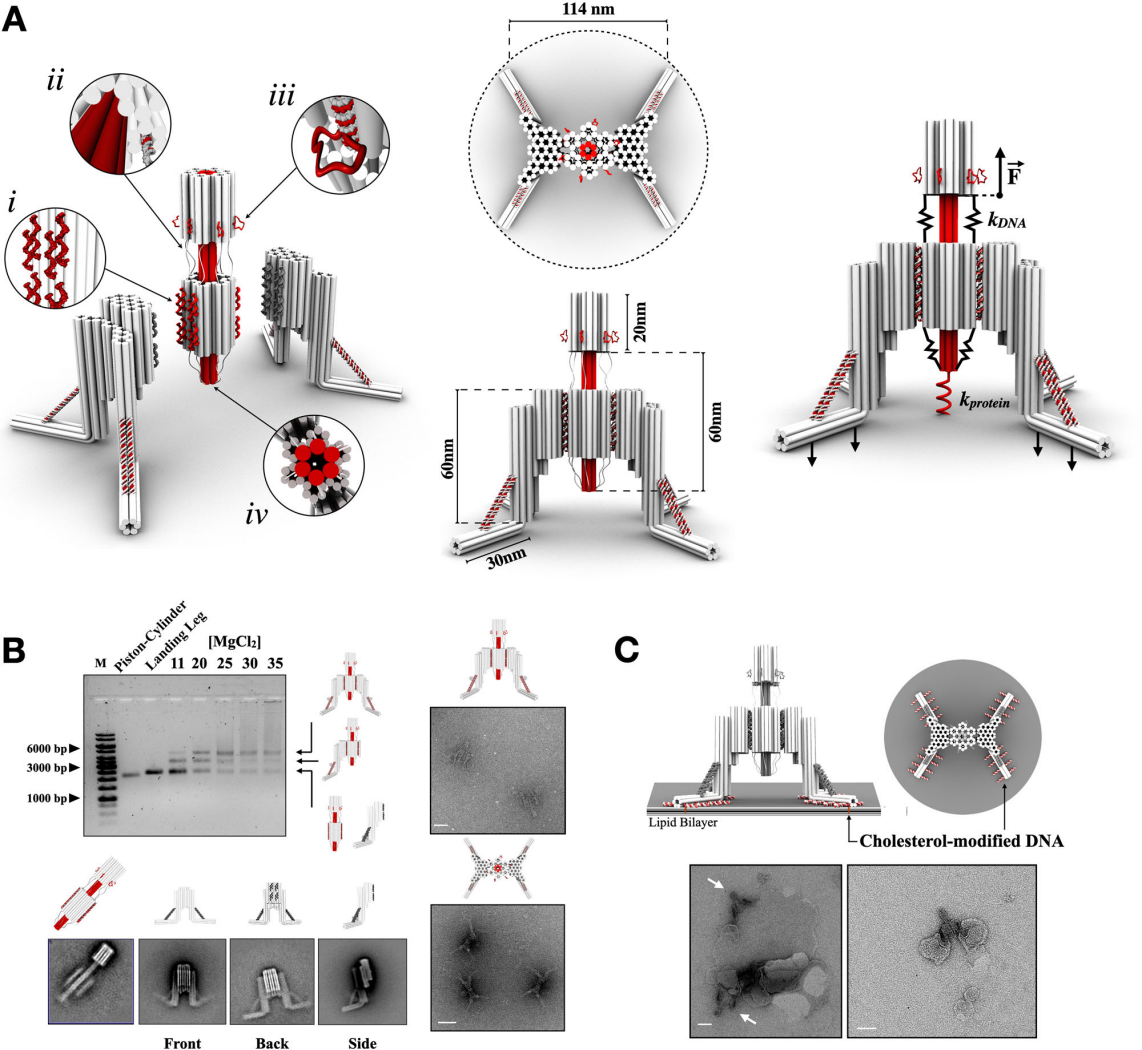
Design and assembly of a DNA-based Nano-winch. **A** Schematic illustration of the assembly strategy of the DNA-based Nano-winch. Double-helical DNA domains are represented by cylinders and are packed on a honeycomb lattice. The Nano-winch comprises two origami in a 1:2 trimer, a central Piston-cylinder and two Landing Legs. The central piston-cylinder core origami has eight strands to anneal to the inner face of the Landing Leg origami (i). To prevent toppling, two ~30 nm six-helix bundles project at 90° from the origami landing legs and 45° away from each other to lay parallel to a surface and maximize area coverage to retain an upright position. They are reinforced with a dsDNA strut. Single-stranded DNA connectors link the top and bottom of the cylinder to the backstop and the piston tip, respectively (ii). These single-stranded DNA connectors loops act as entropic springs with stiffness *k*DNA and exert defined force which is mechanically translated through the rigid origami to the tip of the piston coupled to a molecular target with stiffness *k*protein. The length of these connectors can be adjusted by storing the excess scaffold in reservoir loops on the backstop (iii). The tip of the piston positions up to three ligand moieties targeting specific cell surface receptors (iv). Extension of the Piston results in equivalent and opposing compressive force through the Landing Legs. **B** 1.0% agarose gel with 11 mM MgCl_2_ on which the following samples were electrophoresed: M, 1-kb ladder, Piston-cylinder monomer, Landing Leg monomer, and Piston-cylinder incubated with two-fold molar excess of Landing Legs with a concentration gradient from 11 mM to 35 mM MgCl_2_. Schematic representations and reference-free class averages from single-particle TEM micrographs of individual components (particle sets available in Supplementary Figs. 5, 8, and 9). Fully assembled Nano-winch particles were visualized both laterally and from above. **C** Each landing leg can be modified with eight cholesterol moieties, for a total of 32-modifications. Example TEM images of Nano-winches functionalized with cholesterol moieties adhering to small unilamellar vesicles through the landing legs. Distortions of liposomes is an effect of the process of sample preparation for negative stain TEM. White bars represent 50 nm. All TEM analyses were conducted at least three times for each sample. Source data are provided as a Source Data file.

Our Nano-winch comprises two origami in a 1:2 trimer: a central piston-cylinder and two landing legs (Fig. 1A). The piston-cylinder consists of two domains: a 60 nm long six-helix bundle (6-HB) piston topped with a 20 nm backstop, and a 25 nm cylinder folded concentrically around the piston with a ~2 nm gap between them (Supplementary Figs. 1–2), minimizing friction and allowing the two domains to slide freely. Six single-stranded scaffold connectors, up to 97 nt long, link the backstop to the top of the cylinder. Six additional 97 nt connectors connect the bottom of the cylinder to the piston tip (Fig. 1A)^7^. These single-stranded DNA connectors loops act as entropic springs with constant stiffness *k*DNA and exert defined stresses in the low pN range, which is mechanically translated through the rigid origami to the tip of the piston coupled to a molecular target with a spring of constant stiffness *k*protein, a simplified model of proteins as linear springs as opposed to real proteins. The length of these connectors can be adjusted by substituting a small number of staple strands, storing the excess scaffold in reservoir loops on the backstop so the machine can be tuned cost-efficiently with defined distances and forces increasing the range of potential application. The tip of the piston positions up to three ligand moieties targeting specific cell surface receptors with precision to control both the distance between ligands (4 nm) and ligand stoichiometry allowing targeting of a single mechanoreceptor or several simultaneously at controlled distance across the ~6 nm wide 6-HB piston. Two origami landing legs, which attach to opposite sides of the cylinder through mirrored single-stranded anchors, bring the device to rest on a membrane surface, anchor it in place, and prevent toppling. The origami landing legs provide much of the rigidity of the device, with a micron scale persistence length of 3.8 μm^12^, preventing torsion on the membrane surface and hindering bending of the device during operation. To prevent toppling, two ~30 nm 6-HBs project at 90° from the device and 45° away from each other to lay parallel to a surface and maximize area coverage to retain an upright position. These projected 6-HBs are each reinforced with a dsDNA strut to prevent compression or bending in the landing leg (Fig. 1A top view). Each 6-HB has eight anchor strands that can be functionalized with specific ligand-conjugated oligonucleotides, such as cholesterol, to localize the Nano-winch to the bilayer and retain orientation of the device (Fig. 1C).

Individual origami components were folded, purified by electrophoretic migration, and visualized by transmission electron microscopy (TEM) (Supplementary Figs. 3, 5, 7–9). Averaged TEM particles of the piston-cylinder origami show the concentric folding of the cylinder around the piston. Likewise, averages of the landing leg origami show the front, back, and side of the origami with desired folding of the 6-HB projections with the reinforcing strut. Incubating the piston-cylinder:leg origami with a molar ratio of (1:2.2) and an increasing concentration of magnesium from 11 mM to 35 mM enabled the assembly of the ensemble. Both individual origami migrate to a similar position on agarose gels, but samples incubated with the landing leg origami yielded two additional slower migrating bands (Fig. 1B). The upper band was purified and confirmed by TEM to be the trimeric ensemble (Fig. 1B, Supplementary Figs. 11–13). Side views of the Nano-winch confirm the proper annealing of both landing legs to opposing sides of the cylinder domain. Top views of the Nano-winch show the expected arrangement of the landing leg projections at 90°, ideal for adhering to a membrane surface (Fig. 1B, Supplementary Fig. 13). To test the ability of the device to be addressed to a lipid membrane, we modified each landing leg with eight cholesterol moieties, for a total of 32-modifications (Supplementary Figs. 14 and 15). After incubation with small unilamellar vesicles (SUVs), TEM micrographs reveal the adhesion of the device in the desired orientation. Interaction with smaller SUVs confirm that the device specifically binds to vesicles on the functionalized landing legs (Fig. 1C and Supplementary Fig. 16).

### Characterization of the Nano-winch

We then explored the capability of the device to exert mechanical stresses on a membrane protein. Our approach has been to take advantage of the single-stranded DNA connectors behaviour which functions as an entropic spring^13–15^ and can drive the piston away from the surface. We term this the “autonomous” mode of the Nano-winch, in which it exerts forces without outside signals.

**Fig. 2:**
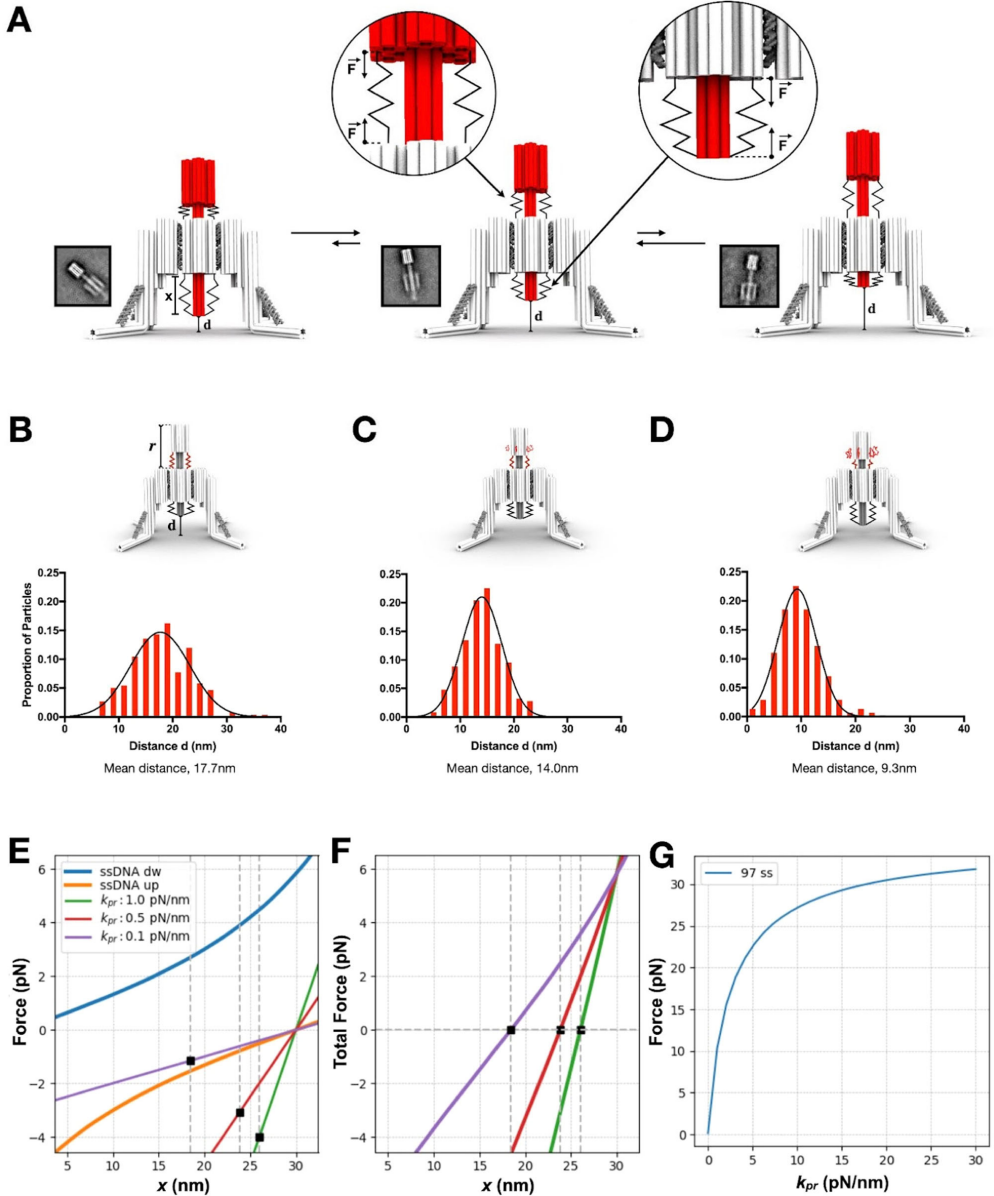
Autonomous single-stranded DNA Nano-winch characterization. **A** Fully assembled trimer with single-stranded 97 nt connectors exists in an equilibrium state between ~5–25 nm with corresponding reference-free class average calculated from single-particle TEM micrographs. Measured distance *r*, correlates directly to the distance *d* between the surface and the tip of the piston. Nano-winches tuned with (**B**) 97 nt, (**C**) 60 nt, and (**D**) 30 nt connector strands and the corresponding distance *d* distributions and reference-free class averages (particle sets available in Supplementary Figs. 20, 22). The length of the connectors are adjusted by storing the excess scaffold in reservoir loops on the backstop, in red. **E** The forces exerted by a single (*n* = 1) top (orange) and bottom (blue) 97 nt ssDNA molecule are plotted as a function of extension *x* defined as the distance between the bottom of the piston and the bottom of the cylinder. The force of the linear spring is plotted for the values of *k*protein (kpr) = (0.1, 0.5, 1) pN/nm. **F** Total force as a function of x for the three values used for kpr. The corresponding values of the equilibrium state of x, x_eq_, are shown as vertical gray dashed lines in both a) and b) (Supplementary Note 1). **G** The total force applied on the linear spring as a function of kpr, when *n* = 6 molecules of 97 nt are present at the top and at the bottom of the structure.

We started by folding the device with six 97 nt long connectors between the backstop and cylinder. Measurements of the distance *r* between the backstop and the cylinder correlates directly to the distance d retracted on a target membrane protein as depicted in Fig. 2B and Supplementary Fig. 18. Measurements of the distance *r* on TEM give a distribution of distance d between ~5–30 nm with a mean of 17.7 nm (Fig. 2A, Supplementary Figs. 17–18). This distance distribution reveals the ability of the piston to scan the membrane surface to recruit target proteins and then extend perpendicularly to the membrane to a maximum d of ~30 nm (Fig. 2B). To adjust the distance distribution, we tuned the length of the corresponding reservoir loops to change this extension. Two additional designs with 60 nt and 30 nt connectors were assembled and evaluated by TEM (Fig. 2C, D). The mean distribution distances of 14.0 nm and 9.3 nm were evaluated for the 60 nt and 30 nt device, respectively, from TEM single-particle analysis (Supplementary Figs. 19–22). To estimate the amount of force applied by the 97 nt connector device in autonomous mode, we first approximated the countervailing top and bottom ssDNA connectors as entropic springs using a Worm Like Chain model (WLC) with the device attached to a simplified target molecular spring, *k*_protein_, with varying stiffness (Supplementary Note 1, Supplementary Fig. 23). For a typical molecular target with a spring of constant stiffness ranging from 0.1 to 20 pN/nm^16,17^, we find the force applied by the nano-winch spans the range from 1.6 pN to 30 pN depending on *k*protein (Fig. 2E–G). This mechanical behaviour is explored further using coarse-grained molecular dynamics and Monte Carlo simulations on oxDNA software^18^, Supplementary Note 2, Supplementary Figs. 24–25) at experimental conditions with a constant 23 °C to reduce thermal fluctuations on the Nano-winch and protein and with salt concentrations matching those of folding conditions to retain structural integrity. Simulations indicated a tension in a range similar to the WLC model. An estimate of the force exerted on the linear spring as a function of *k*protein is given in Supplementary Fig. 25. Force generated by the entropic spring behavior of the connectors in the autonomous mode is translated through the highly rigid body^12^ of the Nano-winch to the target molecule at the tip of the piston. As the piston exerts force perpendicularly to the membrane, the landing legs also distribute an equal and opposing force across a membrane that retains sufficient tension to keep the device moored on the surface (Fig. 2A). The force exerted by the device is affected by factors in the environment, including membrane deformation under the landing legs. Mey et al.^19^ showed that typical membrane indentation curves are linear and, with piston of the Nano-winch, are likely to follow ~1.0Å of vertical displacement with each pN of force exerted by the device (Supplementary Note 4).

### Nano-winch autonomous activation of mechanoreceptors on cells

To explore this mechanical behavior and the functionality of the device on cells, we used the autonomous Nano-winch device to interrogate integrin membrane surface receptors on MCF-7 human breast cancer cells, which have been shown to express high concentrations of β1 integrin and innumerous small integrin clusters^20^. Upon ligand binding and mechanical stress, integrins shift conformation from inactive at ~11 nm to active, elongated up to ~19 nm from cell surface^21^. Reasonable estimates of the forces exerted on single and early activated integrins are in the range of *~*1 and 15 pN^22–24^. These relatively low pN tensions are transmitted to the cytoskeleton to stimulate actin polymerization, and recruit various protein adhesions such as talin, paxillin, and focal adhesion kinase (FAK)^25^ that in turn induce biochemical signals such as FAK phosphorylation^26^. Recent studies have shown a direct proportional, linear relationship between force exerted on integrins and FAK phosphorylation^27^. The Nano-winch provides a tool for mechanical activation of membrane mechanoreceptors such as integrins, in this case on cells in suspension, in contrast to previous studies on substrate^26,27^. We used the 97 nt Nano-winch which has a ~20 nm dynamic range d-distance allowing the tip to explore the membrane surface, to recruit the inactive integrin, and to sufficiently accommodate the height of active integrin. A synthetic cyclic-peptide (cyclic Arg-Gly-Asp, cRGD) was engineered to mimic integrin ligand (Supplementary Figs. 26–28)^28^ and three were clustered approximately 4 nm apart on the piston tip (Supplementary Fig. 29) to promote cRGD-integrin engagement via statistical rebinding^4,29^.

**Fig. 3:**
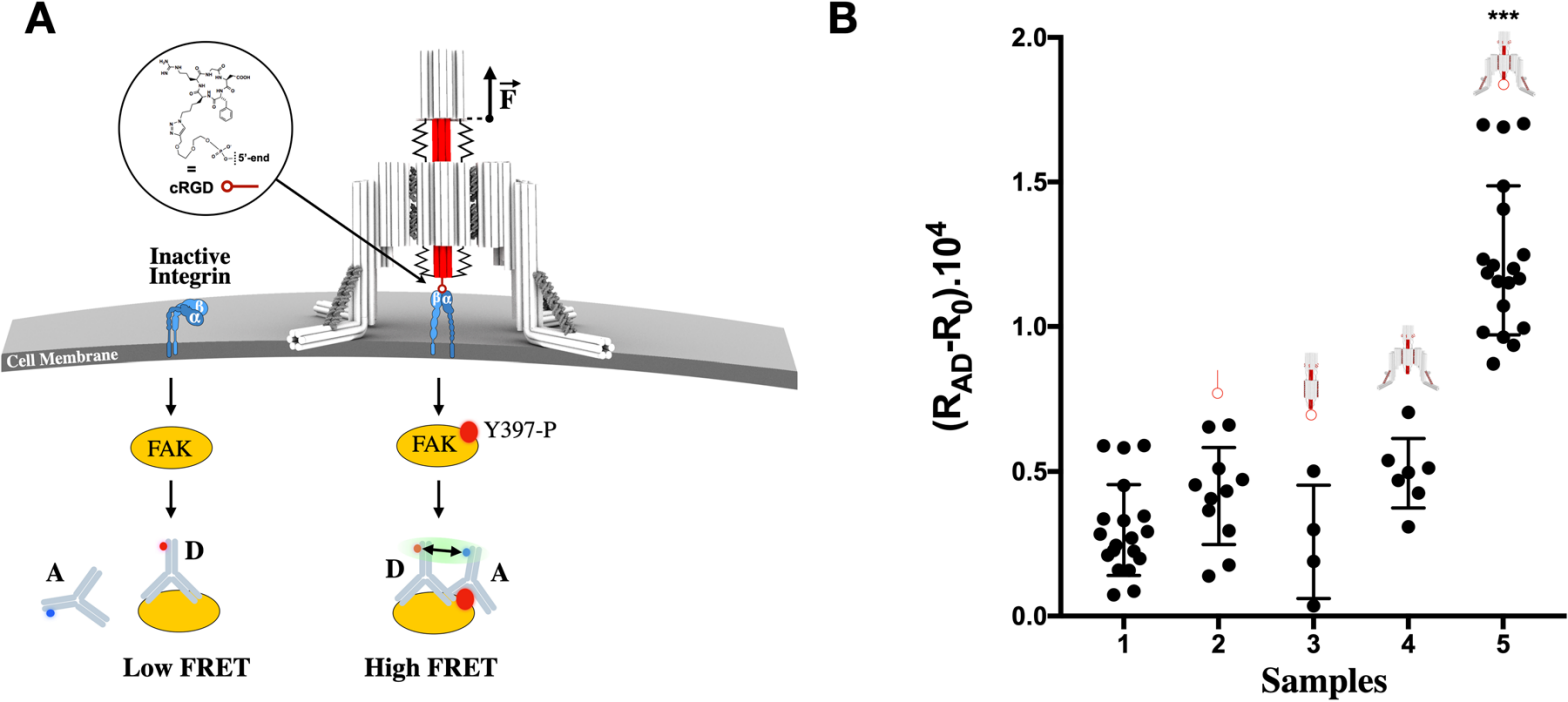
Autonomous DNA Nano-winch activation of integrin signalling. **A** The transmembrane receptor integrin (blue) exists as a compact **αβ** heterodimer. Integrins transmit applied mechanical stresses, between 1 and 15 pN, and recruits additional proteins to assemble focal adhesions including Focal Adhesion Kinase (FAK), which becomes phosphorylated at residue Y397 after mechanical stimulation of integrin. Addition of two antibodies with donor, D, and acceptor, A, labels allows detection of phosphorylated FAK in a LRET assay. Both antibodies bind to phosphorylated FAK (Y397-P) eliciting a detectable high LRET signal, whereas only a single antibody binds in the absence of phosphorylation yielding a low LRET signal. **B** MCF-7 cells in suspension were 1, left untreated control, 2, incubated with RGD conjugated oligonucleotide, 3, incubated with cRGD functionalized Piston-cylinder origami, 4, incubated with non-functionalized Nano-winches, 5, incubated with cRGD functionalized Nano-winch. Cells were then lysed and FAK phosphorylation. The background signal, R_0_, of antibodies alone was subtracted from the signal of lysed cells in experimental and control conditions calculated from ratios of acceptor and donor fluorescence intensities, R_AD_. Results are the average of at least three independent experiments. Error bars represent the standard deviation, statistical significance was determined by one-way analysis of variance with comparison to the untreated control (***P < 0.001). Source data are provided as a Source Data file.

The mechano-chemical signal conversion was monitored using the cytoskeletal protein FAK that auto-phosphorylates at position Y397 upon integrin activation under force exertion^30^ (Fig. 3A). This finding is supported by other recent studies showing a direct relationship between the force applied to integrin and FAK Y397 phosphorylation^26,27^, although with cells on a substrate, whereas ours are in suspension. We examined mechanical stimulation of integrin by measuring Luminescence Resonance Energy Transfer (LRET) between donor and acceptor fluorescently labelled anti-FAK antibodies which specifically bind FAK and phosphorylated FAK, respectively. cRGD-oligo alone and cRGD functionalized piston-cylinder origami without landing legs showed a low LRET acceptor-donor (A-D) emission ratio response, R(A/D) - R0, found to be around 0.40 × 10^4^. Fully assembled Nano-winch lacking cRGD functionalization or functionalized with a RGE peptide as a control also showed low LRET responses, R(A/D)— R0 = 0.49 × 10^4^ ± 0.12 × 10^4^ and 0.27 × 10^4^ ± 0.01 × 10^4^, respectively (Supplementary Fig. 30). However, only fully assembled Nano-winch functionalized with cRGD peptides and incubated with MCF-7 cells elicited a high LRET response, R(A/D)— R0 = 1.23 × 10^4^ ± 0.26 × 10^4^ (Fig. 3B, Supplementary Figs. 31–32). This demonstrates that only a cRGD-functionalized fully assembled Nano-winch can interact specifically with integrin cell receptors and generate linear motion and a corresponding compressive force on the membrane through the landing legs. The average number of Nano-winches on cell surfaces was quantified by flow cytometry as an average of 9,200 ± 312 Nano-winches per cell (Supplementary Fig. 33). Under optimal MCF-7 cell densities that overexpress integrins and optimal incubation time (Supplementary Figs. 31–32), the numbers of Nano-winches per cell generated sufficient tension to stimulate detectable downstream phosphorylation of FAK.

### Remotely controlled Nano-winch activation

**Fig. 4:**
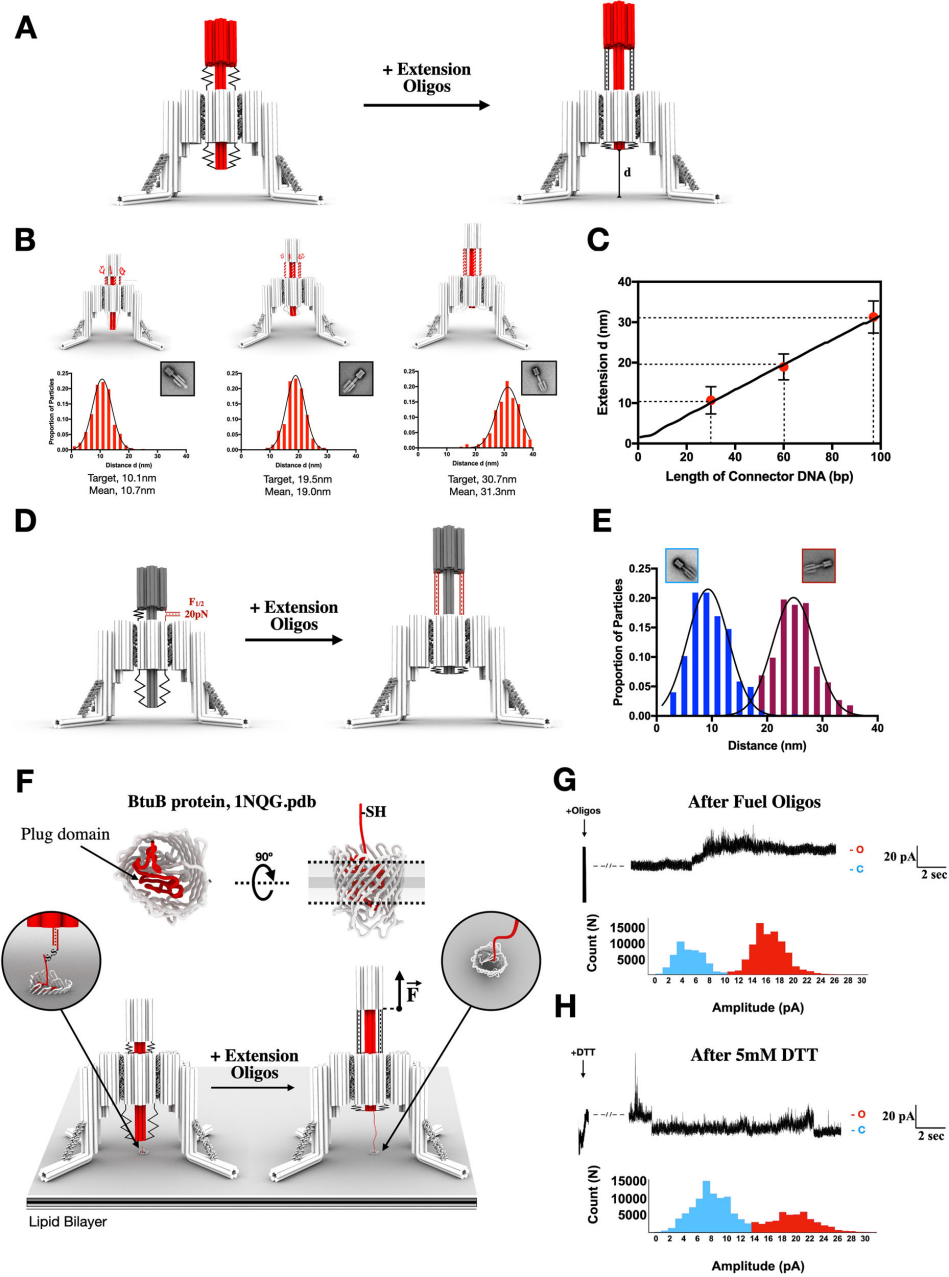
Remotely activated double-stranded DNA Nano-winch characterization and activity. **A** Addition of extension oligonucleotides complementary to the connector strands between the backstop and cylinder ratchets the piston from the membrane surface. Annealing of the oligonucleotides to these connectors transitions them from single-stranded, with a persistence length of ~1 nm, to double-stranded, which is fifty times stiffer, at 50 nm. **B** Nano-winches tuned with 30-bp, 60-bp, and 97-bp connectors between the backstop and cylinder with corresponding distance d measurements and reference-free class averages (particle sets available in Supplementary Figs. 33, 35, and 37). **C** The mean distance extension of 30-bp, 60-bp, and 97-bp connectors (red dots) overlaid on the WLC model of Piston-cylinder extension (black line) enables the user to rationally define the connectors length for a required distance *d*. Error bars represent standard deviation. **D** Nano-winch was folded with a DNA hairpin tethering the backstop to the cylinder (red strands) requiring F1/2 of approximately 20pN to unzip. This hairpin serves as a benchmark to cross-validate the force applied by annealing of the extension oligonucleotides estimated by coarse-grained molecular dynamics simulation. **E** The distance d of tethered Piston-cylinders with the hairpin were measured before (blue) and after (red) incubation with extension oligonucleotides (particle sets available in Supplementary Figs. 39–40). **F** BtuB is a mechanically gated β-barrel channel occluded by a globular plug domain. The N-terminal 49 residues (red) are dislocated to form a channel and a N- terminal linker was engineered with a cysteine residue into the protein to serve as a linker for conjugation with a thiolated oligonucleotide. Addition of extension oligonucleotides retracts the piston and unfolds part of the plug domain to open a channel. **G** Selected traces and corresponding trace count histogram of BtuB-oligo reconstituted into planar lipid bilayers with Nano-winches after addition of 400 nM extension oligonucleotides. Closed channels (blue) transitioned to an open state (red) over two seconds after addition of 400 nM extension oligonucleotides. **H** Reducing the connection between BtuB and the Nano-winch allowed the plug domain to refold and transition from an open channel back to a closed state. Breaks in the traces represent preparations for thoroughly mixing the cis compartment. Arrows on the graphs indicate where the extension oligonucleotides and the DTT reducing agent solutions are added. Source data are provided as a Source Data file.

The autonomous mode of the Nano-winch exerts a ~1–25 pN tension on the target protein depending on the spring constant *k*protein, which covers a large number of biological processes^1^. However, other molecular systems may require finer control of distance *d* extension and higher amounts of force to elicit a response. To achieve this, we explore the capabilities of the Nano-winch to be remotely controlled by addition of oligonucleotides complementary to the backstop-cylinder connector loops. We term this the “remotely activated” mode of the Nano-winch. Annealing of the oligonucleotides to these connectors transitions them from single-stranded, with a persistence length (lP) of ~1 nm^31^, to double-stranded DNA, which has comparatively greater stiffness with a lP of ~50 nm at sodium concentrations above 10 mM^32^. The Nano-winch reservoir loops allow us to tune the length of the connectors with a length intermediate between these two lP. We tested three tuned lengths of the connectors to ascertain their extension distances, 30-bp, 60-bp, and 97-bp. This corresponds to movement of the piston tip by an anticipated distance d on a target membrane protein of 10 nm, 20 nm, and 33 nm respectively (Fig. 4A).

The single-stranded version of each origami was folded, purified, then incubated with extension oligonucleotides complementary to the connector strands to “remotely activate” Nano-winches. Measurements under TEM show ratcheting of the backstop away from the cylinder with mean extension distances d of 10.7 nm, 19.0 nm, and 31.3 nm for each device, respectively (Fig. 4B, Supplementary Figs. 34–39). The experimentally verified extension distances corresponded with the anticipated extension distances modelled from the WLC model (Fig. 4C). Multiple double-stranded connectors in parallel increases the probability of precisely positioning the piston to the desired extension distance (Supplementary Note 3, Supplementary Fig. 40). This model accurately predicts the extension distance *d* of the remotely activated Nano-winch from the double stranded connector lengths and enables the user to rationally define the connectors length for a required distance *d*. To estimate the amount of force applied by the Nano-winch, we performed coarse-grained molecular dynamics and Monte Carlo simulations of double stranded connectors, with respectively 60 and 97 nucleotides, using oxDNA software. For a molecular target with a spring of constant stiffness *k*protein ranging from 0.1 to 20 pN/nm, we find the force applied by the Nano-winch on the surface spanning the range from 1.7 pN to 80 pN (Supplementary Figs. 23–25). An estimate of the force exerted on the linear spring as a function of *k*protein is given in Supplementary Figs. 24–25. For weak deformations from the equilibrium configuration, the force exerted by the Nano-winch is proportional to the effective stiffness. Our simulations show an effective stiffness of 8.0 × 10^−3^ N/m for double stranded and 3.0 × 10^−3^ N/m for single-stranded 97-nucleotide connectors. Note that this value could be an overestimation, as several degrees of freedom, such as relative tilting or rotation of cylinder versus backstop, were ignored.

To experimentally validate that ds-Nano-winch generates tens pN-scale forces, we tested the Nano-winches’ ability to mechanically rupture a DNA hairpin. We folded a piston-cylinder with 97 nt long connectors with a DNA hairpin requiring a known benchmark of force F1/2, which is defined as the equilibrium force of ~20 pN^33^ at which the hairpin spends half of its time in an unzipped state as illustrated in Fig. 4D. Measurements of these tethered piston-cylinders before and after incubation with 97 nt extension oligonucleotides on TEM show extension of the device (Fig. 4E). Also see Supplementary Note 3, representative TEM micrographs and the averaged image from single-particle (Supplementary Figs. 41–42). The deformation from equilibrium imposed by the presence of the hairpin can be estimated to be 5.3 nm (Fig. 4B, E). In the linear approximation, this corresponds to a 8.0 × 10^−3^ N/m × 5.3 nm = 42pN force, which is larger than the hairpin F_1/2_. This shows that the Nano-winch hybridization generates sufficient linear mechanical force to unzip this DNA hairpin^15^.

We tested the remote activation of Nano-winches to mechanically open a channel. TonB-dependent receptors are β-barrel proteins with a globular plug domain occluding passage through the central channel. These proteins recruit the inner membrane protein complex TonB-ExbB-ExbD to mechanically unfold a force-labile region of the plug by approximately 20 nm to form a ~13-Å channel^34^, (Supplementary Fig. 43). A TonB- dependent receptor, BtuB, was engineered with an accessible ~8 nm linker on the plug domain containing a cysteine, allowing covalent conjugation of a thiolated- oligonucleotide complementary to an anchor strand positioned on the tip of the piston (Fig. 4F) and permits detachment of the Nano-winch upon addition of the reducing agent. The plug domain of BtuB refolds after channel opening^35^ and will presumably refold after detachment from the Nano-winch. After BtuB purification, coupling between BtuB and thiolated-oligo leads to ~80% yield (Supplementary Fig. 44). BtuB-oligo was separated from excess oligo using size exclusion chromatography (Supplementary Fig. 45) and then reconstituted into planar lipid bilayers to detect channel formation via conductance across the membrane.

The presence of 4 M urea elicited channel activity from the BtuB-oligo complex (Supplementary Fig. 46), confirming that plug denaturation and integrity of the engineered BtuB is similar to wild-type protein^36^. To accommodate both the 20 nm extension distance^34^ and the ~8nm N- terminal linker, we, again, employed the Nano-winch with a 33 nm extension from the 97 nt connectors. Nano-winches bearing complementary anchors to the BtuB-oligo were added to the cis compartment with no modification of the conductivity at 4 ± 4pA (Supplementary Fig. 47). After 20 min complementary oligonucleotides were added in the cis compartment. Channel opening events shifted the conductivity from the closed state to an open state of 16 ± 4pA, likely reflecting the opening of the BtuB channel by the Nano-winch (Fig. 4G). Addition of dithiolthreitol to the *cis* chamber resulted in closing events reverting from an open state of ~20 ± 6pA to the closed state of ~8 ± 6pA (Fig. 4H). We interpret this as the reduction of the disulfide bond between BtuB and the thiolated oligo, detaching the Nano-winch and relaxing the plug domain to close the β- barrel. Control experiments in the absence of Nano-winch or without the addition of extension oligos failed to elicit increased conductivity (Supplementary Fig. 48).

## Discussion

Our DNA-based Nano-winch is a tuneable, adjustable nanodevice able to interface directly with molecular systems and cells to elicit biochemical effects via mechanical tension. This provides a method that expands the palette of techniques to investigate mechanical-chemical communication of cells. The components of the machine are simple to assemble into a complete actuator, robust enough to be used in a variety of systems and able to adjust the applied distance and tension. Functionalization of the legs with cholesterol is a general method to attach to a membrane bilayer, but it is possible to functionalize with other ligands, such as antibodies or nanobodies, to target specific cell types. Furthermore, functionalization of the piston with controlled stoichiometry and vicinity will provide opportunities for interrogating different membrane proteins and multivalent interactions. The DNA origami method produces billions of individual, functionalized nano-devices able to work in parallel to mechanically activate membrane proteins. In the near future the Nano-winch could be combined with existing techniques, such as super-resolution microscopy, to explore the mechanical force landscape of living cells. Future improvements to the Nano-winch could direct it to particular microdomains of the cells to help investigate specific cell mechanical processes. The Nano-winch operates at a low pN range of force exertion suitable for a majority of biological effects^1,37^. To achieve higher tension exertion required for other biological investigations, our device can be modified to generate actuation through cooperative oligonucleotide annealing^38^, external magnetic^39^, or electric fields and light^10,40^. Efforts to stabilize DNA nano-structures in biological environments using covalent cross-linking or chemical modifications are ongoing^41,42^. A Nano-winch hardened against degradation in a biological environment could target specific cell types and receptors to investigate the effects of mechanical activation on, for example, different processes during specific stages of growth or development. We envision that in the near future our device could complement investigations into mechanotransduction systems in situations inaccessible to existing techniques.

## Methods

### Oligonucleotides

Desalted staple oligonucleotides were purchased from either Eurofins or Integrated DNA Technologies (IDT). Scaffold p7560 was purified from the M13 bacteriophage replicated in XL1 blue strain *Escherichia coli* (Agilent, USA).

### Design and assembly of DNA origami devices

DNA structures were designed using the honeycomb lattice on caDNAno (v.2.0). Scaffold p7560 at 25 nM (Piston-cylinder Origami) or 50 nM (Landing Leg Origami) was combined with 200 nM of the corresponding staple strands in a buffer of 5 mM Tris-HCl, pH 8.0, 1 mM EDTA, 18 mM MgCl_2_. The origami were subjected to a thermal annealing ramp: 65 °C for 15 min, 60 °C to 40 °C − 1 °C per every 2 h, then held at 10 °C.

### Agarose gel analysis

DNA nanostructures were purified from 1% agarose (0.5X TBE, 45 mM Tris-borate, 1 mM EDTA, pH 8.3) supplemented with 11 mM MgCl_2_ and 0.5 mg·ml^−1^ Sybr SAFE. Samples were migrated on the gel for 3 h with a running buffer of 0.5X TBE, 11 mM MgCl_2_ 2.85 V·cm^−1^. Bands corresponding to properly folded DNA origami were excised and transferred to a DNA gel extraction spin column (Merck, France) centrifuged at 5000 g for 5 min at 4 °C.

### Assembly of the molecular Nano-winch

The full DNA Nano-winch origami was combined with a 2.2-fold molar excess of the Landing Leg origami separately purified from agarose gel in a buffer consisting of 0.5X TBE with 11 mM MgCl_2_. MgCl_2_ concentration was supplemented up to a final concentration of 20 mM and samples were then incubated at 37 °C for 10 h. Complete nanodevice assembly was evaluated by migration on 1% agarose and bands containing the fully assembled Nano-winch were excised and purified as described above. Incubation of the Nano-winch with cholesterol-conjugated oligonucleotide was performed at 37 °C for at least 30 min.

### Lipid Vesicle Preparation

Small unilamellar vesicles (SUVs) prepared from 1,2-dioleoyl-sn-glycero-3- phosphocholine (DOPC, Avanti Lipids) were solubilized in chloroform and evaporated under a nitrogen stream. Lipid film was then resuspended in a buffer containing 5 mM Tris-HCl, pH = 7.8, 100 mM NaCl, to 1 mg/ml final concentration. Lipid suspension was then extruded through 200 nm extrusion membranes (Avanti Lipids, U.S.A). Nano-winches functionalized with cholesterol were allowed to incubate with 0.25 mg/ml DOPC SUVs for at least 30 min at room temperature before visualization by electron microscopy.

### Transmission electron microscopy

Purified origami were visualized by adsorption onto glow-discharged carbon-coated grid (Quantifoil Micro tools GmbH, Germany), stained for 60 s with a 2% (w/vol) aqueous uranyl acetate (Merck, France) solution, and then dried with ashless filter paper (VWR, France). Observations of EM grids were carried out on a JEOL 2200FS FEG operating at 200 kV equipped with a 4k × 4k slow-scan CDD camera (Gatan Inc.). Two-dimensional class averages were computed using EMAN2 and ImageJ was used to measure distributions of the different DNA origami nanostructures.

### Cyclic RGD-oligonucleotide and RGE-oligonucleotide synthesis

The oligonucleotides were elongated from deoxyguanosine commercially available solid support on an ABI 394 DNA synthesizer according to standard phosphoramidite chemistry protocols (1 μmol scale). Detritylation was performed for 65 s using 3% TCA in CH2Cl2. For the coupling step: benzylmercaptotetrazole (0.3 M in anhydrous CH3CN) was used as the activator along with commercially available 2′-deoxyribonucleoside-O-2-cyanoethyl, N,N- diisopropylphosphoramidites (0.075 M in CH3CN, 30 s coupling time) or proparyl- diethyleneglycol phosphoramidite 11 (0.1 M in CH3CN, 60 s coupling time). The capping step was performed with acetic anhydride using commercially available solutions (Cap A: acetic anhydride:pyridine:THF 10:10:80 v/v/v, and Cap B: 10 % N-methylimidazole in THF) for 10 s. The oxidation step was performed with a standard, diluted iodine solution (0.1 M I2, THF:pyridine:water 90:5:5, v/v/v) for 15 s. Propargyl-oligonucleotide-functionalized CPG beads were introduced into a sealed vial and treated with conc. aq. ammonia (2 mL) overnight at 40 °C. The supernatant was withdrawn and evaporated. The 5′-propargyl modified oligonucleotide 3 was dissolved in water and then analysed by UV, HPLC and MALDI-TOF MS. The amount of three, determined by UV analysis at 260 nm, was (0.60 μmol, 60% yield). The HPLC purity at 260 nm was 79%. Analytical RP- HPLC retention time: 11.86 min on Macherey Nagel Nucleodur 100 − 3 C 18 ec column (length: 75 mm, ID: 4.6 mm) with a linear gradient of 1 to 25% of CH3CN in TEAAc 0.05 M pH 7. MALDI-TOF MS: m/z: [M-H] − : for C217H269N87O130P21 calcd.: 6826.48; found: 6826.93. CuAAC conjugation was performed on crude of 3. To 600 nmol of 3 in 240 μL of water was added the RGD azide 42 1200 nmol or RGE 42 4300 nmol (240 μL of a 5 mM solution in methanol, 2eq) 240 μL of 2 M TEAAc and ~0.1 mg of Cu0 nanopowder. The mixture was sonicated for 60 sec and then heated under microwaves assistance at 65 °C for 1 h. After centrifugation, the supernatant was withdrawn and saturated EDTA solution was added (500 μL). After 5 min, the solution was desalted by size exclusion chromatography (Nap10). The resulting solution was treated for 1 h with quadrapure IDA resin. The supernatant was then evaporated. The crude was purified by HPLC on a Macherey Nagel Nucleodur C18 HTec column (length: 250 mm, ID: 10 mm), using a linear gradient from 4 to 17% of CH3CN in 50 mM TEAAc pH 7 for 20 min, affording 200 nmol of 5 (33 % yield for RGD and 36% yield for RGE). Analytical RP-HPLC retention time for RGD: 13.49 min. MALDI-TOF MS: m/z: [M-H] − : for C244H308N98O137P21 calcd.: 7455.15; found: 7456.99.

### Integrin activation by the Nano-winch

Phospho-FAK (Tyr397) cellular assay: phospho-FAK assay is based on an Homogeneous Time- Resolved FRET (HTRF®) sandwich immunoassay format comprising two specific monoclonal anti-FAK antibodies, one labeled with Europium- cryptate (donor) and the other labeled with d2 (acceptor). Upon integrin pathway activation, FAK is phosphorylated on Tyr397 leading to a close proximity between the dyes. In this configuration, the excitation of the donor with a light source triggers a Luminescent Resonance Energy Transfer (LRET) towards the acceptor, which in turn fluoresces at a specific wavelength (665 nm). The specific signal modulates positively in proportion to phospho-FAK (Tyr397). FAK reagents were purchased at CisBio bioassays (Marcoules, France).

### Cell culture

MCF-7 cells (Michigan Cancer Foundation – 7) were grown in RPMI supplemented with 10% FBS (without antibiotics) at 37 °C, 5% CO_2_. Cells were split twice a week in 75 cm^2^ flasks. It must be specified that MCF-7 cells grow in clusters and are therefore difficult to dissociate with Versene, making the calculation of the number of cells inaccurate. MCF-7 cells were starved for twenty-four hours in RPMI before performing the experiment. The day of the assay, cells were detached with 5 ml Versene 1X solution (Gibco) for 10–15 min at 37 °C, 5% CO_2_. After adding 5 ml RPMI, cells were centrifuged for five minutes at 300 × g. The cell pellet was resuspended in RPMI in order to have the required number of cells (optimal was 10,000–20,000 cells, Supplementary Fig. 31) in 4 μl of medium. 4 μl of cells (10,000–40,000 cells/well) were plated in a 96-well white plate low volume (CisBio bioassays, Marcoules, France) and mixed with 4 μl of experimental and control DNA constructs stored in Folding Buffer 1X (5 mM Tris-HCl pH7.8, 1 mM EDTA, 18 mM MgCl_2_). FB1X was supplemented with 100 mM NaCl and 20 mM MgCl_2_ extemporaneously. Cells were then lysed by adding 4 μl of supplemented lysis buffer 4X and incubated for at least 30 min at room temperature under shaking. It was found that between five to 30 min after incubation of cells with cRGD-Nano-winches was sufficient to observe FAK phosphorylation (Supplementary Fig. 32). Finally, 4 μl of premixed antibody solution (vol/vol) prepared in the detection buffer were added to lysed cells and incubated 2 h at room temperature. HTRF readings were collected using a PHERAstar plate reader (BMG Labtech) at two specific wavelengths: 665 nm for the acceptor (A) and 620 nm for the donor (D). Phospho-FAK signal was assessed by calculating the ratio R(IA/ID)-R0. R0 is the background signal from the antibodies alone that was removed to the signal measured on lysed cells. The effect of Nano-winches was tested on MCF-7 cells in both autonomous and remote configurations. Nano-winches were incubated with MCF-7 cells in suspension prior to addition of 30 μM of extension oligonucleotides. No significant difference in FAK phosphorylation was detected between either configuration. Controls of extension oligonucleotides alone, with cRGD- conjugated oligonucleotides, with unmodified Nano-winches lacking any ligands, or with Nano-winches decorated with RGE molecules were also performed without significant FAK phosphorylation detected.

### BtuB protein purification and conjugation

Wild-type BtuB was modified with both a cysteine substitution at the third residue in the mature chain (T3C) and a 23-residue N-terminal extension inserted between residues 4–5 consisting of a 6-His tag and Thrombin cleavage site. This extends the N-terminus of BtuB by approximately 8 nm, providing a cysteine to reversibly attach thiolated oligonucleotide, and allowing purification of the protein by affinity chromatography. BtuBT3CHis was expressed from pBAD22 vector in BL21 (DE3) Omp8 cells, to exclude contamination from outer membrane channel proteins, was grown in LB media supplemented with 100 μg/ml ampicillin at 37 °C to OD600 ~0.5 then induced with 0.2% (w/v) arabinose then grown three additional hours. Cells were pelleted and resuspended in buffer A (50 mM Tris-HCl, pH = 7.8, 50 mM NaCl, 5% glycerol), supplemented with 1 mM PMSF, lysed by sonication, cleared by centrifugation at 3,000 × g, 15 min, 4 °C, and membrane collected by centrifugation at 40,000 × g, 30 min, 4 °C. Membranes were resuspended in buffer A and solubilized at 3 mg/ml for 1 hr at room temperature with 1% Triton-X-100. Membranes were pelleted, resuspended in buffer A, and solubilized overnight at 3 mg/ml at 4 °C with 1% LDAO. Membrane was pelleted by centrifugation and supernatant was loaded onto 5 ml HisTrap column (GE Healthcare) equilibrated in buffer A. Protein was eluted using buffer B (50 mM Tris-HCl, pH = 7.8, 300 mM NaCl, 5% glycerol, 600 mM imidazole). Approximately 48 μM BtuBT3CHis was incubated with 80 μM thiol-oligo for 15 min at room temperature with 0.6 mM TCEP. Samples were then incubated with 1.4 mM copper phenanthroline for 15 min, then immediately injected onto a Superdex 200 HR 10/30 column equilibrated in buffer A supplemented with 0.1% LDAO. Fractions were collected and evaluated on 4–20% SDS-PAGE with and without 1 mM TCEP (Supplementary Fig. 45).

### Planar lipid bilayer

Planar bilayers composed of 60 mg/ml azolectin in decane solution were painted across a 0.2-mm aperture in a two compartment chamber containing a symmetrical solution of 1 M KCl, 12 mM MgCl_2_, 1 mM EDTA, 5 mM CaCl_2_, 20 mM HEPES, pH = 7.3 except with 22 mM glycerol in the *cis* compartment and maintained at a constant voltage. Ag-AgCl electrodes were inserted into solutions containing 1 M KCl and were connected to the measurement chamber via agar salt bridges. Data was recorded on Digidata 1440 A with Axoscope and analyzed with ClampFit (version 10.2 Molecular Devices). BtuBT3CHis-oligo was added to the *cis* chamber at ~15 nM. Addition of Nano-winches (97 nt connectors) with complementary anchor strands were added at ~10 nM and allowed to incubate for 20 min before addition of extension oligonucleotides at ~400 nM final concentration. DTT was added to 5 mM to detach the Nano-winch from BtuB.

### Quantification of Nano-winch bound to a MCF-7 cell

To determine the binding level of Nano-winch per cell, the MCF-7 cells were incubated with a fluorescently labeled Nano-winch to provide a quantitative fluorescence intensity distribution for the cells. This was achieved by incorporating an Alexa Fluor 488-labeled DNA staple strand directly into the backstop of the Nano-winch, Supplementary Fig. 33 (5′-end Alexa Fluor-488, IDT Integrated DNA Technologies ALEXA488-AGACAAAAGGGCGACAGGTTTACCAGCGCC-3′). 100 μL MCF-7 cells at 1 × 105 cells/mL suspended in RPMI were mixed for 10 min at 37 °C with fluorescently labeled Nano-winch at saturating concentrations in triplicate, with controls (cells only, fluorescently labeled Nano-winch without cRGD and Nano-winch without Alexa Fluor 488-labeled DNA staple strand). Subsequently, cells were washed twice with 10 mM PBS before it was measured using a flow cytometer. The fluorescence intensity of cells was determined using a BD Biosciences flow cytometer equipped with a 488 nm argon laser and the mean fluorescence intensity (MFI) was calculated, (BD Biosciences version 1.0 software). A sample of unlabelled Nano-winch was used to measure the baseline auto fluorescence in the flow cytometer detectors. To determine the numbers of Nano-winch present per cell, we used a commercially available quantification assay beads QIFIKIT (Agilent Technologies, Germany) which contains five bead populations coated with increasing but defined numbers of surface Alexa-488 fluorophore. QIFIKIT calibration and setup beads were performed according to the manufacturer’s instructions; 100 μL bead suspension was added to 3 mL PBS 0.1% (w/v)-BSA and the resulting mean fluorescence intensity of each population was analyzed. The bead populations (log) MFI was correlated with the (log) number of fluorophore per bead and used to calculate the parameters of linear regression and provide the equation: (log) fluorophore per bead = (slope of line) × (log) sample MFI + (log) fluorophore per bead value when the mean fluorescence intensity is zero. The (log) number of Nano-winch per cell was then calculated from the (log) MFI of the sample using the regression equation.

### Reporting summary

Further information on research design is available in the Nature Research Reporting Summary linked to this article.

## Supplementary information

Supplementary Information

Reporting Summary

## Data Availability

The authors declare that the source data supporting the findings of this study are available within the paper and its Supplementary information files. Source data are provided with this paper.

## Source data

Source Data
