# Supplementary material for: A modular spring-loaded actuator for mechanical activation of membrane proteins
Source: Nat Commun. 2022 Jul 28;13:3182. doi: 10.1038/s41467-022-30745-2 (PMC9334261; doi:10.1038/s41467-022-30745-2)
Supplement: Supplementary file 1 — Supplementary Information [file 41467_2022_30745_MOESM1_ESM.pdf]

Supplementary Materials

A Modular Spring-Loaded Actuator for Mechanical Activation of Membrane Proteins

Mills, A.[1], Aissaoui, N.[1], Maurel, D.[2], Elezgaray, J.[4], Morvan, F.[3], Vasseur, JJ.[3], Margeat, E.[1], Quast, RB.[1], Lai Kee-Him, J.[1] , Saint, N.[5], Benistant, C.[1], Nord, A.[1], Pedaci, F.[1], Bellot, G[1*].

Correspondence to: gaetan.bellot@inserm.fr

This PDF file includes:

Materials and Methods, Supplementary Note 1 to 4, Supplementary Fig. 1 to 47

References 1-15

Materials and Methods

Materials

Oligonucleotides. Desalted staple oligonucleotides were purchased from either Eurofins or Integrated DNA Technologies (IDT). Scaffold p7560 was purified from the M13 bacteriophage replicated in XL1 blue strain *Escherichia coli* (Agilent, USA).

Design and Assembly of DNA origami devices. DNA structures were designed using the honeycomb lattice on caDNAno (v.2.0). Scaffold p7560 at 25nM (Piston-cylinder Origami) or 50nM (Landing Leg Origami) was combined with 200nM of the corresponding staple strands in a buffer of 5mM Tris-HCl, pH 8.0, 1mM EDTA, 18mM $MgCl_2$. The origami were subjected to a thermal annealing ramp: 65°C for 15 minutes, 60°C to 40°C −1°C per every 2 hours, then held at 10°C.

Agarose gel analysis. DNA nanostructures were purified from 1% agarose (0.5X TBE, 45mM Tris-borate, 1mM EDTA, pH 8.3) supplemented with 11mM $MgCl_2$ and 0.5mg·ml^{-1} Sybr SAFE. Samples were migrated on the gel for 3h with a running buffer of 0.5X TBE, 11mM $MgCl_2$ 2.85V·cm^{-1}. Bands corresponding to properly folded DNA origami were excised and transferred to a DNA gel extraction spin column (Merck, France) centrifuged at 5,000 *g* for 5 min at 4°C. Uncropped and unprocessed scans of all the gels are provided in the source data.

Assembly of the Molecular Nano-winch. The full DNA Nano-winch origami was combined with a 2.2-fold molar excess of the Landing Leg origami separately purified from agarose gel in a buffer consisting of 0.5X TBE with 11mM $MgCl_2$. $MgCl_2$ concentration was supplemented up to a final concentration of 20mM and samples were then incubated at 37°C for 10h. Complete nanodevice assembly was evaluated by migration on 1% agarose and bands containing the fully assembled Nano-winch were excised and purified as described above. Incubation of the Nano-winch with cholesterol-conjugated oligonucleotide was performed at 37°C for at least 30 minutes.

Lipid Vesicle Preparation. Small unilamellar vesicles (SUVs) prepared from 1,2-dioleoyl-sn-glycero-3- phosphocholine (DOPC, Avanti Lipids) were solubilized in chloroform and evaporated under a nitrogen stream. Lipid film was then resuspended in a buffer containing 5mM Tris-HCl, pH=7.8, 100mM NaCl, to 1mg/ml final concentration. Lipid suspension was then extruded through 200 nm extrusion membranes (Avanti Lipids, U.S.A). Nano-winches functionalized with cholesterol were allowed to incubate with 0.25mg/ml DOPC SUVs for at least 30 minutes at room temperature before visualization by electron microscopy.

Transmission Electron Microscopy. Purified origami were visualized by adsorption onto glow-discharged carbon-coated grid (Quantifoil Micro tools GmbH, Germany), stained for 60 s with a 2% (w/vol) aqueous uranyl acetate (Merck, France) solution, and then dried with ashless filter paper (VWR, France). Observations of EM grids were carried out on a JEOL 2200FS FEG operating at 200 kV equipped with a 4k × 4k slow-scan CDD camera (Gatan Inc.). Two-dimensional class averages were computed using EMAN2 and ImageJ was used to measure distributions of the different DNA origami nanostructures.

Cyclic RGD-Oligonucleotide and RGE-Oligonucleotide Synthesis. The oligonucleotides were elongated from deoxyguanosine commercially available solid support on an ABI 394 DNA synthesizer according to standard phosphoramidite chemistry protocols (1 µmol scale). Detritylation was performed for 65 s using 3 % TCA in CH_2Cl_2. For the coupling step: benzylmercaptotetrazole (0.3 M in anhydrous CH_3CN) was used as the activator along with commercially available 2'-deoxyribonucleoside-O-2-cyanoethyl, N,N- diisopropylphosphoramidites (0.075 M in CH_3CN, 30 s coupling time) or proparyl- diethyleneglycol phosphoramidite 11 (0.1 M in CH_3CN, 60 s coupling time). The capping step was performed with acetic anhydride using commercially available solutions (Cap A: acetic anhydride:pyridine:THF 10:10:80 v/v/v, and Cap B: 10 % N-methylimidazole in THF) for 10 s. The oxidation step was performed with a standard, diluted iodine solution (0.1 M I2, THF:pyridine:water 90:5:5, v/v/v) for 15 s. Propargyl-oligonucleotide-functionalized CPG beads were introduced into a sealed vial and treated with conc. aq. ammonia (2 mL) overnight at 40 °C. The supernatant was withdrawn and evaporated. The 5'-propargyl modified oligonucleotide 3 was dissolved in water and then analysed by UV, HPLC and MALDI-TOF MS. The amount of 3, determined by UV analysis at 260 nm, was (0.60 µmol, 60 % yield). The HPLC purity at 260 nm was 79 %. Analytical RP- HPLC retention time: 11.86 min on Macherey Nagel Nucleodur 100−3 C 18 ec column (length: 75 mm, ID: 4.6 mm) with a linear gradient of 1 to 25% of CH3CN in TEAAc 0.05 M pH7. MALDI-TOF MS: m/z: [M-H]−: for C217H269N87O130P21 calcd.: 6826.48; found: 6826.93. CuAAC conjugation was performed on crude of 3. To 600 nmol of 3 in 240 µL of water was added the RGD azide 42 1200 nmol or RGE 42 4300 nmol (240 µL of a 5 mM solution in methanol, 2eq) 240 µL of 2M TEAAc and ~0.1 mg of Cu0 nanopowder. The mixture was sonicated for 60 sec and then heated under microwaves assistance at 65°C for 1h. After centrifugation, the supernatant was withdrawn and saturated EDTA solution was added (500 µL). After 5 min, the solution was desalted by size exclusion chromatography (Nap10). The resulting solution was treated for 1h with quadrapure IDA resin. The supernatant was then evaporated. The crude was purified by HPLC

on a Macherey Nagel Nucleodur C18 HTec column (length: 250 mm, ID: 10 mm), using a linear gradient from 4 to 17 % of CH_3CN in 50 mM TEAAc pH 7 for 20 min, affording 200 nmol of 5 (33 % yield for RGD and 36 % yield for RGE). Analytical RP-HPLC retention time for RGD: 13.49 min. MALDI-TOF MS: m/z: [M-H]−: for C244H308N98O137P21 calcd.: 7455.15; found: 7456.99.

Integrin Activation by the Nano-winch. Phospho-FAK (Tyr397) cellular assay: phospho-FAK assay is based on an Homogeneous Time- Resolved FRET (HTRF®) sandwich immunoassay format comprising two specific monoclonal anti-FAK antibodies, one labeled with Europium- cryptate (donor) and the other labeled with d2 (acceptor). Upon integrin pathway activation, FAK is phosphorylated on Tyr397 leading to a close proximity between the dyes. In this configuration, the excitation of the donor with a light source triggers a Luminescent Resonance Energy Transfer (LRET) towards the acceptor, which in turn fluoresces at a specific wavelength (665nm). The specific signal modulates positively in proportion to phospho-FAK (Tyr397). Phospho-FAK (Y397) Monoclonal Antibody was produced by PerkinElmer (Catalog # MAB4528), PerkinElmer source : Monoclonal Rat IgG2A Clone # 820755. Antibody has been validated by PerkinElmer by demonstrating immunoblotting on Human MCF-7 and HeLa cells. FAK reagents were purchased at CisBio bioassays (Marcoules, France).

Cell culture. MCF-7 cells (Michigan Cancer Foundation – 7) were grown in RPMI supplemented with 10% FBS (without antibiotics) at 37°C, 5% CO_2. Cells were split twice a week in 75 cm^2 flasks. It must be specified that MCF-7 cells grow in clusters and are therefore difficult to dissociate with Versene, making the calculation of the number of cells inaccurate. MCF-7 cells were starved for twenty-four hours in RPMI before performing the experiment. The day of the assay, cells were detached with 5ml Versene 1X solution (Gibco) for 10-15 minutes at 37°C, 5% CO_2. After adding 5ml RPMI, cells were centrifuged for five minutes at 300 x g. The cell pellet was resuspended in RPMI in order to have the required number of cells (optimal was 10,000 – 20,000 cells, Supplementary Fig. S28) in 4µl of medium. 4µl of cells (10,000 – 40,000 cells/well) were plated in a 96-well white plate low volume (CisBio bioassays, Marcoules, France) and mixed with 4µl of experimental and control DNA constructs stored in Folding Buffer 1X (5mM Tris-HCl pH7.8, 1mM EDTA, 18mM $MgCl_2$). FB1X was supplemented with 100mM NaCl and 20mM $MgCl_2$ extemporaneously. Cells were then lysed by adding 4µl of supplemented lysis buffer 4X and incubated for at least 30 minutes at room temperature under shaking. It was found that between five to 30 minutes after incubation of cells with cRGD-Nano-winches was sufficient to observe FAK phosphorylation (Supplementary Fig. 31). Finally, 4µl of premixed antibody solution (vol/vol) prepared in the detection buffer were added to lysed cells and incubated 2 hours at room temperature, phospho-FAK

(Tyr397) kit from PerkinElmer reference : 64FAKPEH. HTRF readings were collected using a PHERAstar plate reader (BMG Labtech) at two specific wavelengths: 665 nm for the acceptor (A) and 620 nm for the donor (D). Phospho-FAK signal was assessed by calculating the ratio R(IA/ID)-R0. R0 is the background signal from the antibodies alone that was removed to the signal measured on lysed cells. The effect of Nano-winches was tested on MCF-7 cells in both autonomous and remote configurations. Nano-winches were incubated with MCF-7 cells in suspension prior to addition of 30µM of extension oligonucleotides. No significant difference in FAK phosphorylation was detected between either configuration. Controls of extension oligonucleotides alone, with cRGD- conjugated oligonucleotides, with unmodified Nano-winches lacking any ligands, or with Nano-winches decorated with RGE molecules were also performed without significant FAK phosphorylation detected.

BtuB Protein Purification and Conjugation. Wild-type BtuB was modified with both a cysteine substitution at the third residue in the mature chain (T3C) and a 23-residue N-terminal extension inserted between residues 4 to 5 consisting of a 6-His tag and Thrombin cleavage site. This extends the N-terminus of BtuB by approximately 8nm, providing a cysteine to reversibly attach thiolated oligonucleotide, and allowing purification of the protein by affinity chromatography. $BtuB_{T3C}His$ was expressed from pBAD22 vector in BL21 (DE3) Omp8 cells, to exclude contamination from outer membrane channel proteins, was grown in LB media supplemented with 100µg/ml ampicillin at 37°C to OD_{600} ~0.5 then induced with 0.2% (w/v) arabinose then grown three additional hours. Cells were pelleted and resuspended in buffer A (50mM Tris-HCl, pH=7.8, 50mM NaCl, 5% glycerol), supplemented with 1mM PMSF, lysed by sonication, cleared by centrifugation at 3,000xg, 15 minutes, 4°C, and membrane collected by centrifugation at 40,000xg, 30 minutes, 4°C. Membranes were resuspended in buffer A and solubilized at 3mg/ml for 1hr at room temperature with 1% Triton-X-100. Membranes were pelleted, resuspended in buffer A, and solubilized overnight at 3mg/ml at 4°C with 1% LDAO. Membrane was pelleted by centrifugation and supernatant was loaded onto 5ml HisTrap column (GE Healthcare) equilibrated in buffer A. Protein was eluted using buffer B (50mM Tris-HCl, pH=7.8, 300mM NaCl, 5% glycerol, 600mM imidazole). Approximately 48µM $BtuB_{T3C}His$ was incubated with 80µM thiol-oligo for 15 minutes at room temperature with 0.6mM TCEP. Samples were then incubated with 1.4mM copper phenanthroline for 15 minutes, then immediately injected onto a Superdex 200 HR 10/30 column equilibrated in buffer A supplemented with 0.1% LDAO. Fractions were collected and evaluated on 4-20% SDS-PAGE with and without 1mM TCEP (Supplementary Fig. 44). Uncropped and unprocessed scans of all the gels are provided in the source data.

Planar Lipid Bilayer. Planar bilayers composed of 60mg/ml azolectin in decane solution were painted across a 0.2-mm aperture in a two compartment chamber containing a symmetrical solution of 1M KCl, 12mM $MgCl_2$, 1mM EDTA, 5mM $CaCl_2$,

20mM HEPES, pH = 7.3 except with 22mM glycerol in the *cis* compartment and maintained at a constant voltage. Ag-AgCl electrodes were inserted into solutions containing 1M KCl and were connected to the measurement chamber via agar salt bridges. Data was recorded on Digidata 1440A with Axoscope and analyzed with ClampFit (version 10.2 Molecular Devices). $BtuB_{T3C}$His-oligo was added to the *cis* chamber at ~15nM. Addition of Nano-winches (97nt connectors) with complementary anchor strands were added at ~10nM and allowed to incubate for 20 minutes before addition of extension oligonucleotides at ~400nM final concentration. DTT was added to 5mM to detach the Nano-winch from BtuB.

Flow cytometry. To determine the binding level of Nano-winch per cell, the MCF-7 cells were incubated with a fluorescently labeled Nano-winch to perform flow cytometry analysis and to provide a quantitative fluorescence intensity distribution for the cells. This was achieved by incorporating an Alexa Fluor 488-labeled DNA staple strand directly into the backstop of the Nano-winch, Supplementary Fig. 32 (5'-end Alexa Fluor-488, IDT Integrated DNA Technologies ALEXA488-AGACAAAAGGGCGACAGGTTTACCAGCGCC-3'). 100 µL MCF-7 cells at 1 × 105 cells/mL suspended in RPMI were mixed for 10 minutes at 37°C with fluorescently labeled Nano-winch at saturating concentrations in triplicate, with controls (cells only, fluorescently labeled Nano-winch without cRGD and Nano-winch without Alexa Fluor 488-labeled DNA staple strand). Subsequently, cells were washed twice with 10 mM PBS before it was measured using a flow cytometer. The fluorescence intensity of cells was determined using flow cytometry (BD Biosciences flow cytometer equipped with a 488 nm argon laser and BD Biosciences version 1.0 software), and the mean fluorescence intensity (MFI) was calculated. A sample of unlabelled Nano-winch was used to measure the baseline auto fluorescence in the flow cytometer detectors. To determine the numbers of Nano-winch present per cell, we used a commercially available quantification assay beads QIFIKIT (Agilent Technologies, Germany) which contains five bead populations coated with increasing but defined numbers of surface Alexa-488 fluorophore. QIFIKIT calibration and setup beads were performed according to the manufacturer's instructions; 100 µL bead suspension was added to 3 mL PBS 0.1% (w/v)-BSA and the resulting mean fluorescence intensity of each population was analyzed. The bead populations (log) MFI was correlated with the (log) number of fluorophore per bead and used to calculate the parameters of linear regression and provide the equation: (log) fluorophore per bead = (slope of line) × (log) sample MFI + (log) fluorophore per bead value when the mean fluorescence intensity is zero. The (log) number of Nano-winch per cell was then calculated from the (log) MFI of the sample using the regression equation.

Supplementary Notes

Supplementary Note 1: Mechanical model described by the Worm Like Chain model

To estimate the forces at play in the nano-winch, we consider the simplified structure of Supplementary Fig. 23. The piston, free to slide, is attached to *n* number of ssDNA molecules at the top and *n* at the bottom, both considered as springs with non-linear stiffness $k_{DNA1}(x)$ and $k_{DNA2}(x)$, respectively, a function of the extension of the corresponding molecule. The tip of the piston is also anchored to a membrane protein, considered for simplicity as a spring of constant stiffness k_{pr}. We further assume that the ssDNA molecules have a resting position at zero extension, therefore apply force only when stretched but not when compressed (considering instead non-zero resting positions would lower the force estimated in the following). In equilibrium, $x = x_{eq}$ and the force balance is given by

$$nk_{DNA}(H - x_{eq})(H - x_{eq}) = k_{pr}(x_{eq}) + k_{DNA}(x_{eq})(x_{eq}) \quad [1]$$

Where H = 30 nm is the maximum possible displacement of the piston, and n is the number of ssDNA molecules involved, and we have used the small angle approximation, H-x ~ L and $k_{DNA1} = k_{DNA2} = k_{DNA}$ as we consider both molecules with the same length, for design 97nt only.

For ssDNA, the relationship between the stretching force F and the molecular extension x can be described by the Worm Like Chain model *(*1*)* as

$$x(F) = L_{ss}\left(\coth(\frac{Fb}{K_BT}) - \frac{K_BT}{Fb}\right)(1 + \frac{F}{S}) \quad [2]$$

where L_{SS} = 61 nm is the contour length (L_{SS} = nt x d_{nt}, where nt = 97 is the number of nucleotides, d_{nt} = 0.67 nm is the distance between bases for ssDNA *(*2*)*, b = 1.5 nm is the Kuhn length, and S = 800 pN is stretch modulus *(*1*)*.

For a given value of k_{pr}, the equilibrium position, solution of eq.[1], can be visualized by the intersection of the total force exerted by the two ssDNA and by the linear

spring k_{pr} as functions of x (Supplementary Fig. 23). For protein, a typical value for k_{pr} range from ~ 0.002 N/m to 0.02 N/m, *(*3,4*)*. For n = 6, choosing k_{pr} of 0.002 or 0.02 N/m, we find the force applied by the structure on the surface is 15 and 30 pN respectively.

Supplementary Note 2: Mechanical model based on coarse-grained Monte Carlo simulations.

Treating the DNA like a WLC spring may be quantitatively not exact in our case, because it applies to small bending but it gives a first approximation. It has been shown (5) that for short (~100nt) double stranded DNA segments, the persistence length can be half of that observed for longer double strands, due to the importance of 'kinks'. To better explore the mechanical behaviour, we performed coarse-grained molecular dynamics (brownian dynamics) simulations of short single and double strands, with respectively 30, 60 and 97 nucleotides, using the oxDNA simulator. oxDNA is a simulation code developed to implement Monte Carlo and Molecular Dynamics and provide a physical representation of the thermodynamic and mechanical properties of single- and double-stranded DNA, as well as the transition between them *(*6*)*. Computations were done with T = 23C and folding buffer salt conditions. For each of these systems, a series of Monte Carlo trajectories with constrained end to end distance was performed, which yields the force to distance relation F(x). At equilibrium, projections along the vertical axis (see Supplementary Fig. 23) yield the expression:

$$F_{up} = F_{down}(L(x)) \sin(\alpha(x)) \quad [3]$$

The left and right sides of the equation correspond to the forces applied on the upper and lower cylinder-legs blocks, respectively, and x is the distance between the piston and the surface. In this expression, L is the length of the linker between cylinder and base of the piston, and H, w, and alpha are as labeled in Supplementary Fig. 24:

$$L(x) = \sqrt{w^2 + (H-x)^2} \qquad sin(\alpha(x)) = \frac{H-x}{L(x)} \quad [4]$$

To estimate the force exerted by the nanomachine upon a biomolecule, such as a membrane protein, we modelled the latter as an additional linear spring with stiffness k_{pr}. The data are reported in Supplementary Fig. 24, for single and double stranded connectors, and, as a comparison, the force computed with K_{pr} from 0.0002 N/m to 0.02 N/m.

In the limit of small deformations dx, the force exerted by the Nano-winch can be approximated by k_{winch} dx where k_{winch} is the effective stiffness. We found the following values :

Stiffness (N/m)	30 nucleotides	60 nucleotides	97 nucleotides
ssDNA connector	0.02	0.006	0.003
dsDNA connector	0.68	0.05	0.008

As an element of comparison, commercially available soft AFM cantilevers are characterized by stiffness around 0.05 N/m.

Supplementary Note 3: Model of Nano-winch position:

The six parallel connector strands which connect the backstop to the cylinder become stiffer upon annealing of the complementary oligonucleotides, pushing the piston upwards. Double-stranded DNA behaves as a semiflexible polymer, which can be described with worm-like chain model *(7)*. The thermal conformation of the polymer chain can be characterized by the radial distribution function *G(r; L)* of the end-to-end distance *r* for a given contour length *L* and persistence length l_p (150 bp for dsDNA). We have used the calculated distribution given by Wilhem and Frey *(7)*,

$$G(r) = \frac{2\kappa}{4\pi N} \sum_{k=1}^{\inf} \pi^2 k^2 (-1)^{(k+1)} e^{-\kappa\pi^2 k^2 (1-r)} \quad (1)$$

where $\kappa = l_p/L$ and *N* is a normalization factor, an explicit expression for which is given by Becker *et al.*, :

$$\begin{aligned} N =& \frac{-75e^{5/4}\pi^5\kappa^3}{N'}; \\ N' =& 400e^{5/4}\pi^{13/2}\mathrm{erfc}\left(\frac{\sqrt{5}}{2}\right)(6\kappa+1) + 9000\sqrt{5}e^{\pi^2(1/5-\kappa)} \\ & + 12\sqrt{5}[-15\pi^4(1-5\kappa)^2 + 150\pi^2(5\kappa-1) \\ & - 4\pi^6(5\kappa(5\kappa+2)+3) - 750]. \end{aligned} \quad (2)$$

G(r) is shown in Supplementary Fig. 37 for one dsDNA strand and multiple strands in parallel. The average

end-to-end distance of j DNA molecules in parallel *(8,9)* is,

$$\langle l_{wlc} \rangle = \int_0^{\inf} r[G(r,L)4\pi r^2]^j dr. \quad (3)$$

Supplementary Note 4: Model of membrane deformability:

We have derived an analytical expression for the load force F as a function of the indentation depth h, to better evaluate how these mechanical properties could contribute to the overall elastic membrane response. We followed the theoretical considerations described in A. Janshoff et al. paper (10)*. To evaluate how the bending mechanical properties contribute to the overall elastic membrane response, an analytical expression for the load force F as a function of indentation has been previously derived* (11)*. It is apparent from their force–indentation curves that the elastic response of the membrane is linear, so adding a mechanical stress through a point load like the tip of the Nano-winch piston to the membrane will yield* (12, 13, 14) *with the load force F in the indentation h, and g(v) being a numerical solution for all possible Poisson's ratios v (*14*)*

$$F = \frac{4\pi E t^3}{3(1-\nu^2)R^2_{\text{Nano-Winch}}} h = \frac{64\pi\kappa}{R^2_{\text{Nano-Winch}}} h \tag{1}$$

As the mechanical response of the membrane is linear as expected from Eq. (1) an apparent spring constant k_{app} can be defined :

$$k_{\text{app}} = \left(\frac{\partial F}{\partial h}\right)_{\rho=0} \tag{2}$$

To evaluate the bending contribution to the mechanical response of the membrane upon indentation, k_{app} for pure bending can be calculated according to :

$$F \cong \sigma_0 \pi h + g(\nu)\frac{Et}{R^2_{\text{Nano-Winch}}} h^3 \tag{3}$$

with a typical value for the bending stiffness of $\kappa = 10^{-19}$ J and a radius of R = 100 nm, k_{app} = 9.3 pN m^{-1}.

Because the force is linear with displacement, if the machine applies 1.0 pN, then the membrane will be vertically stretched by a displacement estimated to be ~1.0 Å.

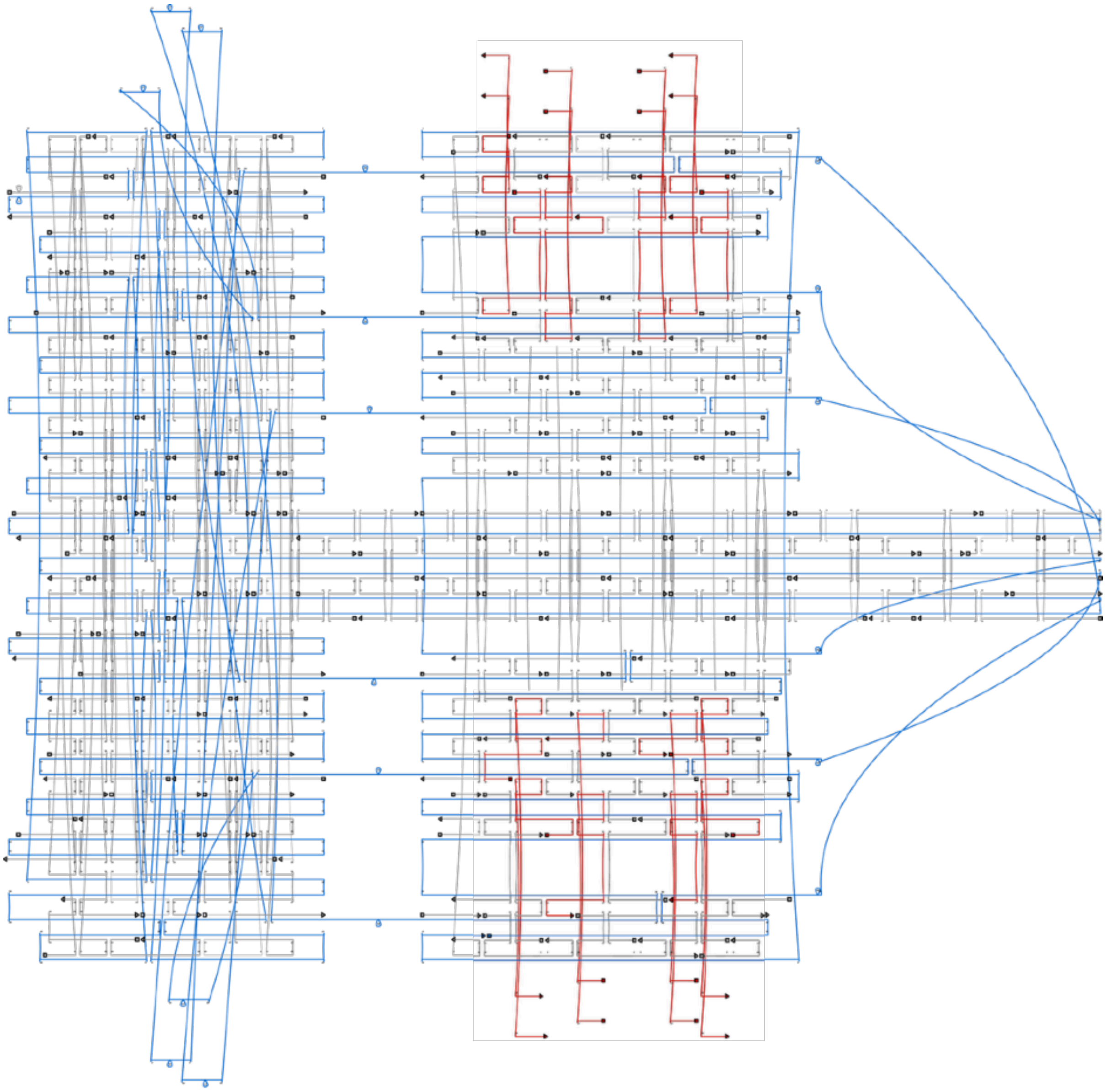

Supplementary Fig. 1. caDNAno design diagram of the Piston-cylinder. The single-stranded scaffold is shown in blue and staples in grey. Sequences to connect Legs-origami are shown in red.

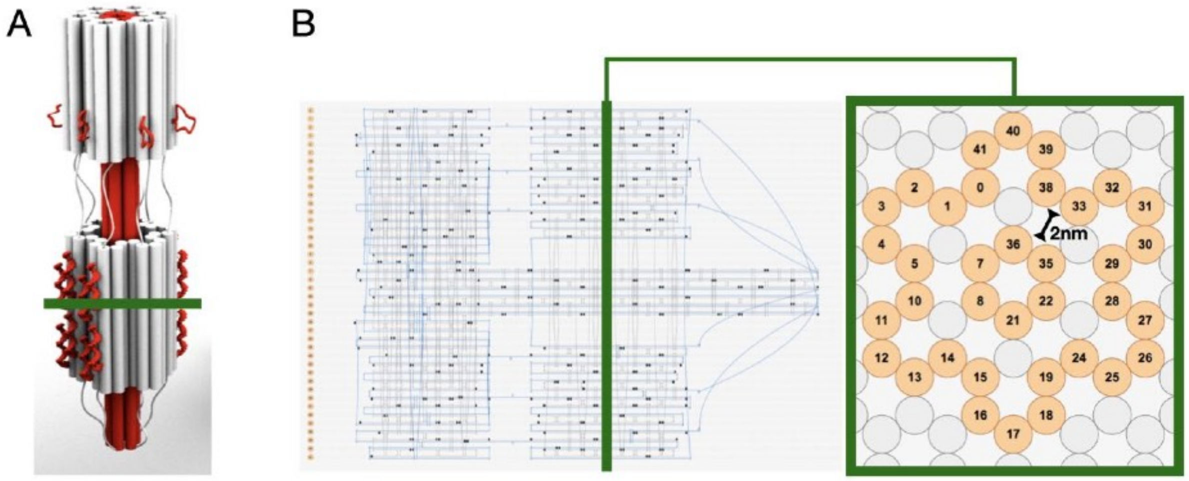

Supplementary Fig. 2. Design of the piston-cylinder. (A) Schematic illustration of the piston-cylinder. (B) Piston-cylinder Cadnano design, the design consists of two domains, a long six-helix bundle piston at the center topped with a backstop (left side on the cadnano design), and a cylinder folded concentrically around the piston with a ~2nm gap between them, cross section right side on the figure.

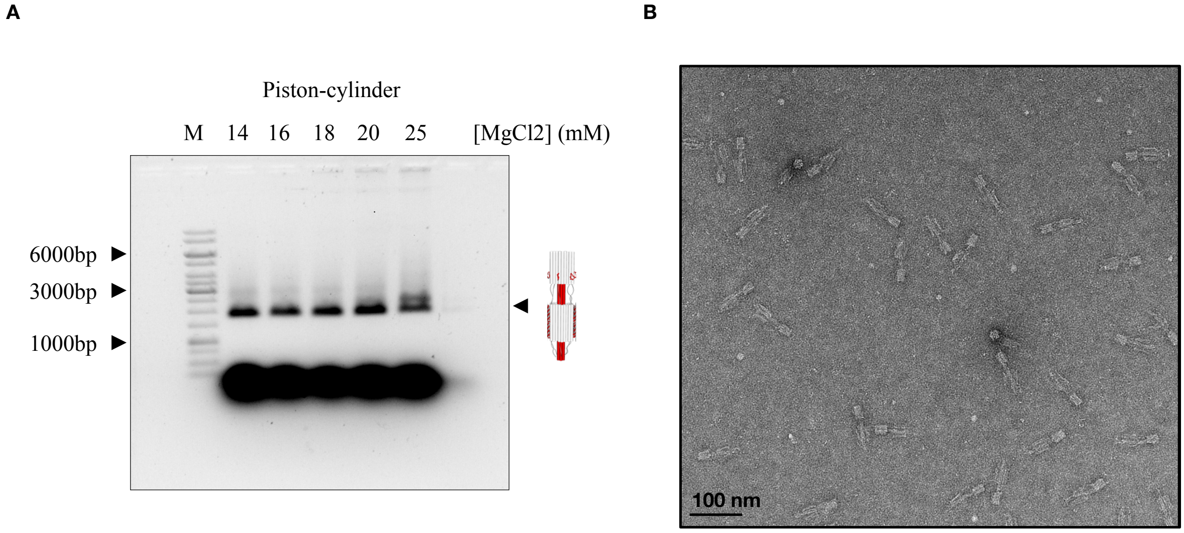

Supplementary Fig. 3. Piston-cylinder design and folding results. A) The optimal folding conditions for the Piston-cylinder were evaluated by performing the folding in different concentrations of magnesium. The resulting origami were separated on a 1% agarose gel with 11mM $MgCl_2$ within the gel and the 0.5X TBE buffer. B) Exemplar TEM image of 97-nt connector length Piston- cylinder origami folded in 18mM $MgCl_2$ after purification from agarose gel. Uncropped and

unprocessed scans of all the gels and unprocessed TEM image are provided in the source data.

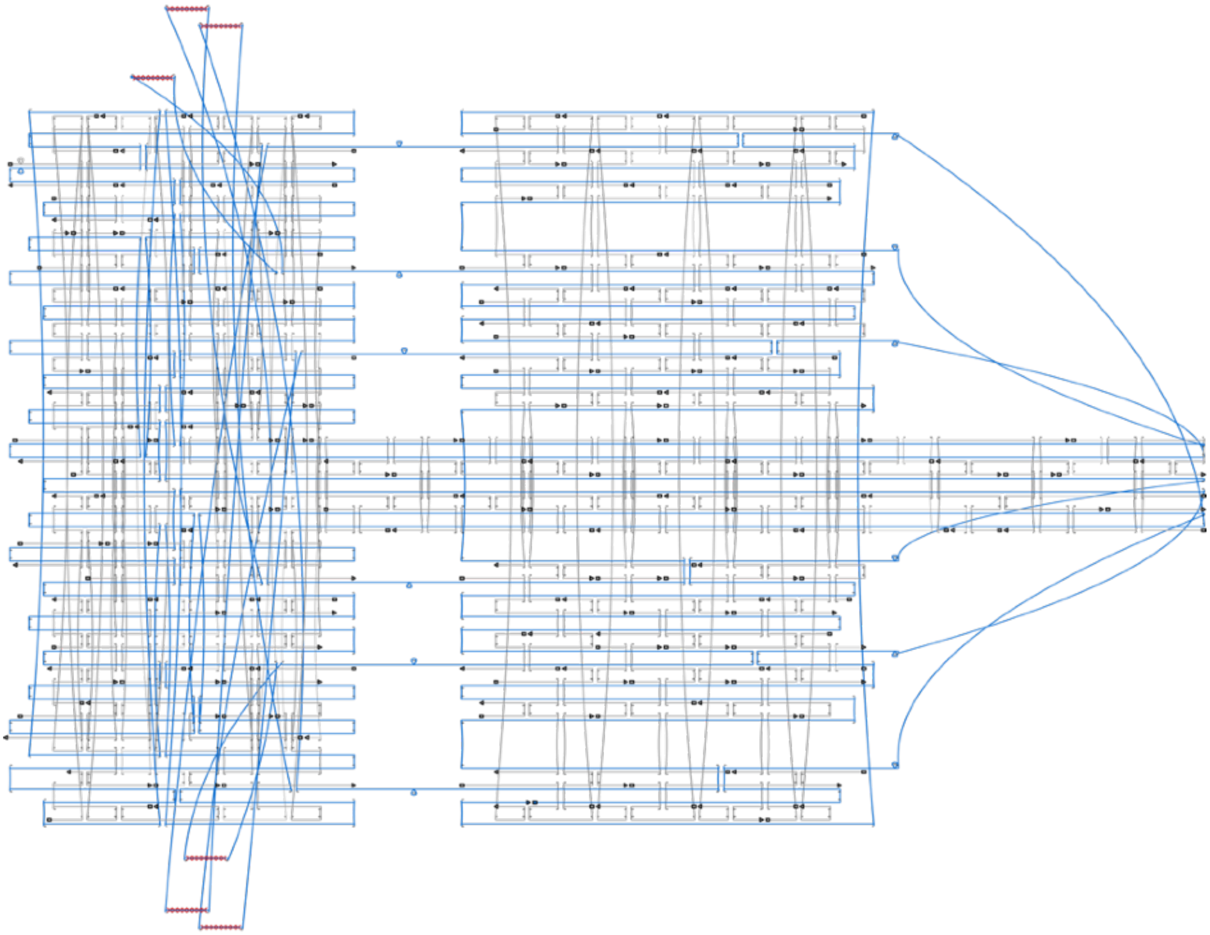

Supplementary Fig. 4. caDNAno design diagram of the 97-nt Connector Piston-cylinder. The single- stranded scaffold is shown in blue and staples in grey.

97-nt Connector Piston-Cylinder

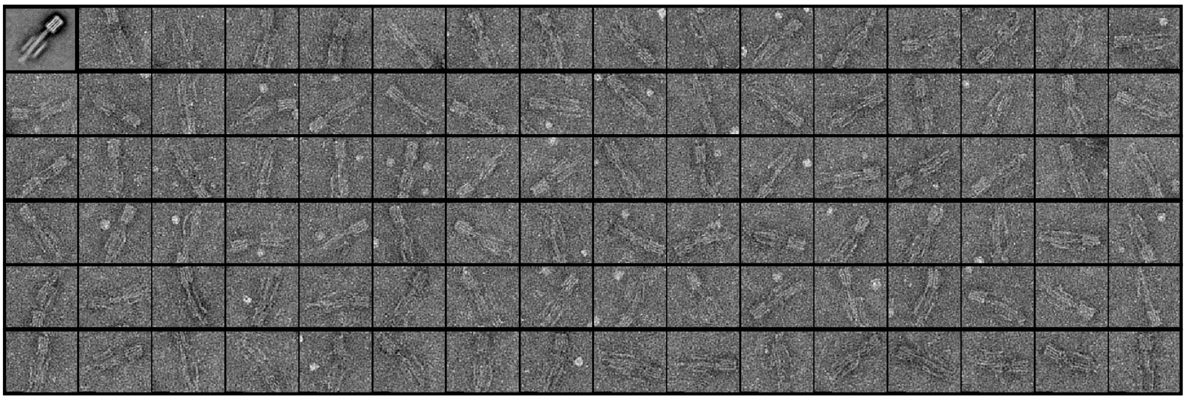

Supplementary Fig. 5. 97-nt Connector Piston-cylinder Particle Set. Representative set of particles of individual 97-nt connector piston-cylinder origami from TEM micrographs. The top left square is an average image. Each square is 92nm x 92nm.

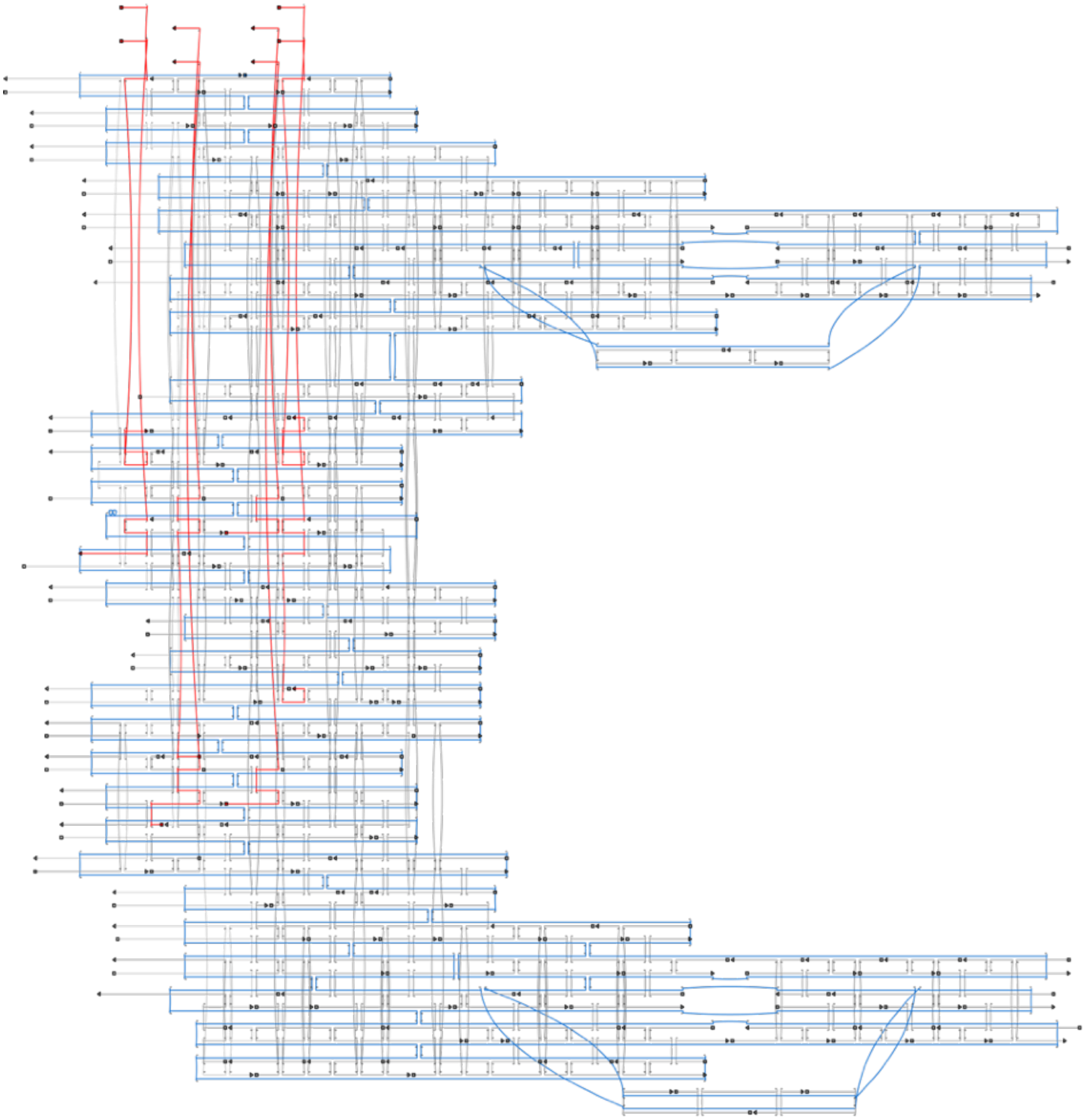

Supplementary Fig. 6. caDNAno design diagram of the Landing Leg-origami. The single-stranded scaffold is shown in blue and staples in grey. Sequences to connect Piston-cylinder origami are shown in red.

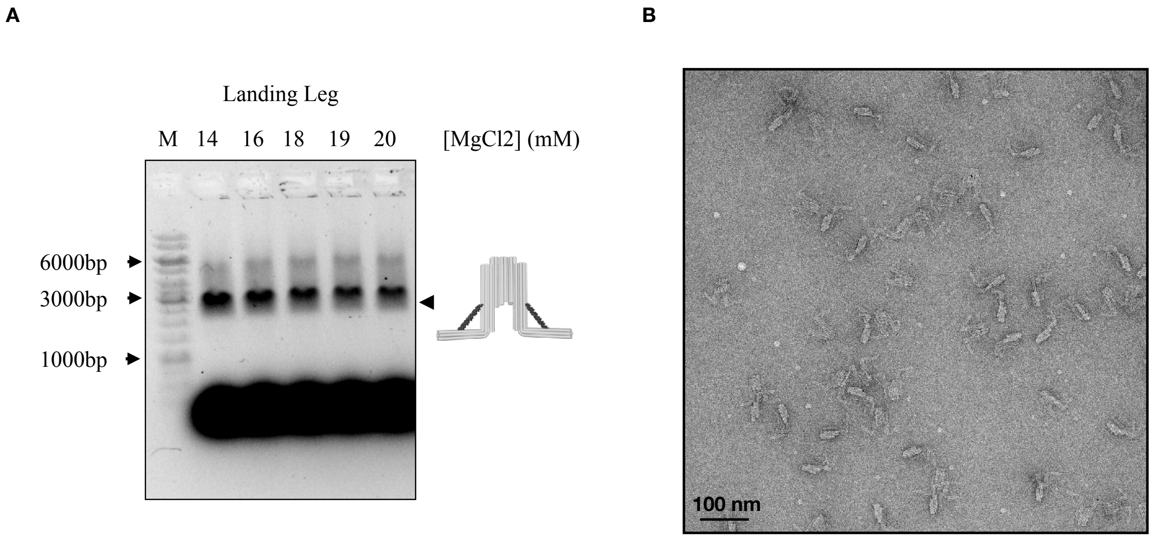

Supplementary Fig. 7. Landing Leg Origami. A) The optimal folding conditions for the Landing Leg performed using a gradient of different magnesium concentrations. Origami were separated from excess staple oligonucleotides and evaluated in 1% agarose with 0.5XTBE and 11mM $MgCl_2$. Uncropped and unprocessed scans of all the gels are provided in the source data. **B)** Exemplar TEM image of Landing Leg origami folded in 18mM $MgCl_2$ after purification from agarose gel.

Landing Leg Side View

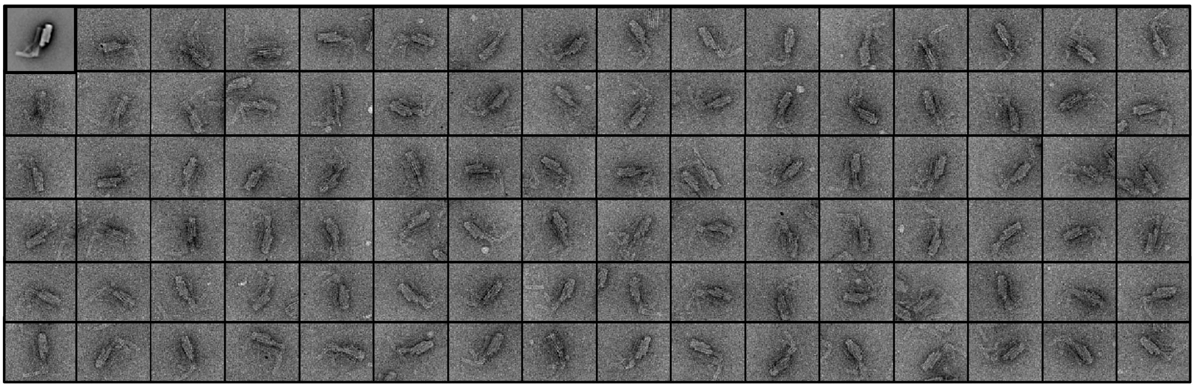

Supplementary Fig. 8. Landing Leg Front View Particle Set. Representative set of individual Landing Leg origami particles from TEM micrographs and only from the front view of the origami. The top left square is an average image. Each square is 92nm x 92nm.

Landing Leg Front and Back View

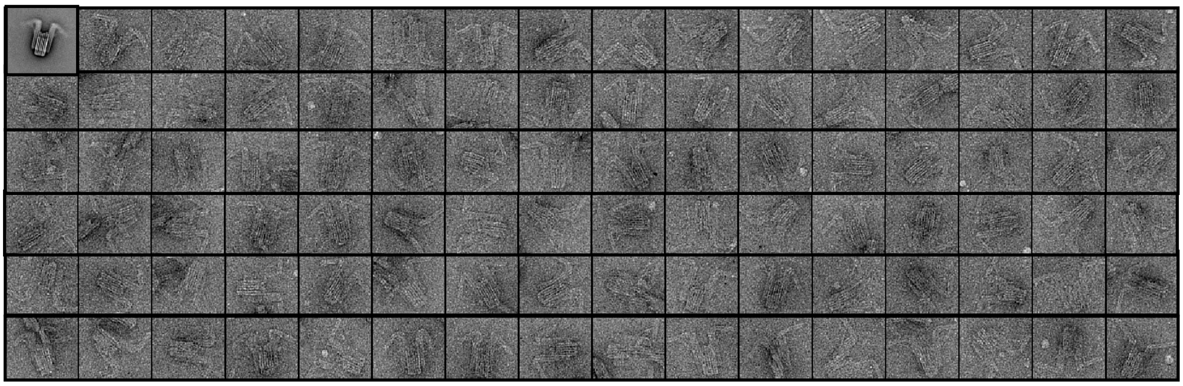

Supplementary Fig. 9. Landing Leg Side View Particle Set. Representative set of individual Landing Leg origami particles from TEM micrographs and only from the lateral or side view of the origami. The top left square is an average image. Each square is 92nm x 92nm.

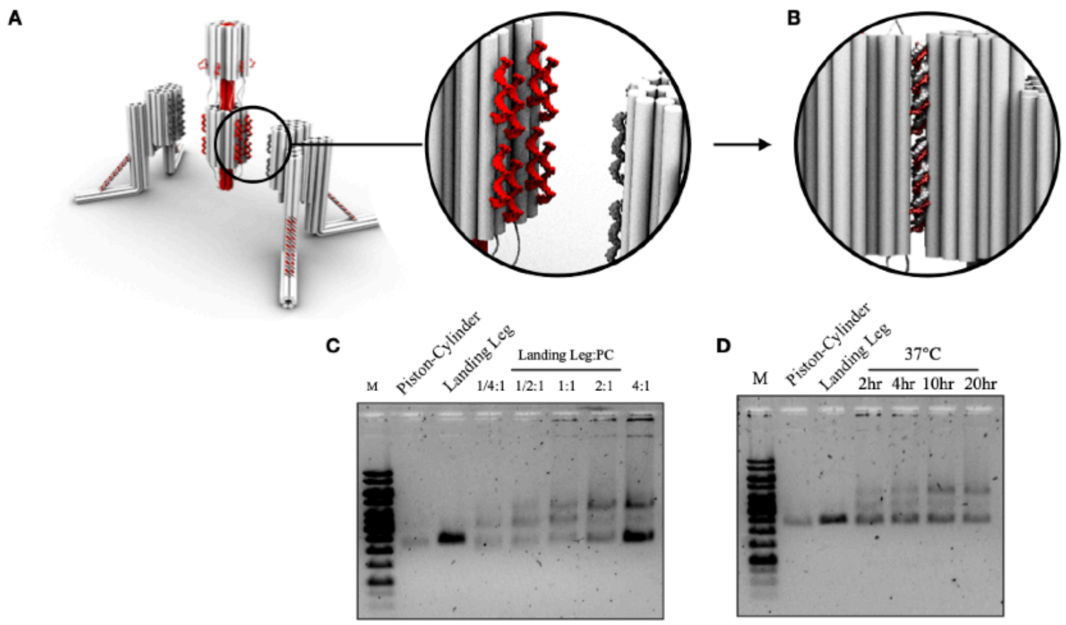

Supplementary Fig. 10. Nano-winch Assembly. A) Complementary strands on the Piston-Cylinder (red) and the Landing Leg (grey) anneal together to **B)** adhere the Landing Leg to the sides of the cylinder domain. **C)** Nano-winches assembly were tested using different molar ratios of Landing Leg to Piston-cylinder (PC) and analyzed on 1% agarose gel in 0.5XTBE buffer and 11mM $MgCl_2$. A ratio of 2:1 Landing Leg:Piston-cylinder was sufficient to achieve the full ternary complex. **D)** Nano-winch assembly was tested for different incubation periods at 37°C then analyzed on 1% agarose gel in 0.5XTBE buffer and 11mM $MgCl_2$. Uncropped and unprocessed scans of all the gels are provided in the source data.

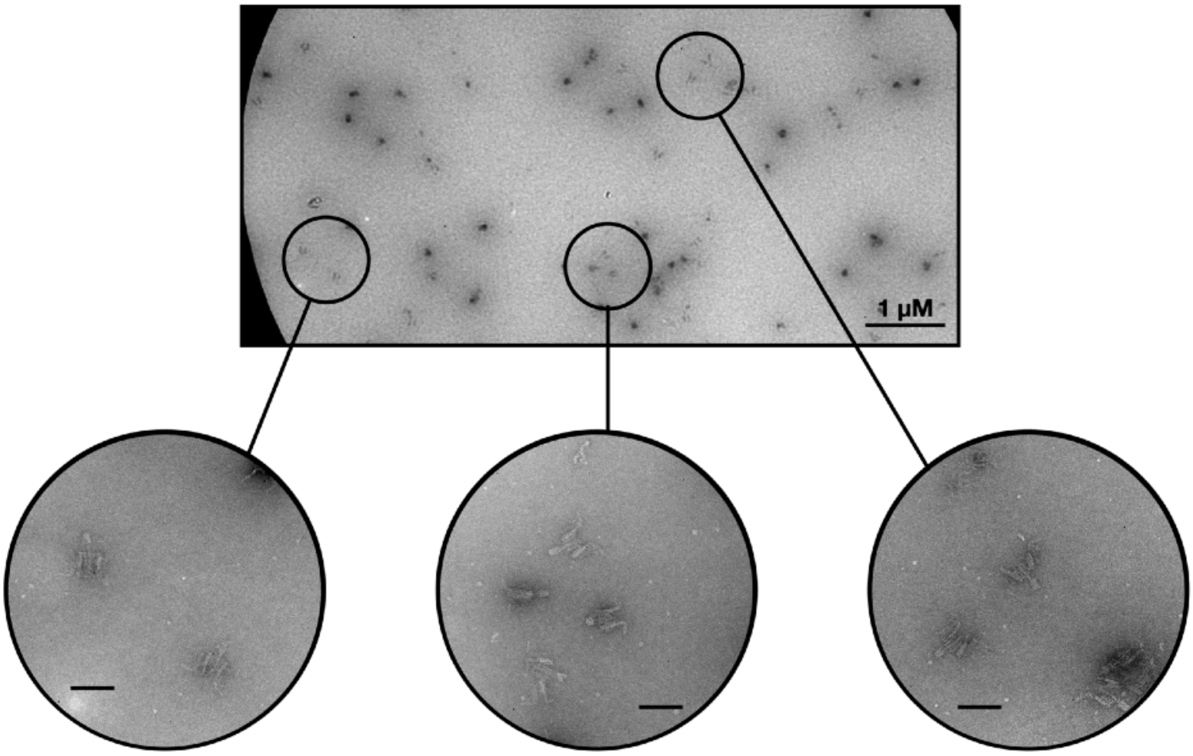

Supplementary Fig. 11. Assembly of the Nano-winch. Nano-winch assembly was checked using negative- stain TEM with individual particles in detail. Black bars represent 50nm and unprocessed TEM image are provided in the source data.

.

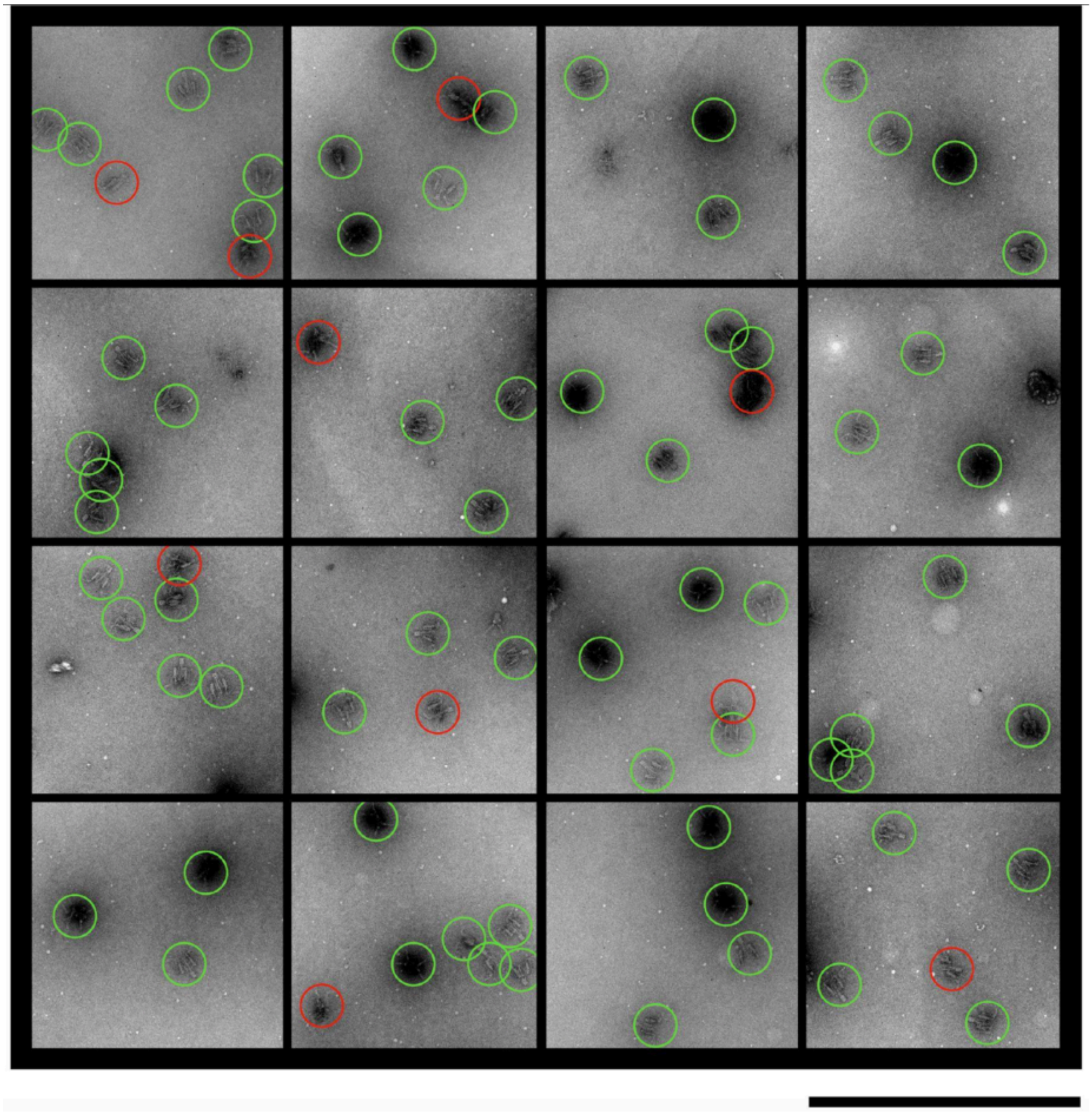

Figure. S12: Nano-winch Assembly Validation. Correct assembly of the Nano-winch was evaluated using NS-TEM. Green circles correspond to correctly assembled nanostructures, red circles indicate incorrect assembly or damaged Nano-winches. Folding of the Nano-winch in high yield (*87*%) in their trimeric forms was estimated from a total number of templates, N= 78. Black bar represents 1μm.

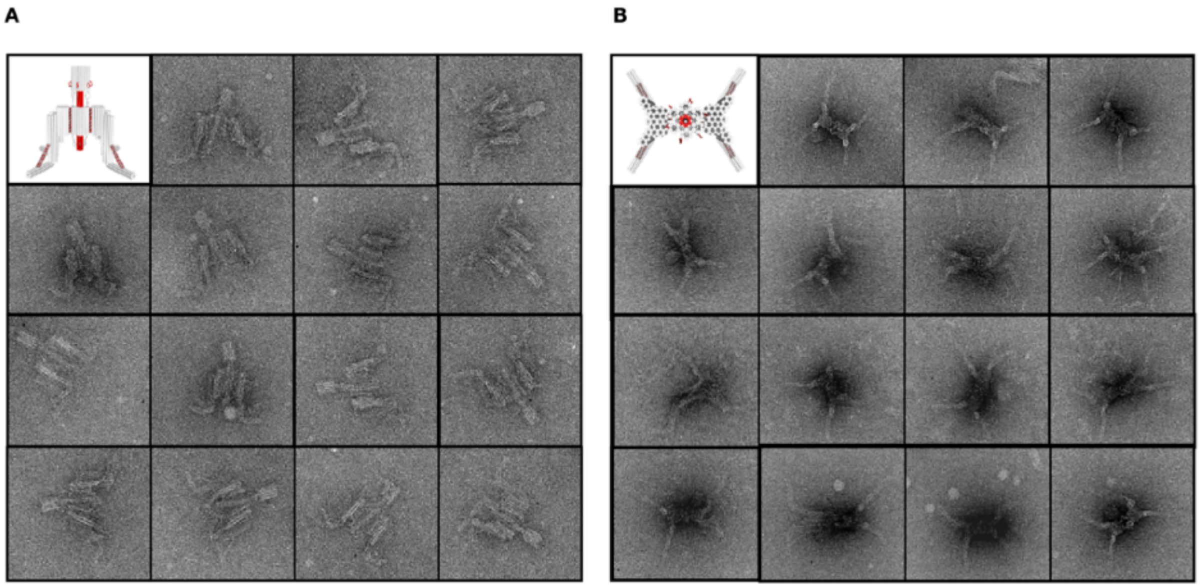

Supplementary Fig. 13. Nano-winch. Exemplar images of the assembly of the the full Nano-winch with **A)** side view, and **B)** top view.

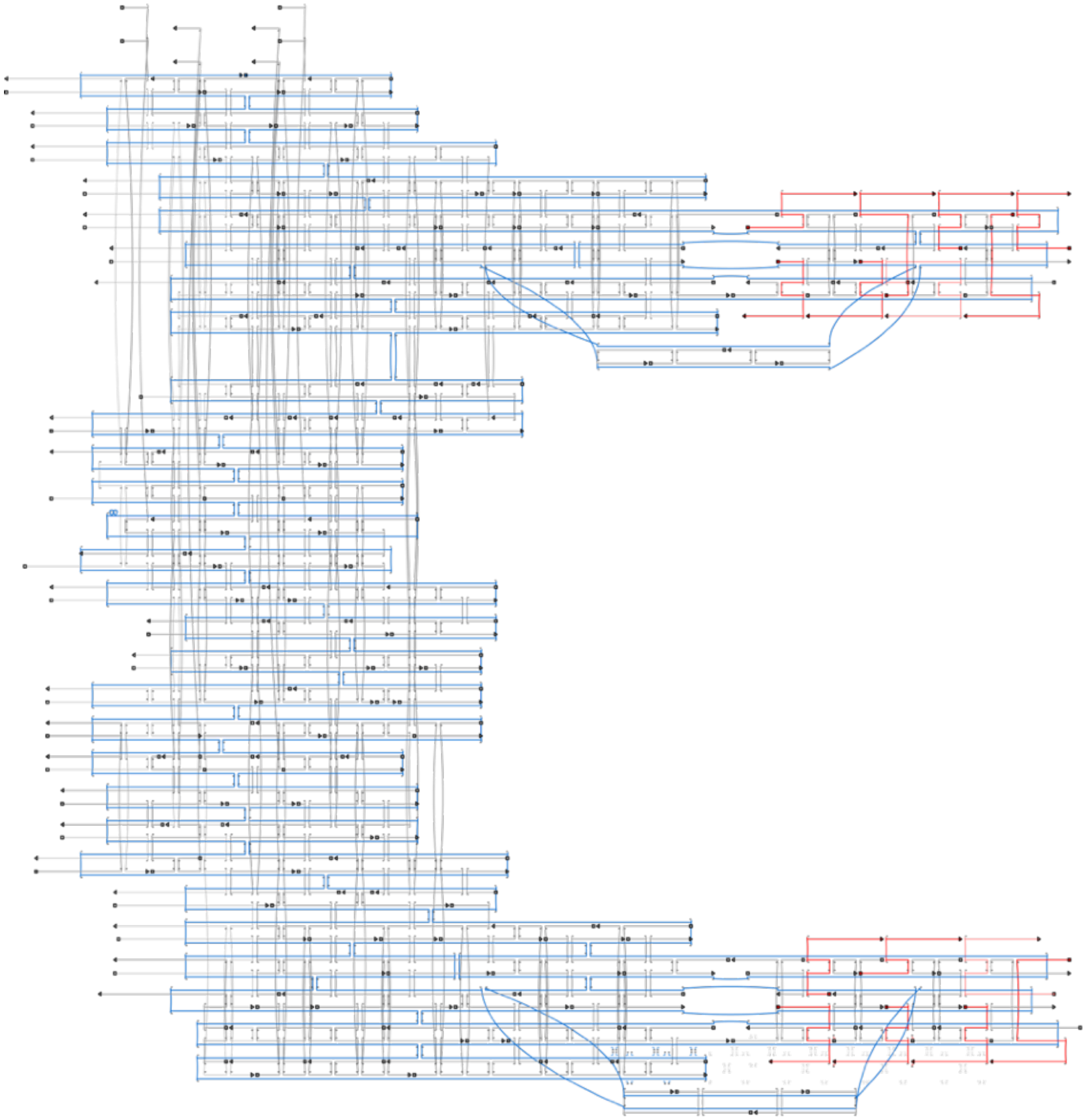

Supplementary Fig. 14. caDNAno design diagram of the Leg-origami cholesterol. The single-stranded scaffold is shown in blue and staples in grey. Sequences for cholesterol functionalization are shown in red.

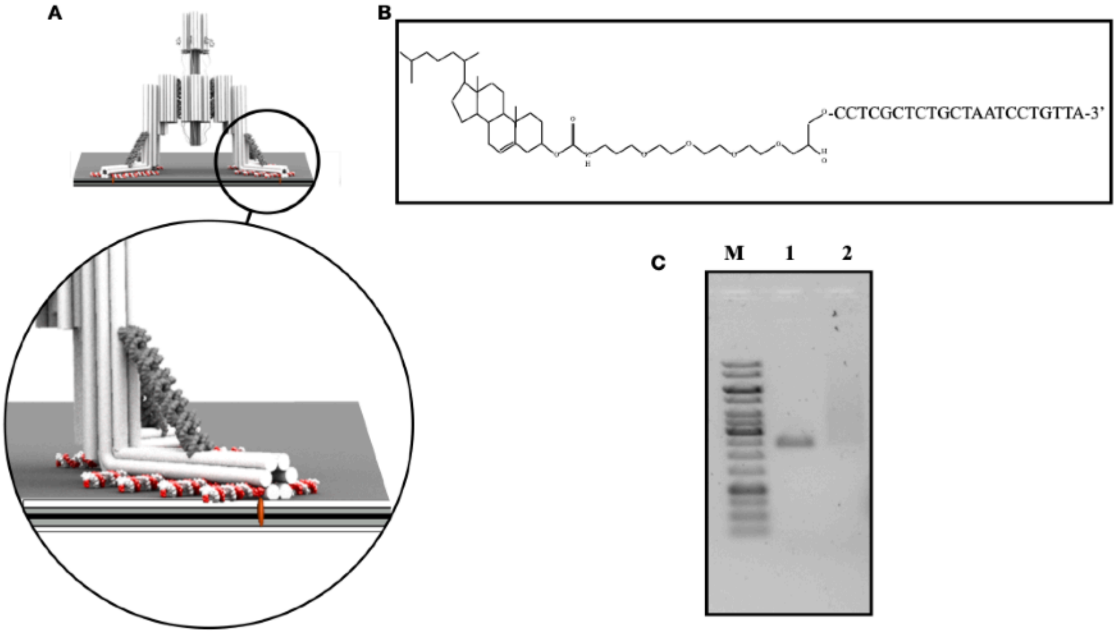

Supplementary Fig. 15. Cholesterol functionalization of the Nano-winch. A) Cholesterol-functionalized oligonucleotides (red strands with red oval) anneal to anchors (grey) on the underside of the Landing Leg. **B)** Structure and sequence of the cholesterol-oligonucleotide. C) Landing Leg origami with anchor strands, lane 1, incubated with 200nM of cholesterol-oligonucleotide for 30 minutes at 37°C resulted in hindered migration of the origami, lane 2. Samples were visualized on 1% agarose gel in 0.5XTBE buffer and 11mM $MgCl_2$. Uncropped and unprocessed scans of all the gels are provided in the source data.

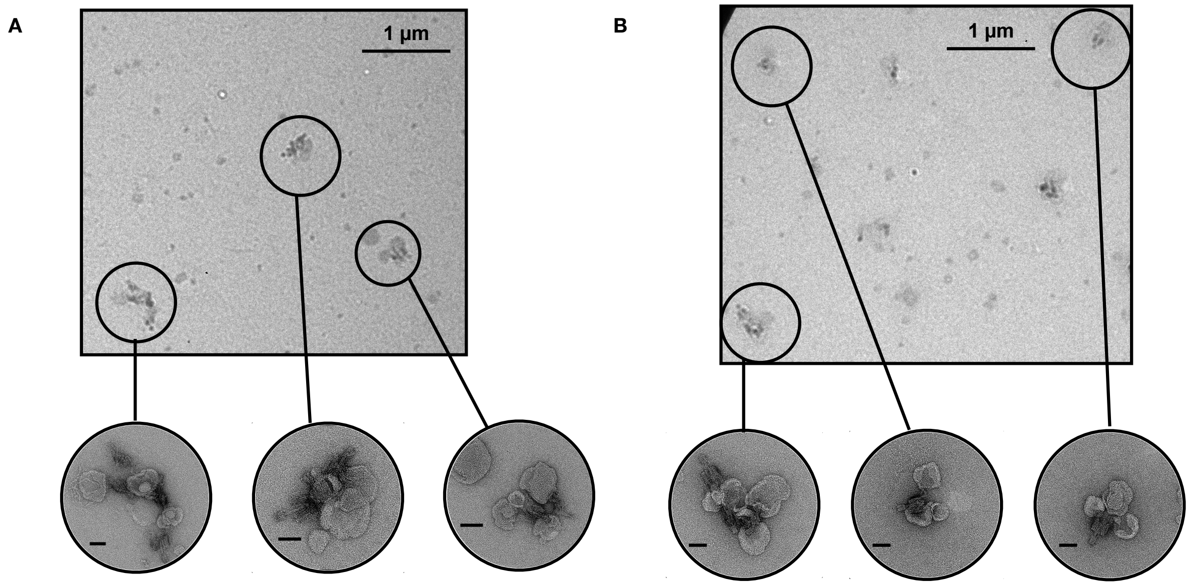

Supplementary Fig. 16. Nano-winch landing on small unilamellar vesicles (SUVs). Cholesterol- functionalized Nano-winches landed on POPC SUVs in both **A)** and **B)**. Black bars represent 50nm.

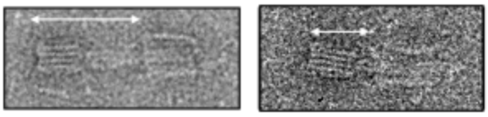

Measurement	Extended	Closed
1	55	26
2	50	26
3	49	24
4	49	22
5	49	23
6	53	28
7	54	31
8	52	27
9	54	30
10	53	25
Mean Distance (nm)	52	26
Standard Deviation (nm)	2	3

Supplementary Fig. 17. Measurement error analysis. Piston-cylinders were measured ten times each in the extended or contracted state to derive a mean and standard deviation from ImageJ analysis.

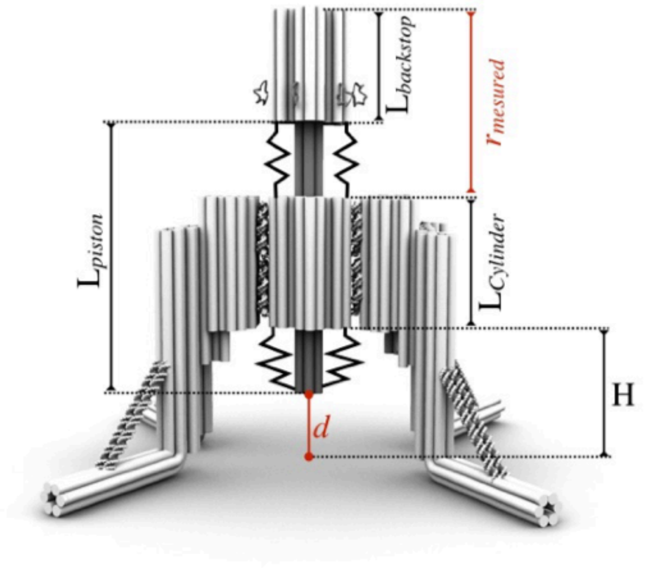

Supplementary Fig. 18. Piston-cylinder Measurements. Precise measurements of the domains of the Piston- cylinder were performed from the top of the backstop to the top of the cylinder. The length of the backstop was then subtracted from the measurement, *X*, to determine the distance extended, *d*.

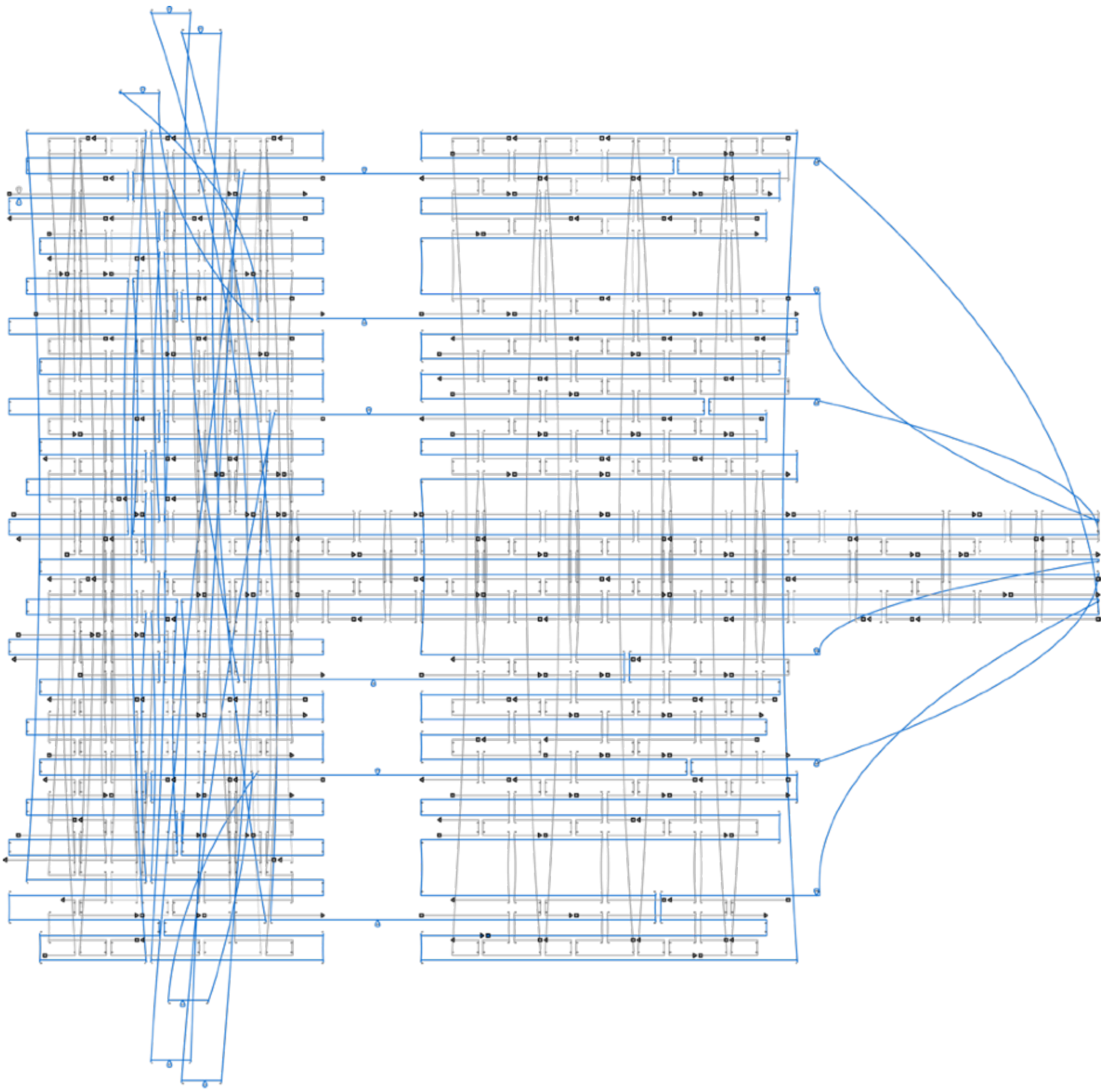

Supplementary Fig. 19. caDNAno design diagram of the 60-nt Connector Piston-cylinder. The single- stranded scaffold is shown in blue and staples in grey.

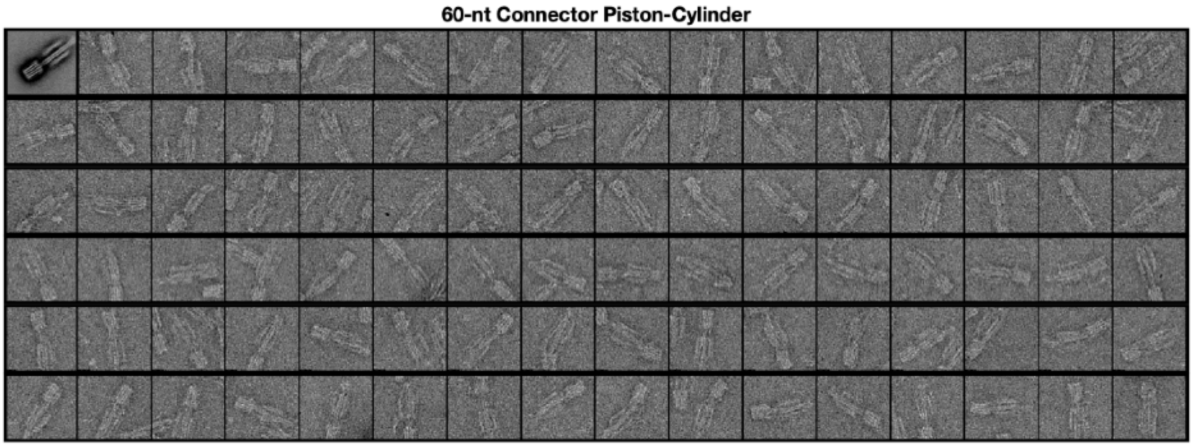

Supplementary Fig. 20. 60-nt Connector Piston-cylinder Particle Set. Representative set of particles of individual 60-nt connector piston-cylinder origami from TEM micrographs. The top left square is an average image. Each square is 92nm x 92nm.

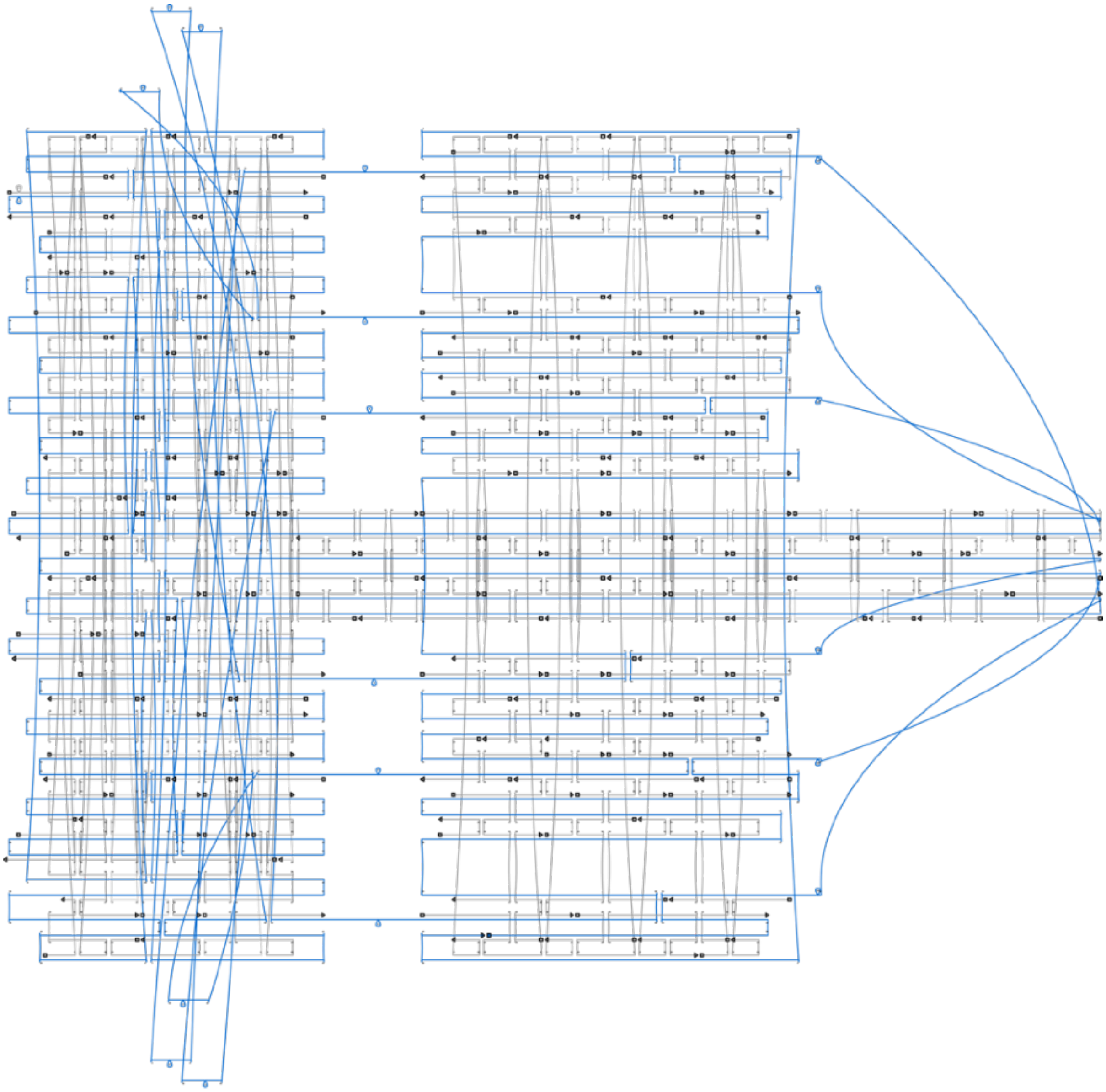

Supplementary Fig. 21. caDNAno design diagram of the 30-nt Connector Piston-cylinder. The single- stranded scaffold is shown in blue and staples in grey.

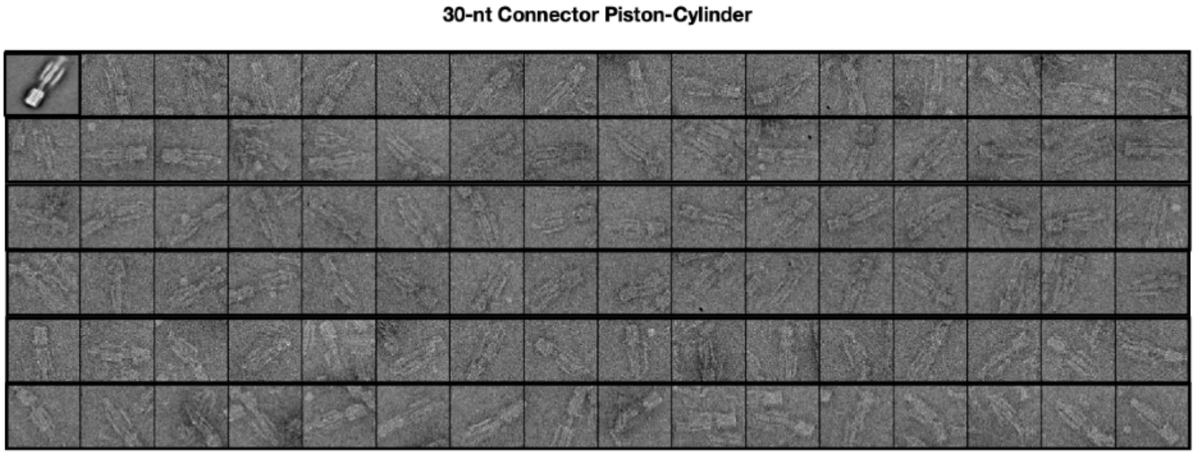

Supplementary Fig. 22. 30-nt Connector Piston-cylinder Particle Set. Representative set of particles of individual 30-nt connector piston-cylinder origami from TEM micrographs. The top left square is an average image. Each square is 92nm x 92nm.

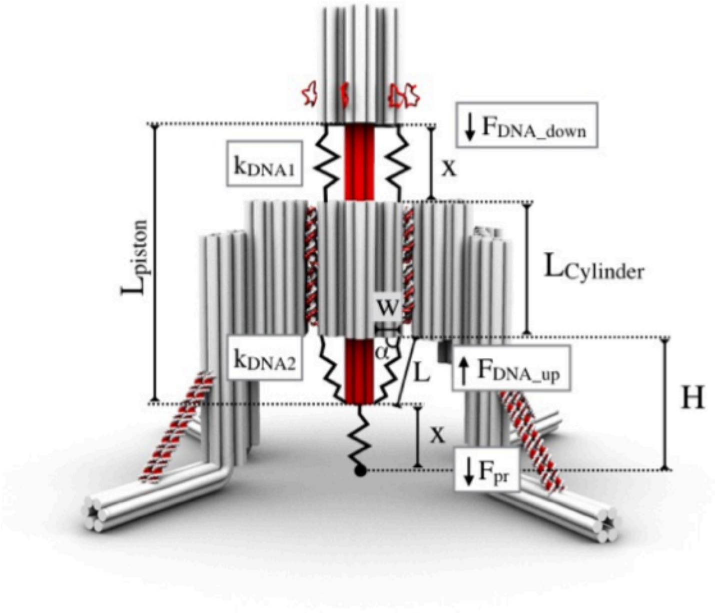

Supplementary Fig. 23. Geometric model of the nanomachine.

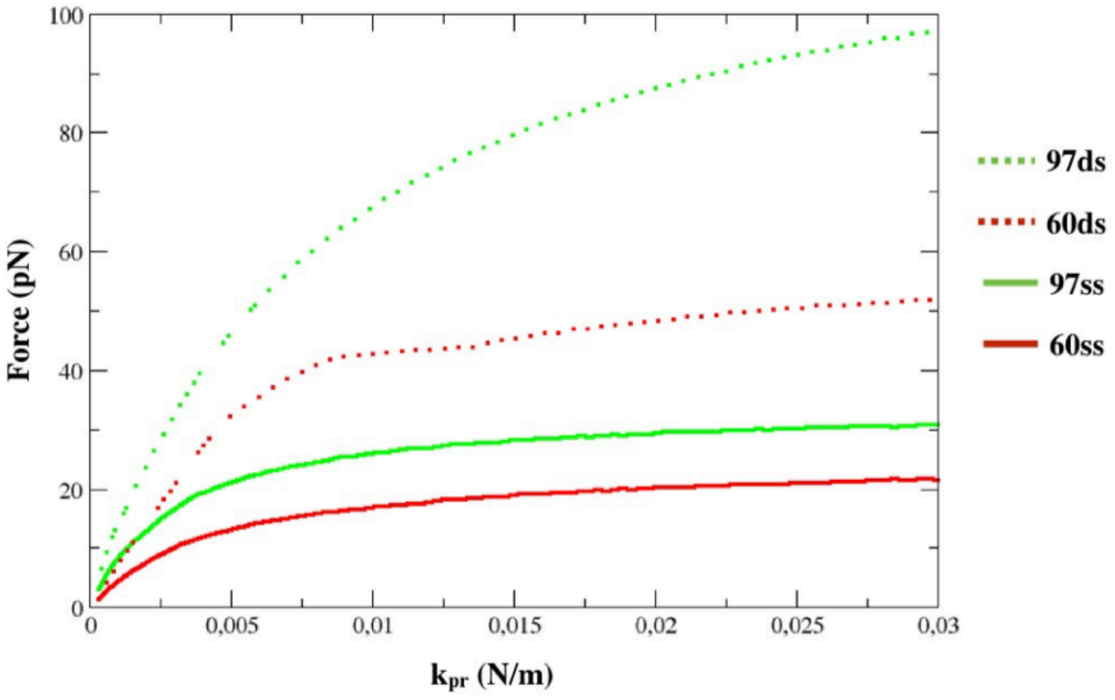

Supplementary Fig. 24. Resulting force, pN, as a function of kpr spanning typical values for protein molecular systems. In green and red the Nano-winch assembled with 97 and 60 connector loops respectively.

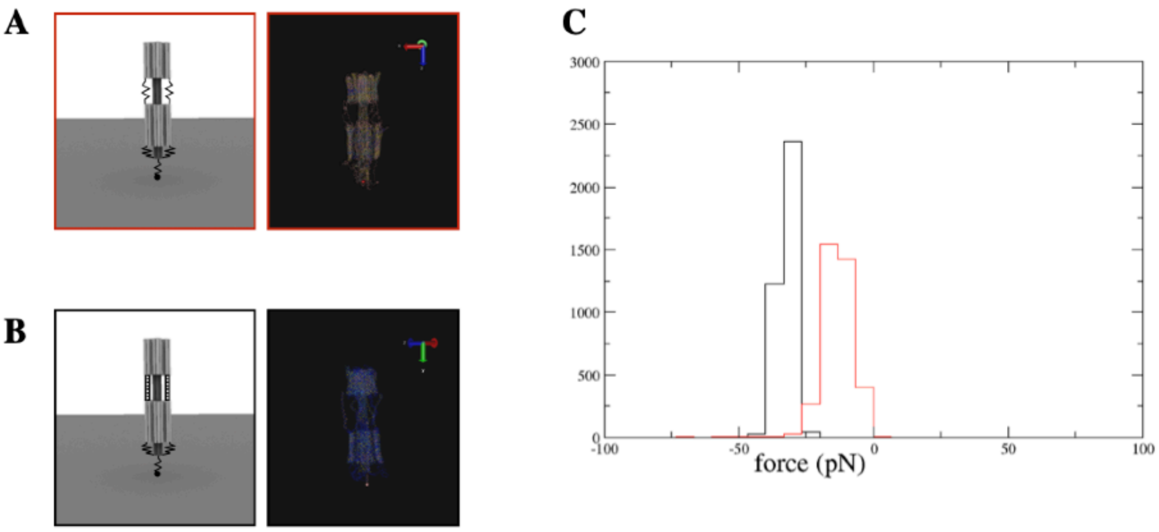

Supplementary Fig. 25. Histograms of the force applied by the nanomachine. A simulation of the Piston- cylinder suspended above a membrane surface with A) single-stranded, and B) double-stranded connector configurations. C) Average force extension profiles of the Piston-cylinder with single- stranded connectors (red) and double-stranded (black) K_{pr}(oxDNA) = 0.0057 (N/m)

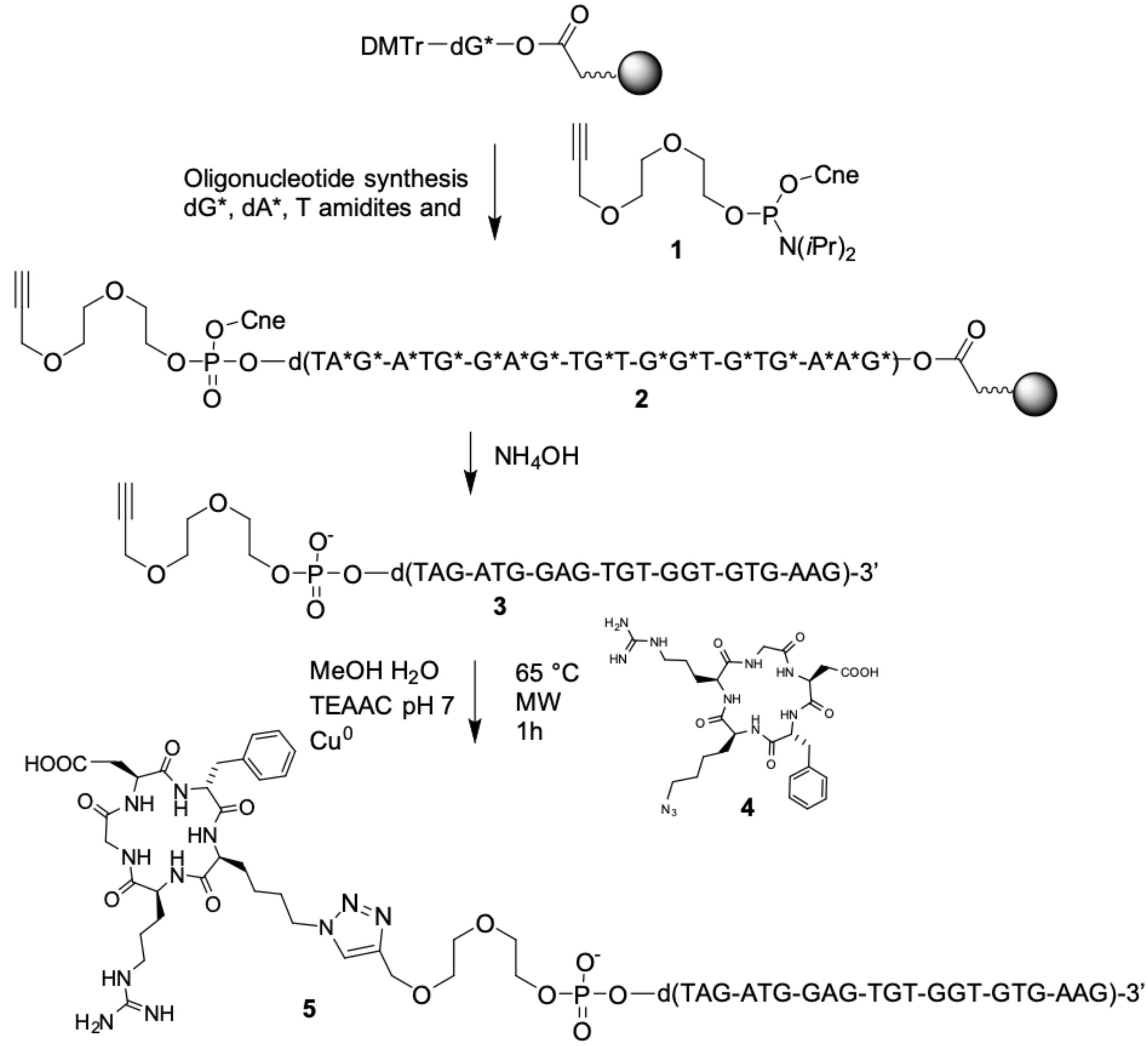

Supplementary Fig. 26. Synthesis of the RGD-oligonucleotide.

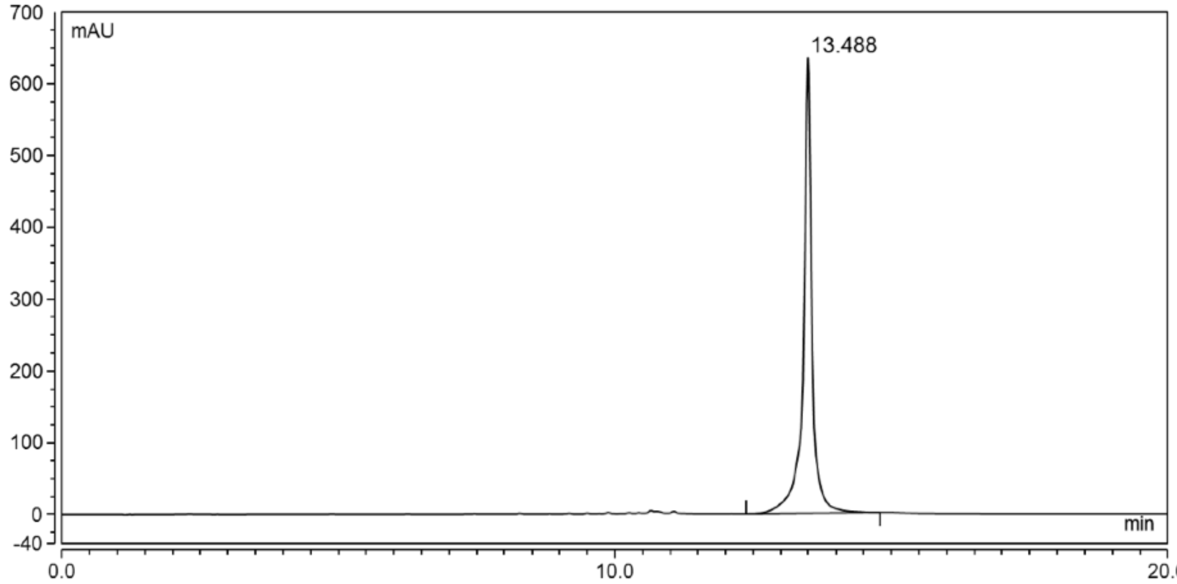

Supplementary Fig. 27. HPLC profile of the synthesised RGD-oligonucleotide. HPLC done on a Macherey Nagel Nucleodur C18 HTec column (length: 250 mm, ID: 10 mm), using a linear gradient from 4 to 17 % of CH3CN in 50 mM TEAAc pH 7 for 20 min.

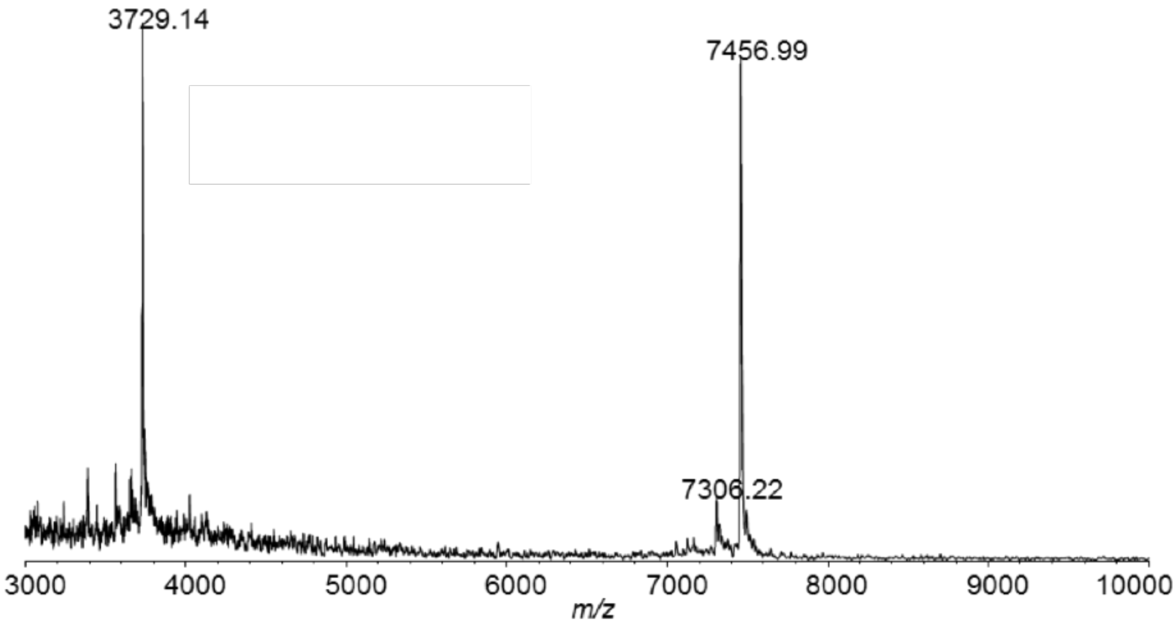

Supplementary Fig. 28. MALDI-TOF profile of the synthesised RGD-oligonucleotide. MS: m/z: $[M-H]^-$: for $C_{244}H_{308}N_{98}O_{137}P_{21}$ calcd.: 7455.15; found: 7456.99.

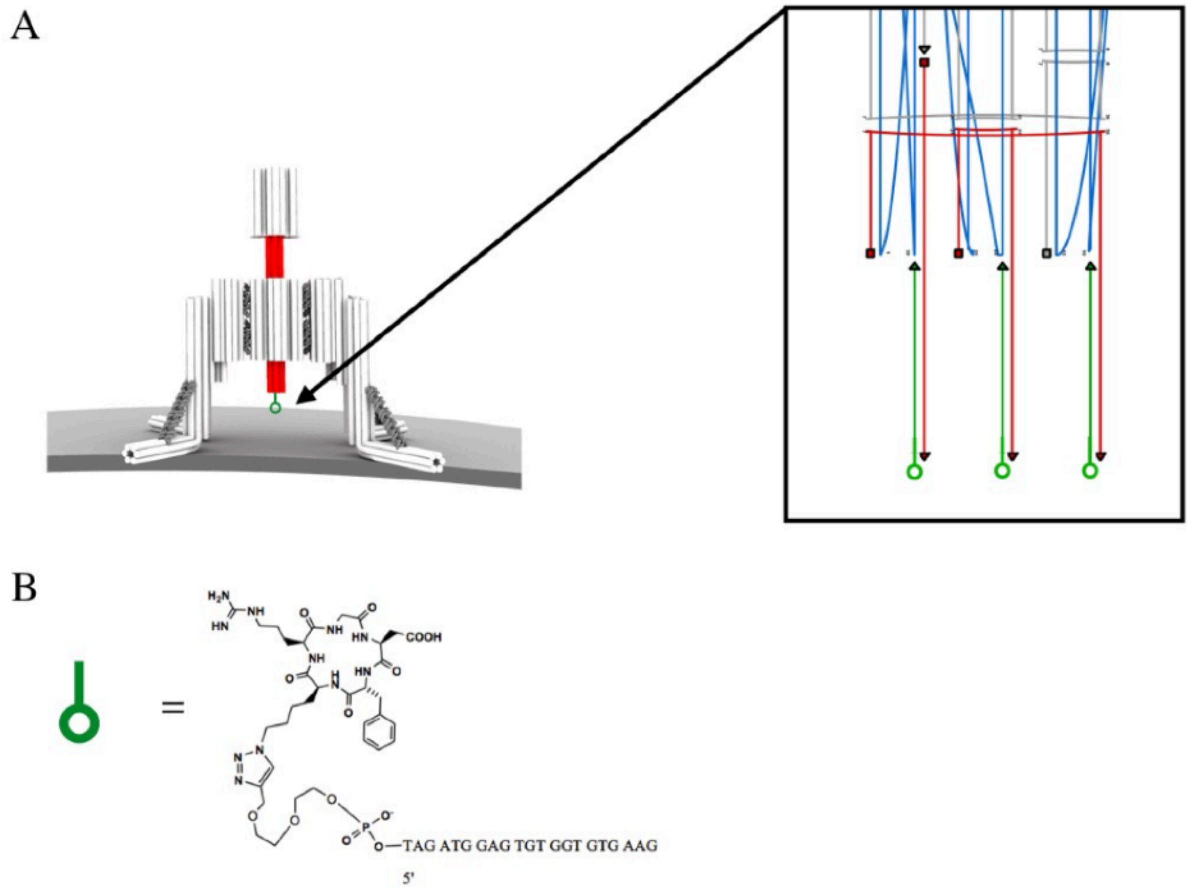

Supplementary Fig. 29. Functionalized Piston tip. A) The six helix bundle tip of the piston of the Nano-winch functionalized with three ligands. **B)** Cyclic RGD-conjugated oligonucleotides (green) to anneal on the piston tip.

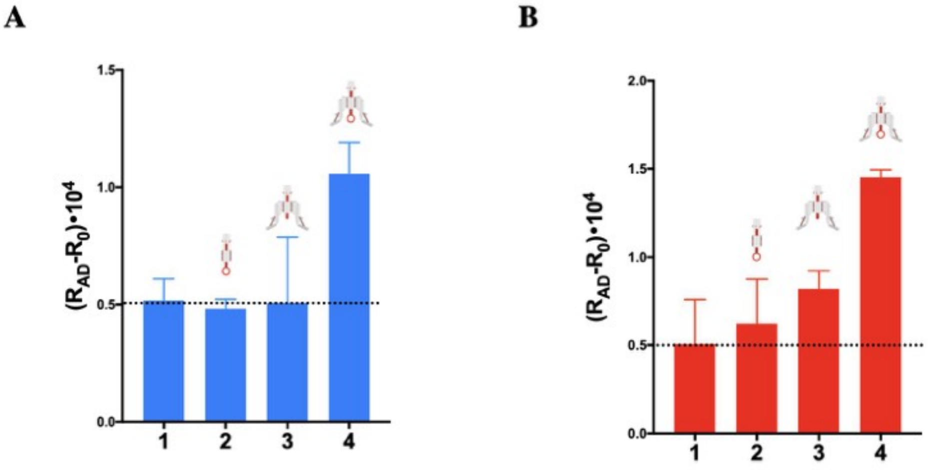

Supplementary Fig. 30. Optimal Cell Density. Cyclic-RGD functionalized Nano-winches were incubated with different densities of MCF-7 cells in suspension. Densities of 10,000 (**A**) to 20,000 cells (**B**) per plate were evaluated for integrin stimulation through FAK phosphorylation. Columns represent mean value (1, base-line. 2, piston-cylinder cRGD. 3, Nano-winch without cRGD. 4, Nano-winch with cRGD.) with bars representing standard deviation of a triplicate. Source data are provided as a Source Data file.

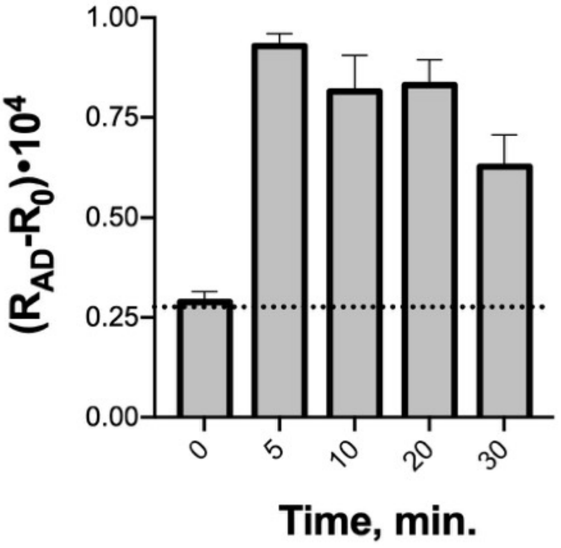

Supplementary Fig. 31. Kinetics of Nano-winch induced integrin activation. MCF-7 cells in suspension were incubated with cRGD-functionalized Nano-winches over a 30 minute time course. Columns represent mean value with bars representing standard deviation of a triplicate. Source data are provided as a Source Data file.

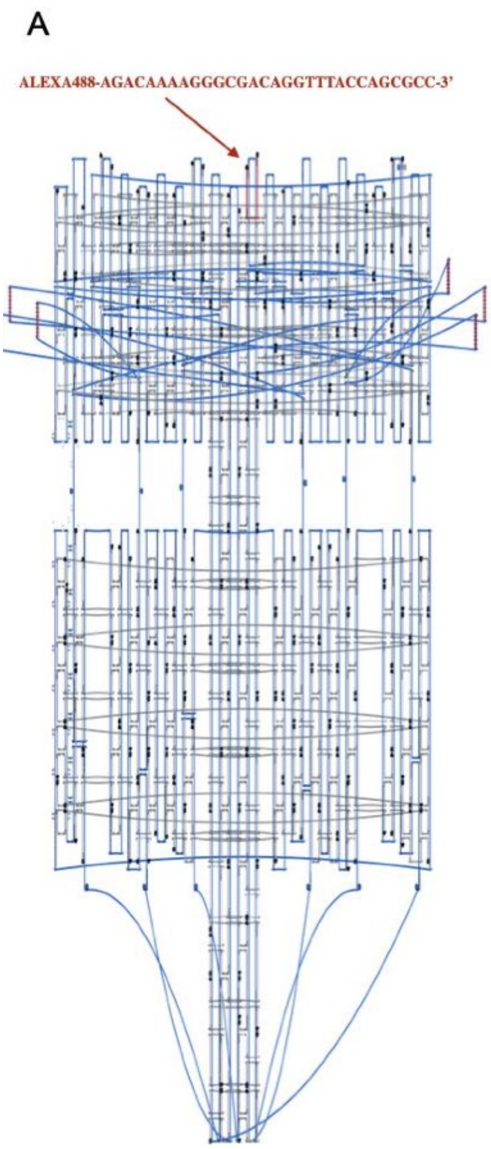

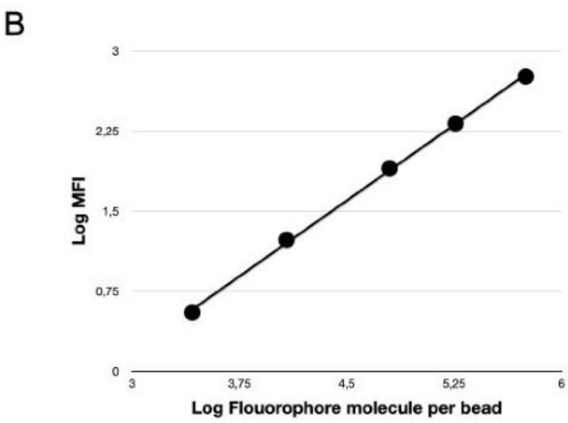

C

Number fluorophore per bead	Measured MFI	Coefficient variation of fluorescent peak	Measured Log MFI	Calculated number fluorophores per bead
2,500	3.61	16.12	0.55	2,600
13,000	16.54	17.56	1.23	12,600
63,000	77.56	16.98	1.9	64,000
181,000	205.81	17.34	2.32	178,800
525,000	579.12	16.54	2.76	529,800

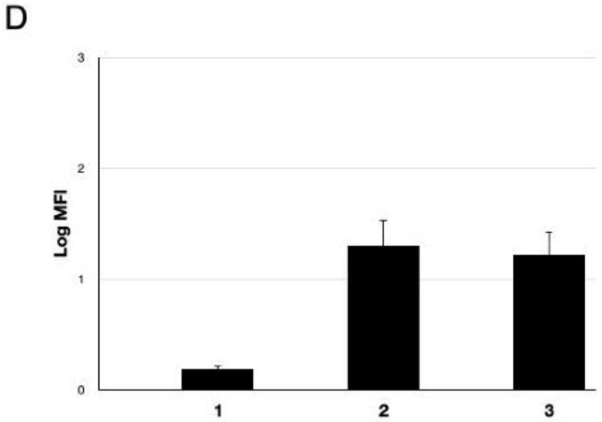

Supplementary Fig. 32. Flow cytometry quantification of Nano-winch bound to a MCF-7 cell. (A) CadNano design of the Alexa Fluor 488 oligonucleotides (red strands) anneal to anchors on the backstop of the Nano-winch. (B) Linear regression analysis of log number of fluorophore molecules per bead and observed MFI, with the fitted trendline overlaid (R^2 = 0.997). (C) Fluorescence intensities of the QIFIKIT five calibration bead populations measured using a BD Biosciences flow cytometer equipped with a 488 nm argon laser. (C) logMFI in triplicate of 1, fluorescently labeled Nano-winch without RGD, 2, fluorescently labeled Nano-winch with RGD after 15 minutes incubation time with MCF-7 cells and 3, fluorescently labeled Nano-winch with RGD after 30 minutes incubation time with MCF-7 cells. Source data are provided as a Source Data file.

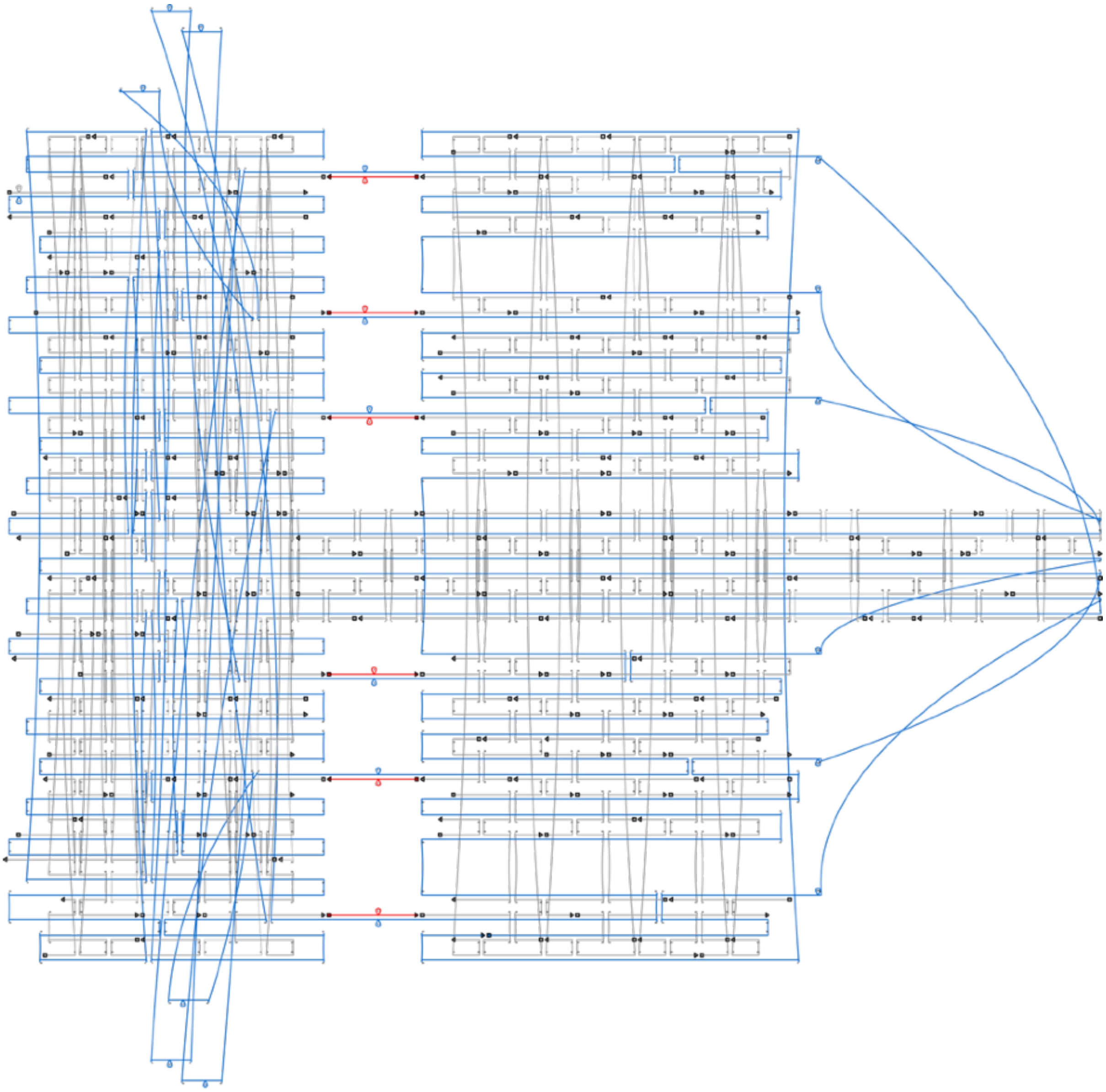

Supplementary Fig. 33. caDNAno design diagram of the extended 30-nt Connector Piston-cylinder. The single-stranded scaffold is shown in blue and staples in grey.

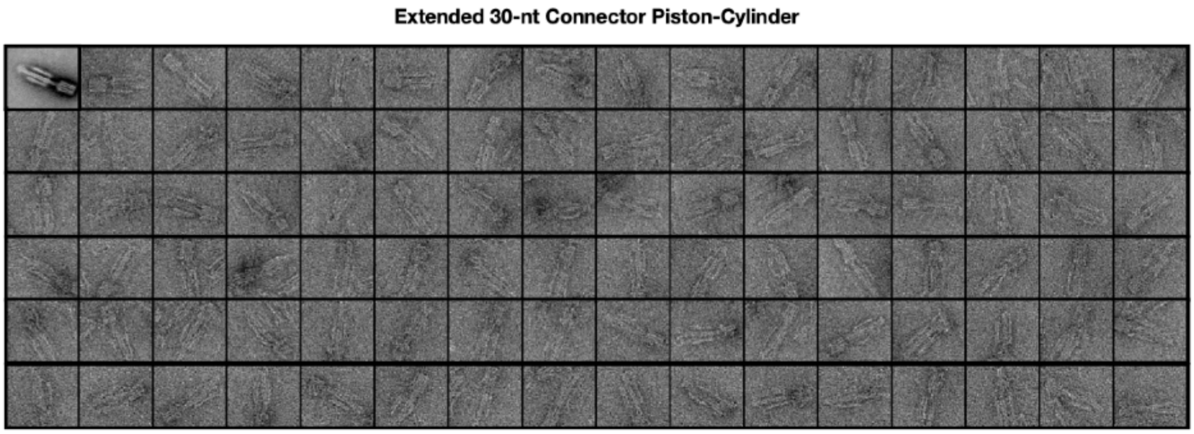

Supplementary Fig. 34. Extended 30-nt Connector Piston-cylinder Particle Set. Representative set of particles of individual extended 30-nt connector piston-cylinder origami from TEM micrographs. The top left square is an average image. Each square is 92nm x 92nm.

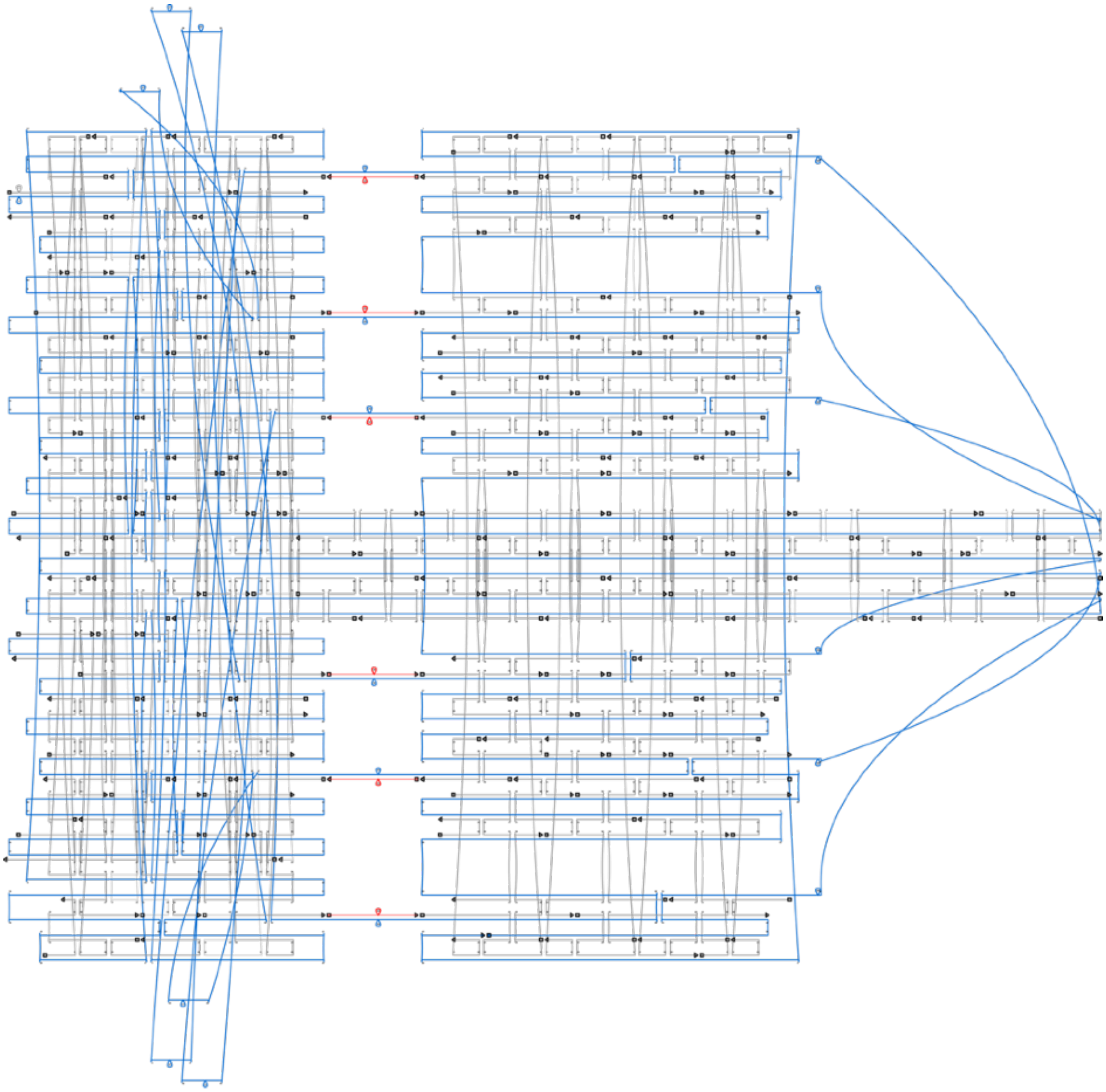

Supplementary Fig. 35. caDNAno design diagram of the extended 60-nt Connector Piston-cylinder. The single-stranded scaffold is shown in blue and staples in grey.

Extended 60-nt Connector Piston-Cylinder

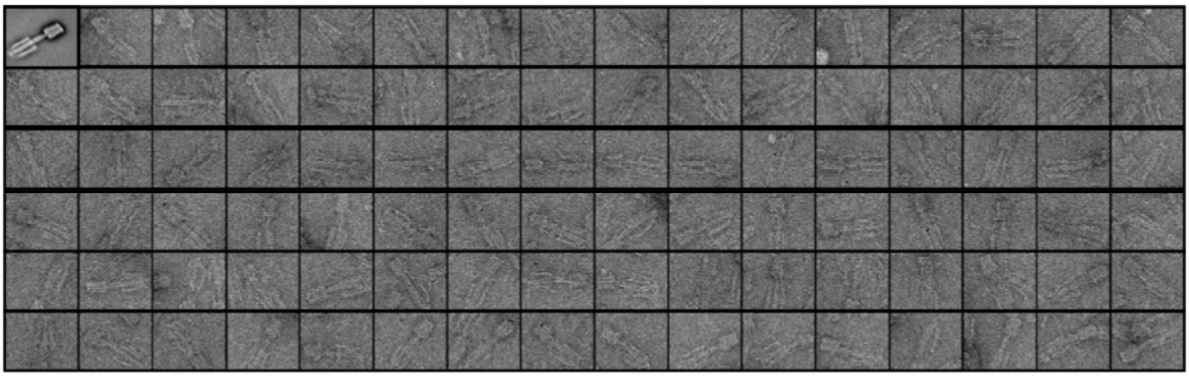

Supplementary Fig. 36. Extended 60-nt Connector Piston-cylinder Particle Set. Representative set of particles of individual extended 60-nt connector piston-cylinder origami from TEM micrographs. The top left square is an average image. Each square is 92nm x 92nm.

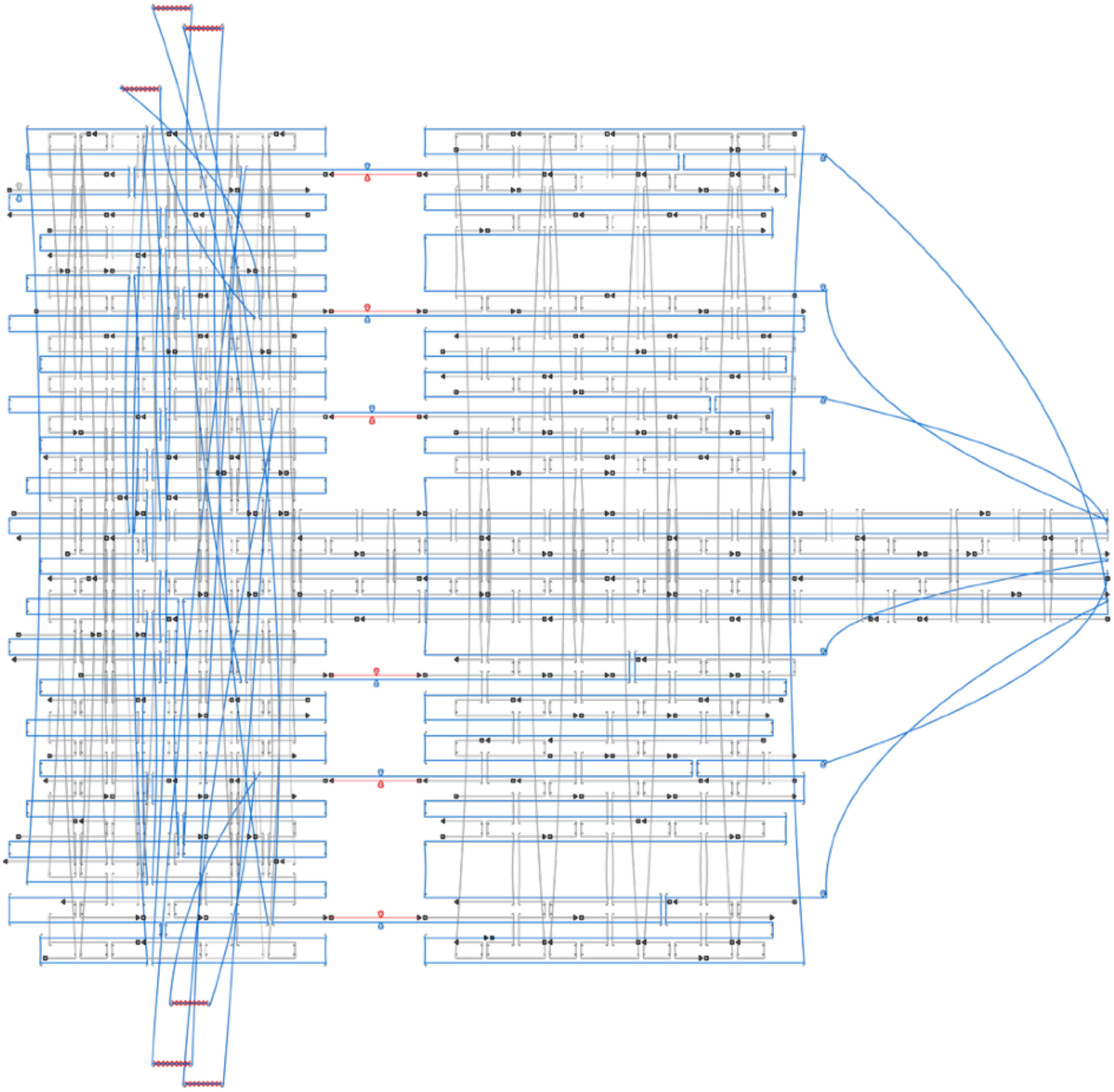

Supplementary Fig. 37. caDNAno design diagram of the extended 97-nt Connector Piston-cylinder. The single-stranded scaffold is shown in blue and staples in grey.

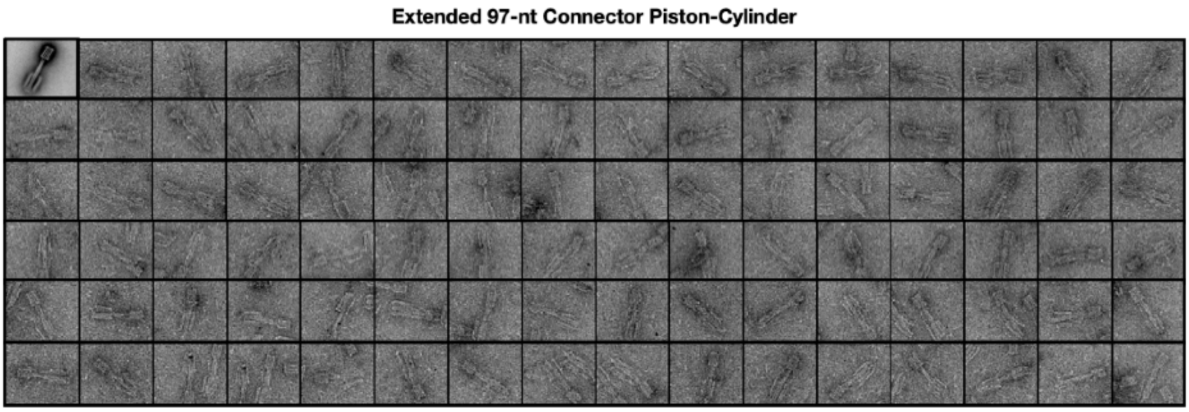

Supplementary Fig. 38. Extended 97-nt Connector Piston-cylinder Particle Set. Representative set of particles of individual extended 97-nt connector piston-cylinder origami from TEM micrographs. The top left square is an average image. Each square is 92nm x 92nm.

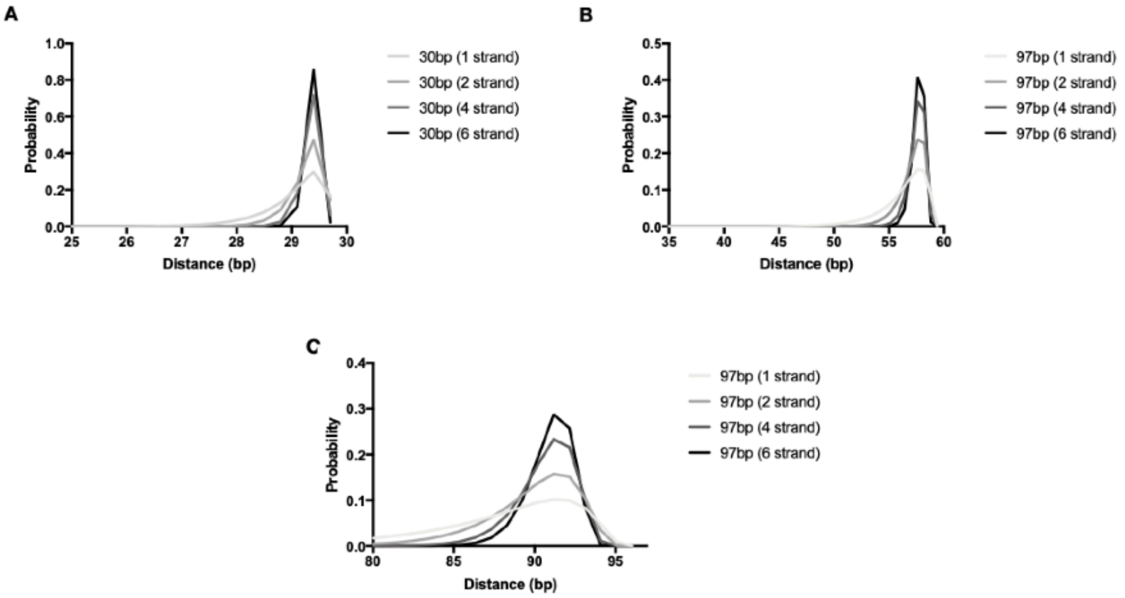

Supplementary Fig. 39. Theoretical End-to-End Distance of Extension. Effective end-to-end distance extension of the Piston-Cylinder dependent on the number of dsDNA connector strands using the WLC model predicted for the **A)** 30bp, **B)** 60bp, and **C)** 97bp.

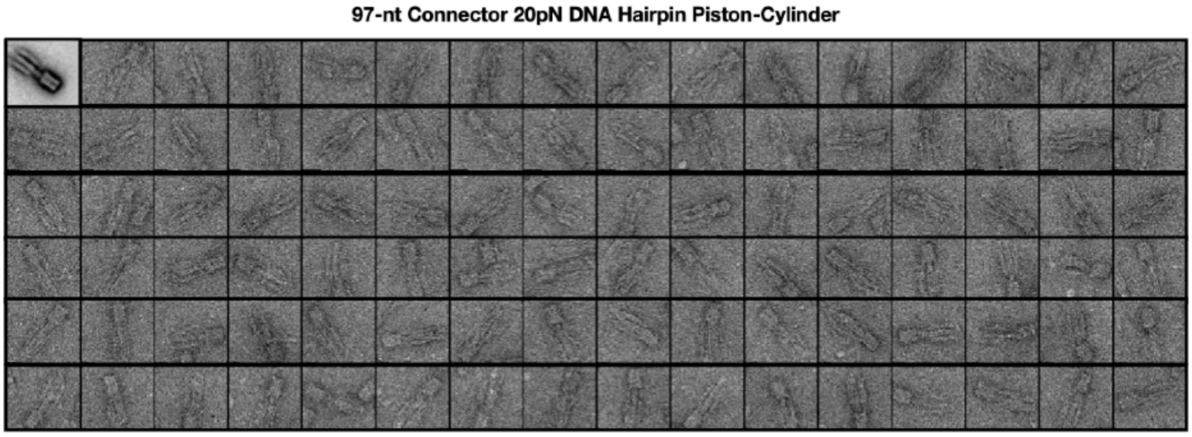

Supplementary Fig. 40. 97-nt Connector Piston-cylinder with 20pN DNA Hairpin Particle Set. Representative set of particles of individual 97-nt connector piston-cylinder origami with $F_{1/2}$ 20pN DNA hairpin from TEM micrographs. The top left square is an average image. Each square is 92nm x 92nm.

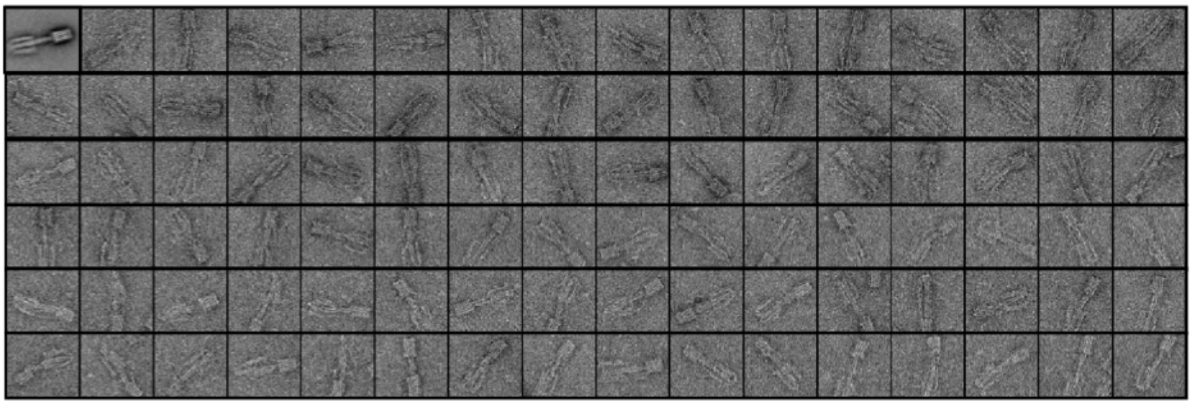

Supplementary Fig. 41. Extended 97-nt Connector with 20pN DNA Hairpin Piston-cylinder Particle Set. Representative set of particles of individual 97-nt connector piston-cylinder origami with $F_{1/2}$ 20pN DNA hairpin from TEM micrographs. The top left square is an average image. Each square is 92nm x 92nm.

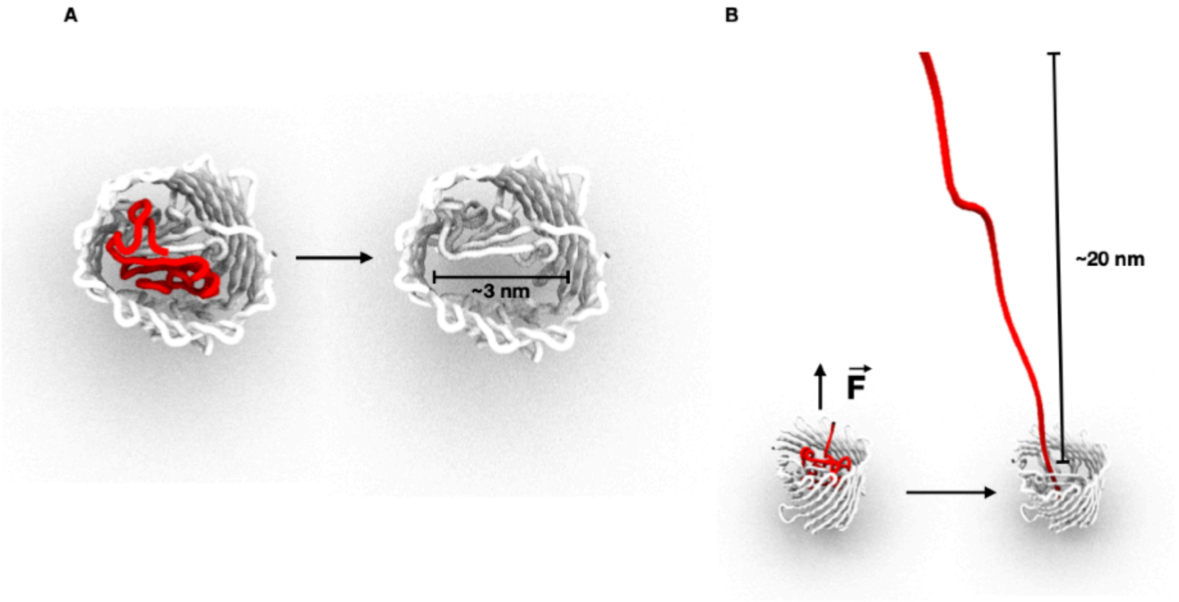

Supplementary Fig. 42. BtuB Channel. Wild-type BtuB is a β-barrel occluded by a plug domain. A) Top view of the BtuB protein (1NQG.pdb, 15). Coloured in red, the first 49 residues directly downstream of the Ton box motif. This motif is known to unfold first as these residues are initially exposed to mechanical deformation as force propagates through the plug. In grey, the recalcitrant plug subdomain. B) The length of a stretched amino acid is 0.4 nm; therefore, force applied to the plug domain has been shown to result in the partial unfolding of the plug domain and extension up to approximately ~20 nm (Hickman et al. 2017).

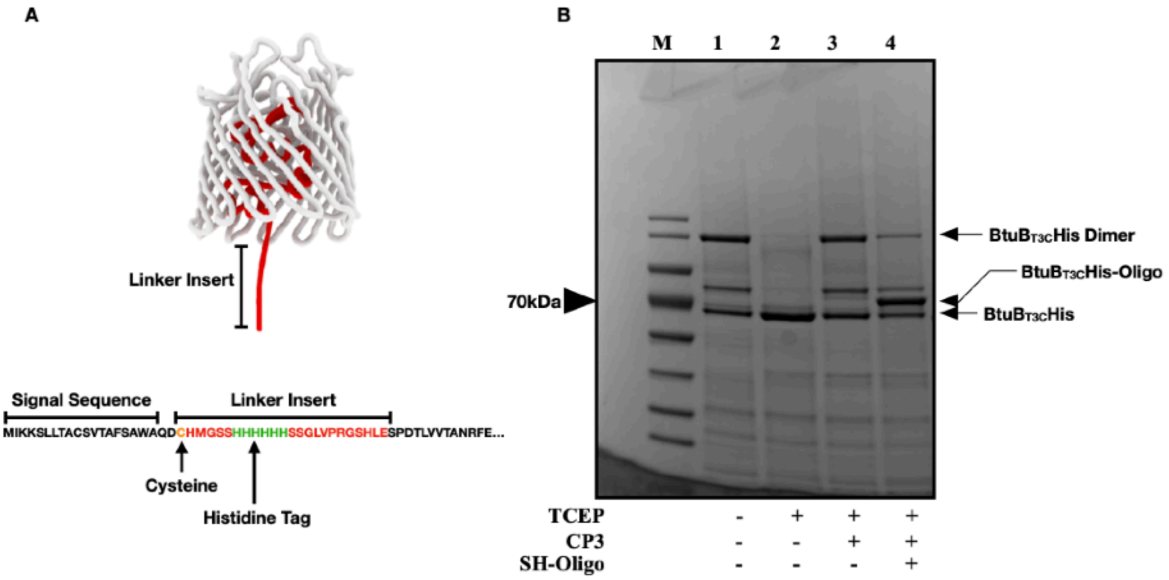

Supplementary Fig. 43. BtuBT3cHis conjugation. A) BtuB was engineered with an N-terminal linker insert to extend the N-terminus. In the amino-acid sequence a linker insert (red text) containing a 6-His- tag (green text) and a cysteine substitution (orange text) was inserted into the wild-type sequence (black text) after the signal sequence. **B)** Conjugation of thiolated-oligonucleotide with BtuBT3cHis analyzed on 4-20% SDS-PAGE. Lane 1, BtuBT3cHis after purification, lane 2, BtuBT3cHis after incubation with 0.6mM TCEP for 10 minutes, lane 3, 10 minute incubation of BtuBT3cHis with 1.4mM copper phenanthroline after 0.6mM TCEP treatment, lane 4, incubation of BtuBT3cHis with thiolated-oligonucleotide during treatment with 0.6mM TCEP, then 1.4mM copper phenanthroline. Uncropped and unprocessed scans of all the gels are provided in the source data.

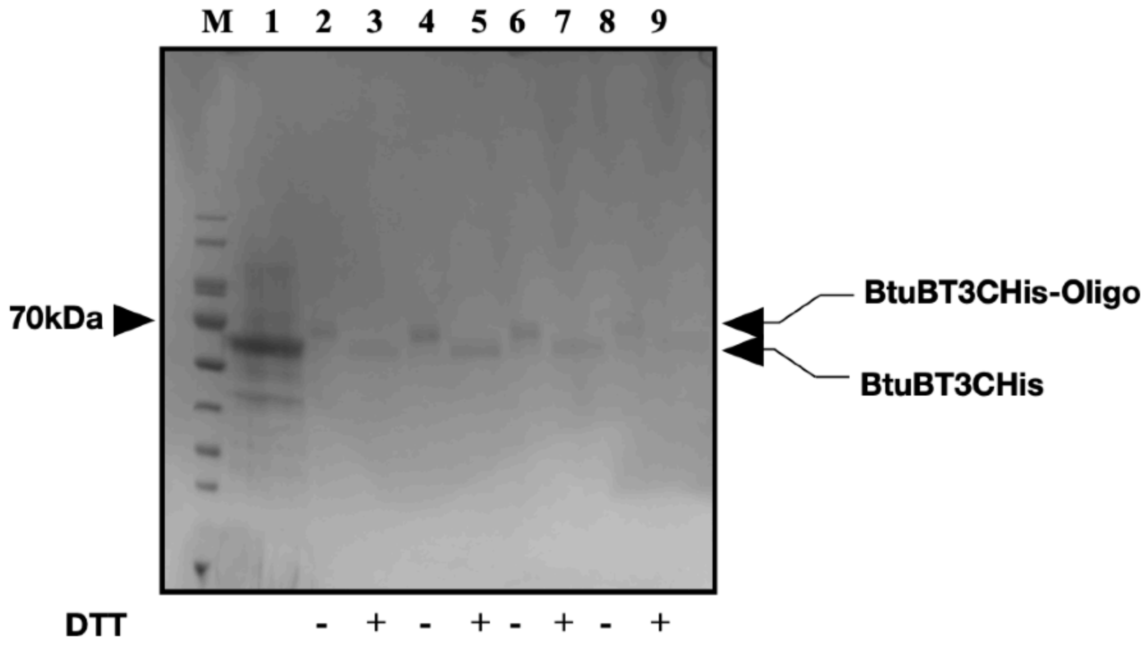

Supplementary Fig. 44. Size exclusion of BtuB$_{T3C}$His-oligonucleotide. BtuB$_{T3C}$His-oligonucleotide was separated from excess oligonucleotide using a Superdex 200 HR 10/30 column. Fractions were analyzed by 4-20% SDS-PAGE. Lane 1, BtuB$_{T3C}$His start material prior to conjugation with thiolated oligonucleotide. Lanes 2 to 9, fractions 12 to 14 alternating with and without incubation with DTT prior to migration on SDS-PAGE. Uncropped and unprocessed scans of all the gels are provided in the source data.

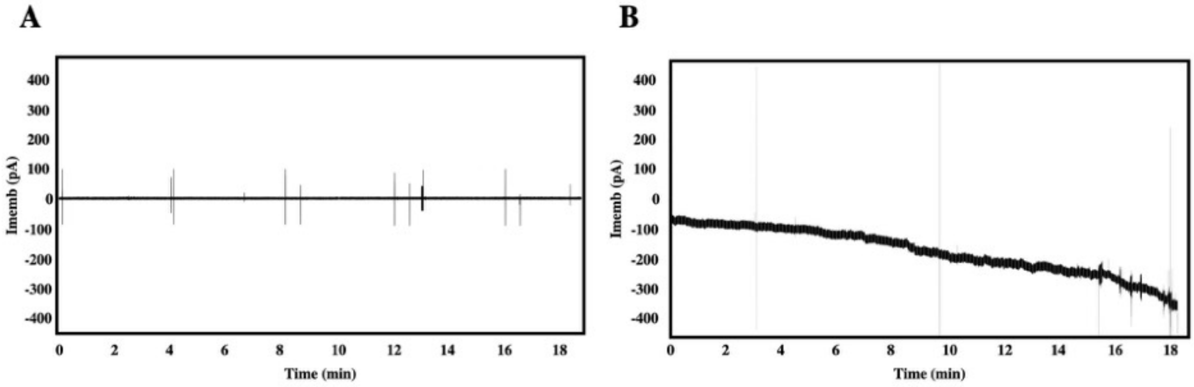

Supplementary Fig. 45. BtuB$_{T3C}$His-oligo in Planar Lipid Bilayer. Current traces of conjugated BtuB$_{T3C}$His- oligo applied to planar lipid bilayer with **A**, buffer only or **B**, with 4M urea in solution. Gradually increasing conductivity observed after the unfolding of the BtuB plug domain. Conditions were held at -20mV.

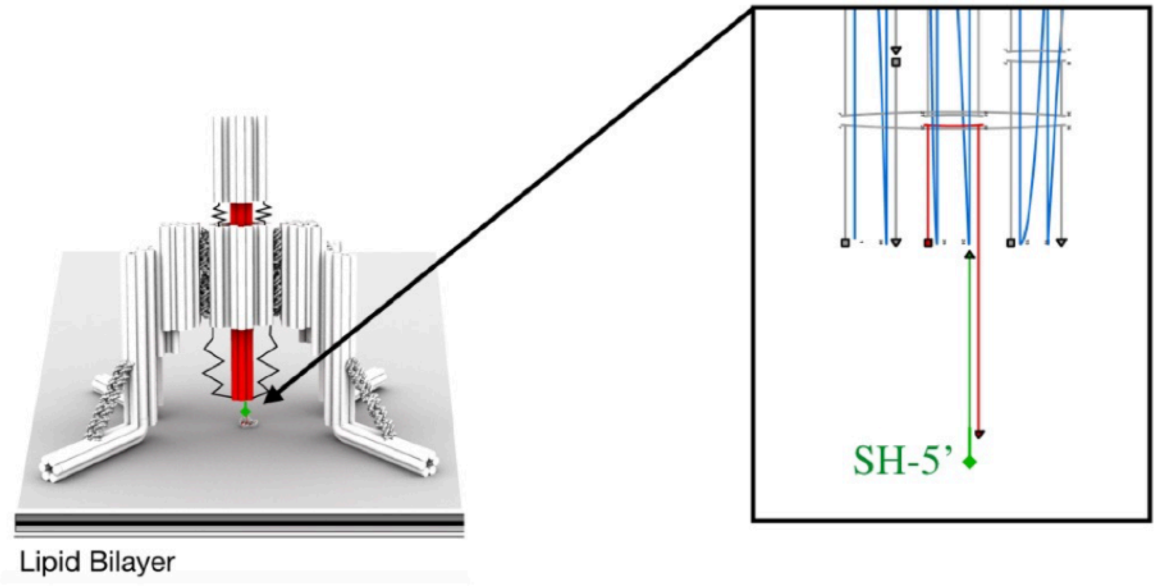

Supplementary Fig. 46. Targeting Thiolated Membrane Protein. A membrane protein conjugated with a thiolated oligonucleotide (green) is targeted by a complementary anchor strand (red) emerging from the tip of the Nano-winch piston. The Nano-winch is then able to directly apply tension upon the membrane protein.

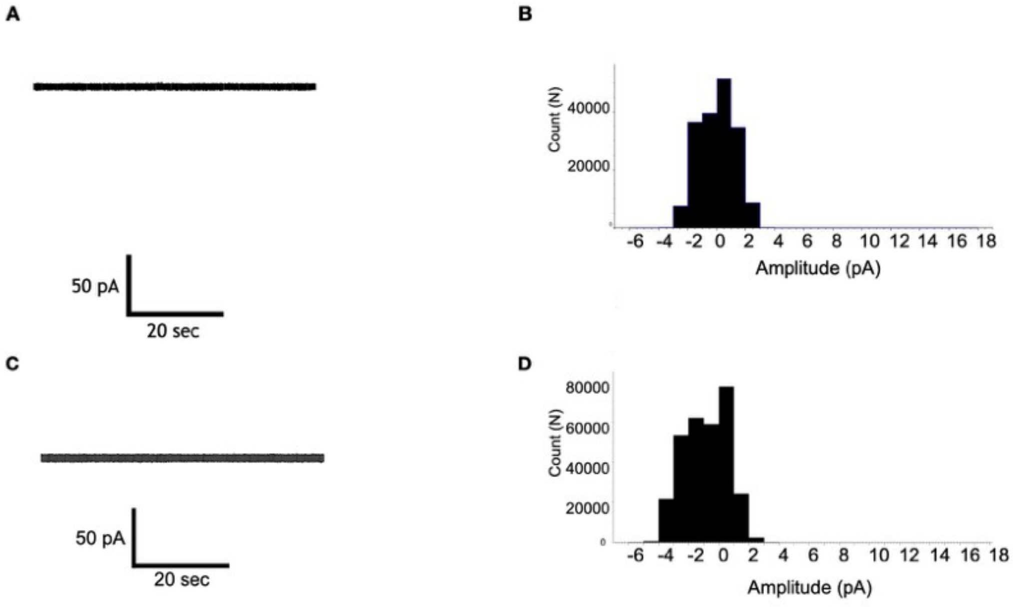

Supplementary Fig. 47. BtuB does not open without extension oligonucleotides. Representative traces of BtuB$_{T3C}$His-oligo reconstituted into planar lipid bilayer with A, Nano-winch without extension oligonucleotides with B, corresponding trace count. BtuB$_{T3C}$His-oligo reconstituted into planar lipid bilayers with C, ~400nM extension oligos and D, the corresponding trace count. Conditions were held at 50mV throughout the duration of the experiment.

References :

1. Smith, S. B., Cui, Y. & Bustamante, C. Overstretching B-DNA: the elastic response of individual double-stranded and single-stranded DNA molecules. *Science* **271**, 795–799 (1996).

2. Chi, Q., Wang, G. & Jiang, J. The persistence length and length per base of single-stranded DNA obtained from fluorescence correlation spectroscopy measurements using mean field theory. *Physica A: Statistical Mechanics and its Applications* **392**, 1072–1079 (2013).

3. Steltenkamp, S., Muller, M.M., Deserno, M., Hennesthal, C., Steinem, C., and Janshoff, A. Mechanical properties of pore-spanning lipid bilayers probed by atomic force microscopy, *Biophys. J.* **91**, 217-226 (2006).

4. Lee, G., K. Abdi, ., P. E. Marszalek. Nanospring behavior of ankyrin repeats. *Nature*. **440**:246–249 (2006).

5. Chen, H., & Yan, J. (2008). Effects of kink and flexible hinge defects on mechanical responses of short double-stranded DNA molecules. *Physical Review E*, *77*(4), 041907.

6. Ouldridge,T.E., Louis, A.A., and Doye, J.P.K. Structural, mechanical and thermodynamic properties of a coarse-grained DNA model. *J. Chem. Phys*., **134**, 085101 (2011).

7. Wilhelm, J., and Frey, E. Radial Distribution Function of Semiflexible Polymers. Phys. Rev. Lett. **77**, 2581 (1996).

8. Becker, N. B., Rosa, A., and Everaers, R. The radial distribution function of worm-like chains. The European Physical Journal E, **32**(1), 53-69 (2010).

9. Funke, JJ., Dietz, H. Placing molecules with Bohr radius resolution using DNA origami. Nat Nanotechnol. **11**(1):47-52 (2016).

10. Janshoff, A., & Steinem, C. Mechanics of lipid bilayers: What do we learn from pore-spanning membranes?. *Biochimica et Biophysica Acta (BBA)-Molecular Cell Research*, **1853**(11), 2977-2983. (2015)

11. Mey, I., Stephan, M., Schmitt, E. K., Müller, M. M., Ben Amar, M., Steinem, C., & Janshoff, A. Local membrane mechanics of pore-spanning bilayers. *Journal of the American Chemical Society*, **131**(20), 7031-7039. (2009)

12. Lee, C., Wei, X., Kysar, J. W., & Hone, J. Measurement of the elastic properties and intrinsic strength of monolayer graphene. *science*, **321**(5887), 385-388. (2008)

13. Wan, K. T., Guo, S., & Dillard, D. A. A theoretical and numerical study of a thin clamped circular film under an external load in the presence of a tensile residual stress. *Thin Solid Films*, **425**(1-2), 150-162. (2003)

14. Begley, M. R., & Mackin, T. J. Spherical indentation of freestanding circular thin films in the membrane regime. Journal of the Mechanics and Physics of Solids, **52**(9), 2005-2023 (2004).

15. Chimento, D. P., Mohanty, A. K., Kadner, R. J., & Wiener, M. C. Substrate-induced transmembrane signaling in the cobalamin transporter BtuB. *Nature Structural & Molecular Biology*, **10**(5), 394-401 (2003).
