# Supplementary material for: A modular spring-loaded actuator for mechanical activation of membrane proteins
Source: Nat Commun. 2022 Jul 28;13:3182. doi: 10.1038/s41467-022-30745-2 (PMC9334261; doi:10.1038/s41467-022-30745-2)
Supplement: Supplementary file 2 — Reporting Summary [file 41467_2022_30745_MOESM2_ESM.pdf]

nature portfolio

Corresponding author(s): Bellot Gaëtan

Last updated by author(s): May 11, 2022

Reporting Summary

Nature Portfolio wishes to improve the reproducibility of the work that we publish. This form provides structure for consistency and transparency in reporting. For further information on Nature Portfolio policies, see our Editorial Policies and the Editorial Policy Checklist.

Statistics

For all statistical analyses, confirm that the following items are present in the figure legend, table legend, main text, or Methods section.

n/a	Confirmed	
☐	☒	The exact sample size (*n*) for each experimental group/condition, given as a discrete number and unit of measurement
☐	☒	A statement on whether measurements were taken from distinct samples or whether the same sample was measured repeatedly
☒	☐	The statistical test(s) used AND whether they are one- or two-sided<br>*Only common tests should be described solely by name; describe more complex techniques in the Methods section.*
☐	☒	A description of all covariates tested
☐	☒	A description of any assumptions or corrections, such as tests of normality and adjustment for multiple comparisons
☐	☒	A full description of the statistical parameters including central tendency (e.g. means) or other basic estimates (e.g. regression coefficient) AND variation (e.g. standard deviation) or associated estimates of uncertainty (e.g. confidence intervals)
☒	☐	For null hypothesis testing, the test statistic (e.g. *F*, *t*, *r*) with confidence intervals, effect sizes, degrees of freedom and *P* value noted<br>*Give P values as exact values whenever suitable.*
☐	☒	For Bayesian analysis, information on the choice of priors and Markov chain Monte Carlo settings
☐	☒	For hierarchical and complex designs, identification of the appropriate level for tests and full reporting of outcomes
☒	☐	Estimates of effect sizes (e.g. Cohen's *d*, Pearson's *r*), indicating how they were calculated

Our web collection on statistics for biologists contains articles on many of the points above.

Software and code

Policy information about availability of computer code

Data collection	Gatan DigitalMicrograph 3.5
Data analysis	caDNAno version 2.0 was used to design the DNA structures.<br>Eman2 version 2.3 was used for two-dimensional class averaging<br>ImageJ version 2.0 was used to measure distributions of the different DNA origami nanostructures as described in the Methods section.

For manuscripts utilizing custom algorithms or software that are central to the research but not yet described in published literature, software must be made available to editors and reviewers. We strongly encourage code deposition in a community repository (e.g. GitHub). See the Nature Portfolio guidelines for submitting code & software for further information.

Data

Policy information about availability of data

All manuscripts must include a data availability statement. This statement should provide the following information, where applicable:

- Accession codes, unique identifiers, or web links for publicly available datasets
- A description of any restrictions on data availability
- For clinical datasets or third party data, please ensure that the statement adheres to our policy

The authors declare that the source data supporting the findings of this study are available within the paper and its Supplementary information files. Source data are provided with this paper.

Field-specific reporting

Please select the one below that is the best fit for your research. If you are not sure, read the appropriate sections before making your selection.

☒ Life sciences ☐ Behavioural & social sciences ☐ Ecological, evolutionary & environmental sciences

For a reference copy of the document with all sections, see nature.com/documents/nr-reporting-summary-flat.pdf

Life sciences study design

All studies must disclose on these points even when the disclosure is negative.

Sample size	For experiments involving quantification, at least three independent experiments were chosen as the minimal replicate number, and sample size was determined by statistical significance with comparison to untreated control.
Data exclusions	No data were excluded from analysis.
Replication	All experiments were reproduced to reliably support conclusions stated in the manuscript.
Randomization	TEM imaging were selected randomly, all images were analyzed equally with no sub-sampling.
Blinding	Blinding was not possible as experimental conditions were evident from the image data. Quantifications were performed using computational pipeline applied equally to all conditions and replicates.

Reporting for specific materials, systems and methods

We require information from authors about some types of materials, experimental systems and methods used in many studies. Here, indicate whether each material, system or method listed is relevant to your study. If you are not sure if a list item applies to your research, read the appropriate section before selecting a response.

Materials & experimental systems

n/a	Involved in the study	
☐	☒	Antibodies
☐	☒	Eukaryotic cell lines
☒	☐	Palaeontology and archaeology
☒	☐	Animals and other organisms
☒	☐	Human research participants
☒	☐	Clinical data
☒	☐	Dual use research of concern

Methods

n/a	Involved in the study	
☒	☐	ChIP-seq
☐	☒	Flow cytometry
☒	☐	MRI-based neuroimaging

Antibodies

Antibodies used	anti-FAK, PerkinElmer. Manufacturer reference : Perkin Elmer LLC 64FAKPEH
Validation	anti-FAK antibody has been validated by PerkinElmer by demonstrating immunoblotting on Human MCF-7 and HeLa cells

Eukaryotic cell lines

Policy information about cell lines

Cell line source(s)	MCF-7 cells, (human, atcc htb-22)
Authentication	None of the cell lines have been authenticated.
Mycoplasma contamination	Cell lines were tested negative for mycoplasma contamination and no indication of contamination was observed.
Commonly misidentified lines (See ICLAC register)	No commonly misidentified cell lines were used.

Flow Cytometry

Plots

Confirm that:

- [x] The axis labels state the marker and fluorochrome used (e.g. CD4-FITC).
- [x] The axis scales are clearly visible. Include numbers along axes only for bottom left plot of group (a 'group' is an analysis of identical markers).
- [x] All plots are contour plots with outliers or pseudocolor plots.
- [x] A numerical value for number of cells or percentage (with statistics) is provided.

Methodology

Sample preparation	MCF-7 cells (Michigan Cancer Foundation – 7) were grown in RPMI supplemented with 10% FBS (without antibiotics) at 37°C, 5% CO2. Cells were detached with 5ml Versene 1X solution (Gibco) for 10-15 minutes at 37°C, 5% CO2. After adding 5ml RPMI, cells were centrifuged for five minutes at 1,400 rpm. The cell pellet was resuspended in RPMI in order to have the required number of cells. 100 μL MCF-7 cells at 1 × 10^5 cells/mL suspended in RPMI were mixed for 10 minutes at 37°C with fluorescently labeled Nano-winch at saturating concentrations in triplicate, with controls (cells only, fluorescently labeled Nano-winch without cRGD and Nano-winch without Alexa Fluor 488-labeled DNA staple strand). Subsequently, cells were washed twice with 10 mM PBS before it was measured using a flow cytometer. Samples were analyzed by flow cytometry using BD Biosciences, at least 10.000 events were acquired per sample. Collected data were processed by BD FACSSuite (BD Biosciences).
Instrument	BD FACScanto II (BD biosciences) equipped with 488nm, 633nm and 405nm lasers.
Software	BD FACSDiVa (Becton Dickinson biosciences)
Cell population abundance	Cell sorting was not relevant and not employed. All flow cytometry data regard only sample analysis.
Gating strategy	Using the FSC/SSC gating, debris was removed by gating on the cell population. The boundaries between "positive" and "negative" staining cells were gated according to the control groups.

- [x] Tick this box to confirm that a figure exemplifying the gating strategy is provided in the Supplementary Information.
